# Deletion of *SNX9* alleviates CD8 T cell exhaustion for effective cellular cancer immunotherapy

**DOI:** 10.1038/s41467-022-35583-w

**Published:** 2023-02-02

**Authors:** Marcel P. Trefny, Nicole Kirchhammer, Priska Auf der Maur, Marina Natoli, Dominic Schmid, Markus Germann, Laura Fernandez Rodriguez, Petra Herzig, Jonas Lötscher, Maryam Akrami, Jane C. Stinchcombe, Michal A. Stanczak, Andreas Zingg, Melanie Buchi, Julien Roux, Romina Marone, Leyla Don, Didier Lardinois, Mark Wiese, Lukas T. Jeker, Mohamed Bentires-Alj, Jérémie Rossy, Daniela S. Thommen, Gillian M. Griffiths, Heinz Läubli, Christoph Hess, Alfred Zippelius

**Affiliations:** 1 Laboratory of Cancer Immunology, Department of Biomedicine, University of Basel and University Hospital of Basel, Basel, Switzerland; 2 Laboratory of Tumor Heterogeneity, Metastasis and Resistance, Department of Biomedicine, University of Basel and University Hospital of Basel, Basel, Switzerland; 3 Laboratory of Immunobiology, Department of Biomedicine, University of Basel and University Hospital of Basel, Basel, Switzerland; 4 Cambridge Institute for Medical Research, Biomedical Campus, Cambridge, CB2 0XY UK; 5 Laboratory of Cancer Immunotherapy, Department of Biomedicine, University of Basel and University Hospital of Basel, Basel, Switzerland; 6 Bioinformatics Core Facility, Department of Biomedicine, University of Basel and University Hospital of Basel, Basel, Switzerland; 7 Swiss Institute of Bioinformatics, Basel, Switzerland; 8 Laboratory of Molecular Immune Regulation, Department of Biomedicine, University of Basel and University Hospital of Basel, Basel, Switzerland; 9 Transplantation Immunology & Nephrology, Basel University Hospital, Basel, Switzerland; 10 Department of Surgery, University Hospital Basel, Basel, Switzerland; 11 Biotechnology Institute Thurgau, University of Konstanz, Kreuzlingen, Switzerland; 12 Division of Molecular Oncology and Immunology, The Netherlands Cancer Institute, Amsterdam, The Netherlands; 13 Medical Oncology, University Hospital Basel, Basel, Switzerland; 14 Cambridge Institute of Therapeutic Immunology and Infectious Disease, Jeffrey Cheah Biomedical Centre, University of Cambridge, Cambridge, CB2 0AW UK

**Keywords:** Translational research, Tumour immunology, Immunotherapy, Tumour immunology

## Abstract

Tumor-specific T cells are frequently exhausted by chronic antigenic stimulation. We here report on a human antigen-specific ex vivo model to explore new therapeutic options for T cell immunotherapies. T cells generated with this model resemble tumor-infiltrating exhausted T cells on a phenotypic and transcriptional level. Using a targeted pooled CRISPR-Cas9 screen and individual gene knockout validation experiments, we uncover sorting nexin-9 (SNX9) as a mediator of T cell exhaustion. Upon TCR/CD28 stimulation, deletion of *SNX9* in CD8 T cells decreases PLCγ1, Ca^2+^, and NFATc2-mediated T cell signaling and reduces expression of NR4A1/3 and TOX. *SNX9* knockout enhances memory differentiation and IFNγ secretion of adoptively transferred T cells and results in improved anti-tumor efficacy of human chimeric antigen receptor T cells in vivo. Our findings highlight that targeting SNX9 is a strategy to prevent T cell exhaustion and enhance anti-tumor immunity.

## Abstract (web summary)

The efficacy of T-cell-based cancer immunotherapies can be compromised by T cell exhaustion. Here the authors develop a human ex vivo exhaustion model and, based on a CRISPR-Cas9 screen, identify SNX9 as a regulator of T cell exhaustion, showing that *SNX9* knockout is associated with improved T cell function and anti-tumor activity in preclinical cancer models.

## Introduction

Tumor-specific CD8 T cells regularly enter a state of exhaustion due to chronic antigen stimulation within the tumor microenvironment^1,2^. T cell exhaustion is characterized by impaired production of effector cytokines such as IFNγ^3,4^ and high expression of inhibitory receptors such as PD-1 and TIM-3^5^. The pivotal role of tumor-specific CD8 T cells in anti-tumor immunity has fueled the development of therapeutic strategies that prevent or revert tumor-associated T cell exhaustion. Antibodies targeting inhibitory receptors such as PD-1 and CTLA4 are now considered to be among the most critical advances in the field of oncology in the last decades, given their outstanding clinical success^6,7^. While PD-1/PD-L1 blockade can reinvigorate some T cells, terminal exhaustion still limits the therapeutic efficacy of immune checkpoint blockade (ICB)^8,9^. Adoptive transfer of tumor-infiltrating lymphocytes (TILs) and genetically engineered T cells such as chimeric antigen receptor (CAR) T cells are changing the landscape of available treatments in hematological malignancies and solid tumors such as melanoma and lung cancer^10^. Despite impressive clinical results, the efficacy of adoptively transferred T cells can likewise be compromised by an exhausted state^11–14^. Thus, most patients with advanced cancers treated with immunotherapies still fail to achieve long-term responses.

The advent of single-cell technologies has re-shaped our understanding of the tumor-infiltrating immune compartment including exhausted T cells in human patients^15–18^. Yet, these studies mainly provide a descriptive snapshot, while the mechanistic understanding and validation of potential targets for immunotherapy remain difficult. Most attempts to screen panels of genes or molecules for their impact on T cell exhaustion are based on murine T cells that were exhausted either in vivo by chronic infection^19^ or in tumor models^20,21^, ex vivo by repetitive stimulation with anti-CD3 antibodies, antigen-expressing tumor cells^22–24^, or stimulation under hypoxia^25^. Interestingly, one attempt to impose an exhaustion state in human T cells utilized repetitive stimulation ex vivo with peptide-loaded human dendritic cells, which resulted in a reduction in cytokine secretion, but not degranulation or killing capacity^26^. Recently, ex vivo models have been developed using human tumor explants, which preserve many important features of the tumor microenvironment and mimic patient response^27,28^. These patient-derived cultures, however, are short-lived with a scarce infiltration of tumor-specific T cells, which hampers extensive screening approaches. Likewise, the low abundance and unknown antigen specificity in TILs freshly obtained from patients hinder more comprehensive mechanistic studies.

To address these challenges, here we develop a human ex vivo exhaustion model to generate tumor antigen-specific exhausted T cells that resemble patient-derived T cells on a phenotypic and transcriptional level. Using this model, we perform a targeted pooled CRISPR-Cas9 screen to rank candidate genes and investigate the highest-ranking genes in single gene validation experiments. We thereby discover that a knockout of sorting nexin-9 (SNX9) improves T cell effector functions and memory differentiation, translating into enhanced anti-tumor efficacy of adoptively transferred T cells including CAR T cells in vivo. Our findings suggest that SNX9 amplifies TCR/CD28-mediated activation through PLCγ1, Ca^2+^, and NFATc2 and this correlates with higher expression of NR4A1/3 and TOX. We thereby identify a therapeutic strategy to improve T cell-based immunotherapies by limiting excessive stimulatory signals and thereby alleviate T cell exhaustion.

## Results

### Ex vivo repetitive antigen-specific stimulation of T cells results in exhaustion

**Fig. 1:**
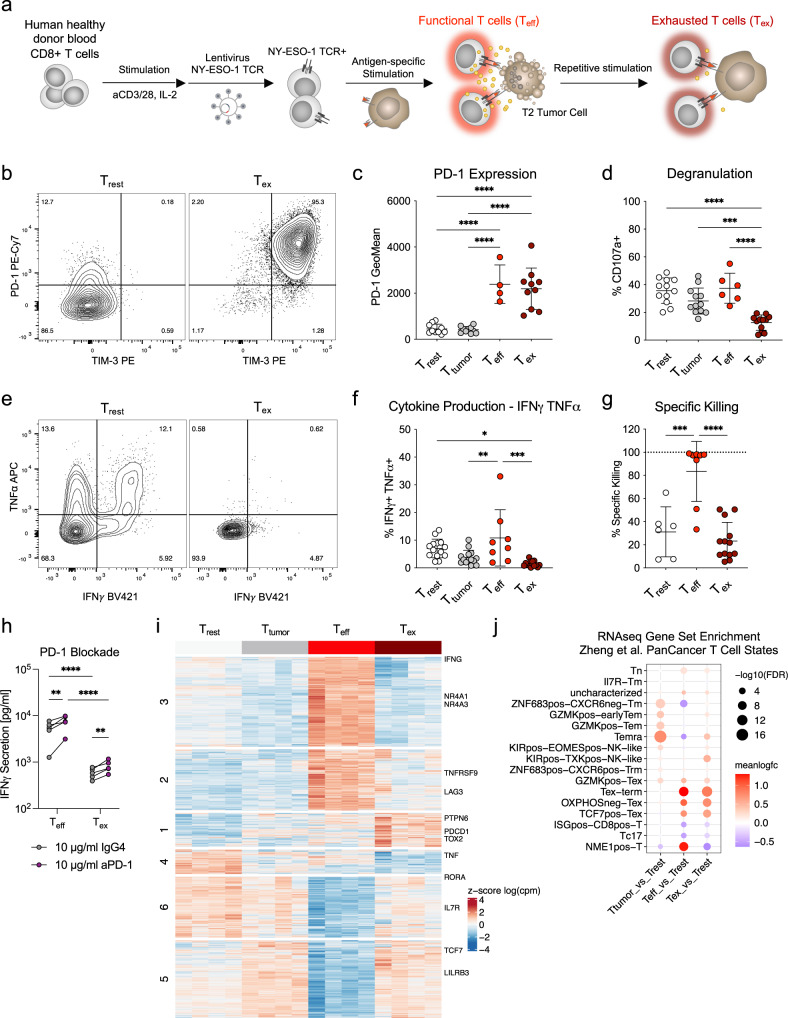
Repetitive antigen-specific stimulation ex vivo of T cells results in exhaustion. **a** Scheme representing the principle of the exhaustion model. **b** Representative co-expression of PD-1 and TIM-3 measured by antibody staining and flow cytometry. **c** PD-1 expression measured by flow cytometry after antibody staining on day 12 after the first stimulation. *n* = 10 donors from *n* = 5 experiments except for T_eff_ *n* = 4 of *n* = 2 experiments. **d** Degranulation measured by CD107a surface exposure during re-stimulation of all cells after 13 days of culture. *n* = 12 donors of *n* = 6 experiments except for T_eff_ *n* = 6 of *n* = 3 experiments. **e–f** Representative flow cytometry plot and quantification of IFNγ and TNFα production measured by intracellular cytokine staining. *n* = 14 donors from *n* = 7 experiments except for T_eff_ *n* = 8 of *n* = 4 experiments. **g** Specific lysis of T2-Luc+ tumor cells pulsed with peptide after 5 hours of co-incubation with T cells measured by luminescence intensity. n = 6 donors for T_rest_, *n* = 13 for T_ex_ and *n* = 8 for T_eff_. **h** IFNγ secretion measured by ELISA after 4 days co-culture of T_eff_ or T_ex_ with MDA-MB-231 cells loaded with 100 nM NY-ESO-1-9V peptide in presence of either 10 μg/ml human IgG4 isotype control or anti-PD-1 antibody (Nivolumab). Statistics is a paired 2-way-ANOVA with Holm-Sidak correction with *n* = 5 donors. **i** Heatmap of all differentially regulated genes between the four conditions showing row-scaled log-cpms and clustered using k-means (numbers indicated on the left). Selected genes associated with exhaustion or functionality are labeled on the right. *n* = 4 donors. **j** Gene set enrichment analysis comparing mRNA expression of the different conditions for the Zheng. et al PanCancer T cell states. Color represents the mean log fold change and the size the -log10 false discovery rate (FDR). **c**-**d**; **f**-**g** 1-way ANOVA statistics with Holm-Sidak correction, Mean and SD are shown. * *p* < 0.05, ** *p* < 0.01, *** *p* < 0.001, **** *p* < 0.0001. Source data and exact p-values are provided as a Source Data file.

To develop an antigen-specific ex vivo model for human T cell exhaustion, we transduced human healthy donor peripheral CD8 T cells with a lentiviral construct encoding a TCR specific for the cancer-testis antigen NY-ESO-1^29,30^. We hypothesized that repetitive stimulation with antigen presented on tumor cells would lead to T cell exhaustion. Therefore, we repetitively stimulated transduced antigen-specific T cells using HLA-A2 positive T2 tumor cells loaded with 1 μM NY-ESO-1 peptides for four cycles in 3-day intervals (T_ex_, Fig. 1a). We compared T_ex_ to T cells that only received medium and IL-2 (T_rest_) and to T cells that were co-cultured with T2 tumor cells without peptides (T_tumor_). We also included a condition that is only stimulated once three days before the readout to mimic activated effector T cells (T_eff_, Supplementary Fig. 1a).

Both T_eff_ and T_ex_ had increased expression of the inhibitory receptors PD-1 and TIM-3 compared to T_rest_ and T_tumor_ controls (Fig. 1b, c, Supplementary Fig. 1b, c). However, only T_ex_ showed a decreased capacity to degranulate (CD107a exposure upon restimulation), produce the inflammatory cytokines IFNγ and TNFα, and kill tumor cells (Fig. 1d, g). The co-expression of PD-1, TIM3, and LAG3 and the impairment in degranulation correlated with the peptide concentration used for the repetitive stimulation (Supplementary Fig. 1d). At the same time, IFNγ production was impaired even at the lowest concentration of NY-ESO-1 peptide. T_ex_ maintained increased PD-1 expression and impaired degranulation capacity after seven days of resting without further stimulation (Supplementary Fig. 1e, f). A therapeutic anti-PD1 antibody could improve the killing capacity and IFNγ secretion of both T_eff_ and T_ex_ in a co-culture assay with PD-L1 expressing tumor cells, however, T_ex_ functionality remained impaired (Fig. 1h, Supplementary Fig. 1g). Loss of proliferative capacity has been described as another hallmark of exhaustion^31^. T_ex_ highly expanded during repetitive stimulation, however, their potential to proliferate in response to stimulation was reduced compared to controls (Supplementary Fig. 1h, i). Confirming our results with repetitive T2 lymphoma line stimulation, T_ex_ generated using a melanoma cell line for stimulation (NA8-Mel, HLA-A2+, loaded with NY-ESO-1 peptide) showed a similar exhausted phenotype (Supplementary Fig. 1j). We concluded that T_ex_ generated by repetitive antigen-specific stimulation ex vivo functionally resemble human TILs in their reduced capacity to degranulate, secrete cytokines, and proliferate^2^.

Intratumoral T cells are characterized by a distinct transcriptional pattern compared to their functional counterparts^2,3,32^. We therefore investigated transcriptional changes among the different conditions by bulk mRNA sequencing (Supplementary Fig. 2a, b). Compared to T_rest_ and T_tumor_ cells, T_eff_ showed a very distinct transcriptional profile (clusters 3, 5 and 6) which includes the upregulation of activation-associated genes such as *NR4A1/2/3* and *IFNG* (Fig. 1i, Supplementary Fig. 2b, c, Supplementary Data 1, 2). T_ex_ share some of these activation-associated transcriptional changes to a lower extent (cluster 2), which includes the upregulation of co-stimulatory receptors *TNFRSF9* (encoding 4-1BB), and the inhibitory receptors *LAG3 and CTLA4*. Along this line, T_ex_ showed pronounced upregulation of cluster 1, which contains *PDCD1* (PD-1), *TOX2*, and *PTPN6* (SHP-1) – transcripts that are associated with T cell exhaustion and PD-1 signaling^33,34^. T_rest_ displayed highest expression of clusters 4 and 6, which include the transcripts *TNF* and *IL7R*. T_tumor_ and T_ex_ showed higher expression of cluster 5, including the progenitor-associated transcripts *TCF7* and *SLAMF6*, and NK-associated transcripts *LILRB3*, *FCGR3A*, and *NKG7* (Fig. 1i, Supplementary Data 1, 2). Additionally, we used ISMARA to estimate the activities of NFAT/NR4A transcription factors, which are associated with exhaustion^33,35,36^. We observed higher motif activity for NFATc2/3 in T_eff_ and T_ex_ compared to T_rest_, and elevated activity of NR4A1 in T_ex_ compared to T_eff_ (Supplementary Fig. 2d).

Next, we performed gene set enrichment analyses for the comprehensive ‘PanCancer’ T cell gene signatures from Zheng et al. (Supplementary Data 3)^37^. T_tumor_ cells enriched mainly for the Temra signature, while both T_eff_ and T_ex_ showed high enrichment in the Tex-term signature (Fig. 1j). This is likely explained by the genes in the Tex-term signature, which are associated with activation and cell cycle such as *IL2RA* (CD25), *GZMB*, *TNFRSF9* and *CCND2* (Supplementary Fig. 2e, Supplementary Data 2). While T_eff_ express higher levels of these activation genes, other genes of the Tex-term signature, including *PDCD1*, *CD27*, *TOX*, *FUT8*, *LYST*, and *PDE7B*, were more expressed in the T_ex_ condition (Supplementary Fig. 2e, Supplementary Data 2). Only T_eff_ showed high enrichment in the proliferation-associated NME1-T signature. At the same time, T_ex_ enriched in the KIR+ TXK+ NK-like signature, which could suggest that they undergo an NK-like transition as recently described for exhausted CAR T cells^38^. Overall, our data show that T_ex_ resemble exhausted human TILs in terms of functionality and transcriptional changes.

### SNX9 is a potential mediator of T-cell exhaustion

**Fig. 2:**
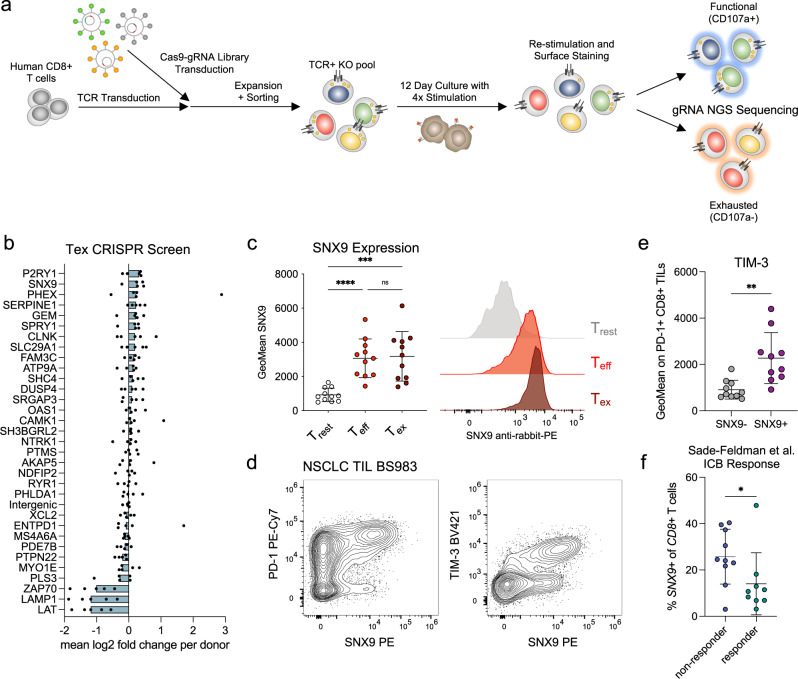
SNX9 is a potential mediator of T cell exhaustion. **a** Schematic description of the targeted-pooled CRISPR-Cas9 KO screen. **b** Mean log2 fold changes for gRNA enrichment in the pooled CRISPR/Cas9 screen. Dots represent the mean enrichment of all 5 guides for each donor biological replicate. **c** Representative flow cytometry plots and quantification of SNX9 expression measured by antibody staining in flow cytometry of human CD8 T cells of the ex vivo model with the different conditions. Mean and SD are shown. *n* = 11 donors of *n* = 3 experiments. Statistics are a 1-way ANOVA with Holm-Sidak correction. **d** Representative flow cytometry plots of SNX9 vs. PD-1 and TIM-3 expression in tumor-infiltrating CD8 T cells of a non-small cell lung cancer (NSCLC) patient. **e** TIM-3 expression measured by flow cytometry in SNX9 positive vs. SNX9 negative in intratumoral PD-1+ CD8 T cells of NSCLC patients. Statistics are a two-tailed paired t-test. Mean and SD are shown. n = 10. **f** Percentage of *SNX9*+ cells among CD8 T cells for each sample before treatment with ICI. Statistic is a Mann-Whitney test (non-normal distribution). We used 1 log normalized counts as the threshold for *SNX9* positivity. Samples with less than ten cells in either population were excluded, resulting in n = 10 nonresponders and n = 9 responders. **c**,** e**,** f** Mean and SD are shown. * *p* < 0.05, ** *p* < 0.01, *** *p* < 0.00 and **** *p* < 0.0001. Source data and exact p-values are provided as a Source Data file.

To discover genes regulating T cell exhaustion, we utilized the ex vivo exhaustion model to perform a targeted pooled CRISPR-Cas9 screen. Briefly, among genes with upregulated expression in T_ex_, we prioritized genes that were shared with published gene sets for human TILs and had little-known functions in T cell exhaustion (Supplementary Data 4). To perform the CRISPR-Cas9 screen, we simultaneously transduced primary human CD8 T cells with the NY-ESO-1 TCR construct and a pooled lentiviral library that encodes gRNAs and Cas9 (Fig. 2a). Sorted co-expressing cells were repetitively stimulated, stimulated again for 4 hours and then sorted by flow cytometry into cells with maintained degranulation potential (CD107a+, “functional”) and cells with impaired degranulation (CD107a-, “exhausted”). We then sequenced the gRNAs of each sample (*n* = 5 biological donor replicates) and used the PinAPL-py platform for gRNA mapping and counting^39^. For each donor replicate, we calculated the mean log2 fold change of the *n* = 5 guides per gene in both cell fractions. The median of these values for *n* = 5 donor replicates was then used to rank the genes (Fig. 2b). As expected, the three controls with known essential functions in T cell functionality (*LAT*, *LAMP1*, and *ZAP70*) showed the highest negative enrichment (*p* < 0.01, 1-way ANOVA). The highest-ranking genes for positive enrichment of gRNAs in the functional fraction of cells included *P2RY1*, *SNX9*, *SERPINE1*, and *PHEX* (Fig. 2b, ns, *p* > 0.05). None of these genes had been associated with changes in T cell proliferation in the CRISPR screen by Shifrut and colleagues (*P2RY1* KO had shown a trend towards lower proliferation, *p* = 0.057)^40^. Initial validation experiments were performed for these top-ranked genes using crRNA-tracrRNA-Cas9 electroporation of the guides with the highest log2 fold change. Unlike for *PHEX* and *P2RY1*, lower gene expression after electroporation was achieved for *SNX9* and *SERPINE1* (Supplementary Fig. 3a). Repetitive stimulation of individual KO T cells confirmed higher CD107a degranulation in *SNX9* KO T_ex_ (Supplementary Fig. 3b).

Based on the individual gene KO data, we decided to further investigate SNX9. First, we confirmed that T_eff_ and T_ex_ upregulated SNX9 on protein level both by flow cytometry and Western blot (Fig. 2c, d, Supplementary Fig. 3c–e). Furthermore, we found that SNX9 is significantly co-expressed with TIM-3 among PD-1+ TILs from NSCLC patients (Fig. 2d, e, Supplementary Fig. 3f, g). Mining a published single-cell ATACseq dataset of TILs from cancer patients, we found an open chromatin region (OCR) 7 kbp downstream of the transcriptional start site of the *SNX9* gene with increased accessibility in intermediate and late-exhausted compared to naïve, memory, effector, and early exhausted CD8 T cells (Supplementary Fig. 4a)^41^. This could suggest that epigenetic reprogramming associated with T cell exhaustion contributes to elevated SNX9 expression. Next, we re-analyzed published single-cell RNA sequencing data of melanoma TILs^17^. Within the CD8 tumor-infiltrating T cells cluster, *SNX9* expression was negatively correlated with the expression of central-memory and progenitor markers, *CCR7* and *TCF7* (Supplementary Fig. 4b, c). Consequently, *SNX9*+ T cells showed higher expression of inhibitory receptors *PDCD1* and *HAVCR2* (TIM-3), and exhaustion-related transcriptional regulators *TOX* and *TOX2*. As TCF7+ CD8 TILs correlate with response to ICB^17^, we investigated whether *SNX9* expression would be associated with therapy resistance. In this cohort of melanoma patients, the percentage of *SNX9*+ cells among CD8 T cells before treatment correlated with poor response to ICB (Fig. 2f). These findings further strengthened our hypothesis that SNX9 is involved in T cell exhaustion.

### *SNX9* KO improves effector functions of exhausted T cells and dampens the NFAT-NR4A1/3-TOX axis

**Fig. 3:**
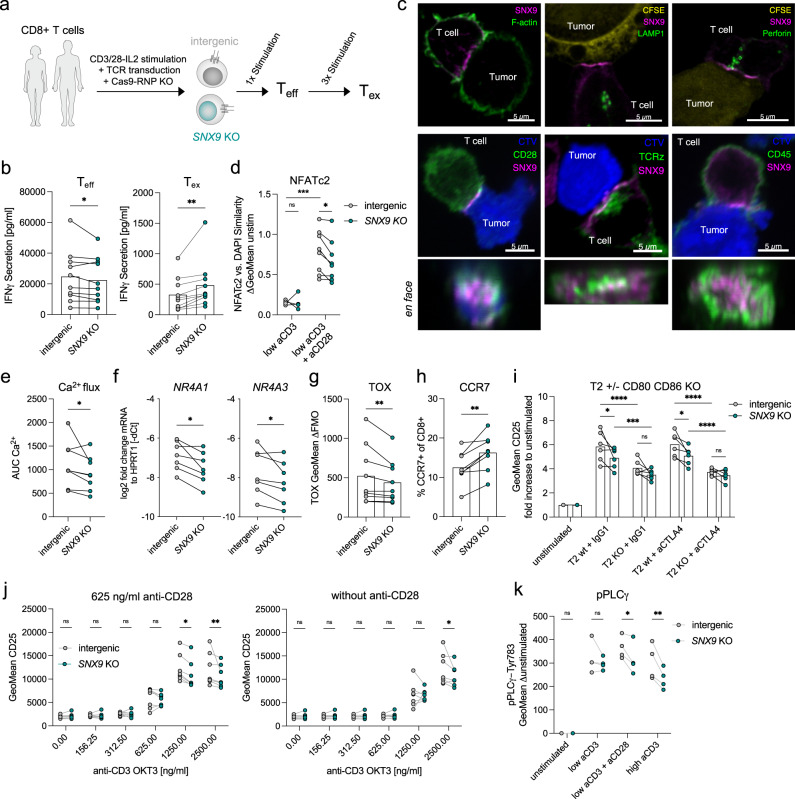
*SNX9* KO improves effector functions of exhausted T cells and dampens the NFAT-NR4A1/3-TOX axis. **a** Schematic procedure to generate SNX9 KO T cells. **b** IFNγ secretion by ELISA of T_eff_ and T_ex_ in response to re-stimulation. *n* = 10 donors (T_eff_) and 8 (T_ex_). **c** Top row: representative confocal images of T_ex_ co-cultured with T2 tumor cells for actin (*n* = 6), and LAMP1/Perforin (*n* = 3). Bottom row: example images of CD28-EGFP (*n* = 6) or TCRz-EGFP (*n* = 5) fusion proteins, or anti-CD45 (*n* = 6) and SNX9 in T_eff_. All images are provided in the Source Data file. **d** Nuclear translocation measured by image cytometry of NFATc2 in T_eff_ upon anti-CD3/28 stimulation. *n* = 7 donors of *n* = 2 experiments. **e** Area under the curve (AUC) of Calbryte520-AM Ca^2+^ flux upon stimulation. *n* = 8 donor of *n* = 3 experiments. **f** RT-qPCR quantifications of mRNA for NR4A1 and NR4A3 in cells on day 6 of the T_ex_ culture. *n* = 7 donors of *n* = 2 experiments. **g** Antibody staining (delta unstained) of TOX in T_ex_. *n* = 8 donors of *n* = 4 experiments. **h** Expression of CCR7 measured by flow cytometry antibody staining in T_ex_. *n* = 8 of n = 3 experiments. **b**, **e**-**h** Statistics are a two-sided paired t-tests. **i** Geometric mean CD25 upregulation relative to unstimulated cells for T_eff_ co-cultured with T2 wt or T2 CD80 CD86 KO (T2 KO) cells. 10 μg/ml human IgG1 or 10 μg/ml anti-CTLA4 blocking antibody (ipilimumab) was added. *n* = 6 donor replicates of *n* = 2 experiments. **j** CD25 geometric mean intensity of stimulated T cells with or without *SNX9* KO. *n* = 7 donors of *n* = 2 experiments. **k** Delta unstimulated geometric mean of phospho-PLCγ1-Tyr783 fluorescence intensity. *n* = 4 donors. **d**, **i**-**k** Statistics are paired 2-way ANOVA with Holm-Sidak correction. * *p* < 0.05, ** *p* < 0.01, *** *p* < 0.001 and **** *p* < 0.0001. Source data and exact p-values are provided as a Source Data file.

To mechanistically understand the contribution of SNX9 to the exhausted T cell state, we knocked out *SNX9* by Cas9-crRNA-tracrRNA electroporation and confirmed lower SNX9 protein levels by flow cytometry and Western blot (Fig. 3a, Supplementary Fig. 5a, b). Cell expansion was unchanged by *SNX9* KO, both for T_eff_ and T_ex_ (Supplementary Fig. 5c). As expected from our CRISPR-Cas9 screen and the initial validation, *SNX9* KO increased degranulation and IFNγ secretion in T_ex,_ whereas reduced IFNγ secretion was observed for *SNX9* KO T_eff_ (Fig. 3b, Supplementary Fig. 5d, e). In agreement with reports on the role of SNX9 in CD28 signaling in cell lines^42,43^, we observed clustering of SNX9 at active immune synapses in primary human CD8 T cells with a qualitative co-localization of SNX9 with the central supramolecular activation cluster (cSMAC) components TCRz (CD3ζ) and CD28 (Fig. 3c, Supplementary Fig. 5f). We observed only marginal co-localization with the distal SMAC (dSMAC) component CD45, or with LFA1 and lytic granules. These experiments indicate that SNX9 may be implicated in regulating T cell activation at the immune synapse.

Next, we asked whether *SNX9* KO impacts signaling through the Ca^2+^/NFAT pathways, as previously reported for cell lines^42,43^. Utilizing T_eff_ cells, we observed lower NFATc2 nuclear translocation *SNX9* KO cells upon anti-CD3+ anti-CD28 stimulation (Fig. 3d, Supplementary Fig. 5g). In agreement, we observed a decrease in the area under the curve for intracellular Ca^2+^ flux measured by Calbryte520-AM fluorescence upon anti-CD3 + anti-CD28 stimulation, whereas peak amplitudes were not significantly affected (Fig. 3e, Supplementary Fig. 5h). NFAT activation is important for T cell activation but has also been linked to the development of T cell exhaustion through downstream induction of NR4A1/2/3 and TOX/TOX2 expression^36,44,45^. We identified that the expression of *NR4A1*, *NR4A3*, and TOX was reduced in *SNX9* KO T_ex_ (Fig. 3f, g, Supplementary Fig. 5i). In addition to NFAT activation, CD28 activation is known to promote glycolysis and proliferation, which are, however, also linked to terminal differentiation^46^. Therefore, we further investigated how *SNX9* KO affects T cell metabolism and differentiation. We found that *SNX9* KO decreased the expression of lactate dehydrogenase A (*LDHA*), a critical enzyme in the glycolytic pathway (Supplementary Fig. 5i), and that *SNX9* KO T_ex_ had a lower glucose dependence with a switch towards fatty acid or amino acid oxidation (FAO/AAO), reminiscent of memory T cells (Supplementary Fig. 5j)^46,47^. Moreover, T_ex_ *SNX9* KO cells maintained elevated expression of the central-memory-associated receptor CCR7 (Fig. 3j, Supplementary Fig. 5k)^48^. Overall, our data suggests that *SNX9* KO decreases signaling through NFATc2-NR4A1/3-TOX and induces metabolic changes, which may both contribute to decreased exhaustion and increased central-memory-like differentiation.

We hypothesized that the observed reductions in Ca^2+^/NFAT signaling, glycolysis, and terminal differentiation in *SNX9* KO T cells might be explained by reduced CD28 signaling^42,43,49,50^. Therefore, we sought to investigate if *SNX9* specifically affects CD28 signaling in our experimental system with primary tumor-antigen-specific T cells. For this purpose, we generated T2 tumor cells with a double KO of both CD28 ligands CD80 and CD86 (termed “T2 KO”) (Supplementary Fig. 6a). We then stimulated both intergenic and *SNX9* KO T_eff_, either with NY-ESO-1 peptide-loaded T2 wildtype (“T2 wt”), or T2 KO cells and quantified CD25 upregulation as a marker of NFAT signaling^51^. As expected, stimulation with T2 KO resulted in lower CD25 upregulation compared to stimulation with T2 wt cells (Fig. 3i, Supplementary Fig. 6b, c). The KO of *SNX9* in T_eff_ resulted in lower upregulation of CD25 after stimulation with T2 wt cells, while no significant change was observed with T2 KO cells. Identical results were obtained when a blocking CTLA4 antibody was added to the co-culture to avoid CTLA-4 inhibitory signaling through CD80/86. No effect of *SNX9* KO was observed on CD25 expression in unstimulated T_eff_ (Supplementary Fig. 6b). *SNX9* KO T_eff_ cells showed decreased CD28 surface expression while expression of TCRβ was not affected (Supplementary Fig. 6d). In summary, we provide evidence that a loss of SNX9 in primary tumor-antigen specific T cells reduces activation in the context of intact CD28-CD80/86 signaling. In the absence of the latter, T cell activation was independent of SNX9 expression.

We sought to confirm these results in a reductionist antibody-based stimulation assay. To this end, we stimulated intergenic and *SNX9* KO T cells with plate-bound anti-CD3 antibody (OKT3 clone) alone or in combination with stimulatory plate-bound anti-CD28 antibody (CD28.2 clone). At an intermediate level of anti-CD3 (1250 ng/ml), we observed increased CD25 expression when combined with anti-CD28 co-stimulation (Fig. 3j). In this intermediate anti-CD3+ anti-CD28 stimulation condition, *SNX9* KO T cells showed reduced CD25 upregulation, which is compatible with our experiments above revealing CD28-dependent effects of SNX9. Unexpectedly, when increasing the level of anti-CD3 to a saturated range (2500 ng/ml), we observed reduced CD25 upregulation on *SNX9* KO T cells even in the absence of anti-CD28. In agreement, we also observed lower PLCγ1 phosphorylation (upstream initiator of calcium/NFAT signaling) with *SNX9* KO cells in response to intermediate anti-CD3+ anti-CD28 stimulation as well as high anti-CD3 stimulation alone (Fig. 3k). Contrastingly, we did not observe an effect of *SNX9* KO on the phosphorylation of AKT (downstream mediator of PI3K pathway) upon either stimulation (Supplementary Fig. 6e). In summary, our results suggest that SNX9 amplifies CD28-dependent upregulation of CD25 in the context of intermediate level TCR/CD3 signaling, while saturated TCR/CD3 stimulation provokes CD28-independent effects of SNX9 on CD25 and PLCγ1.

### *Snx9* KO improves anti-tumor efficacy and reduces terminal exhaustion in vivo

**Fig. 4:**
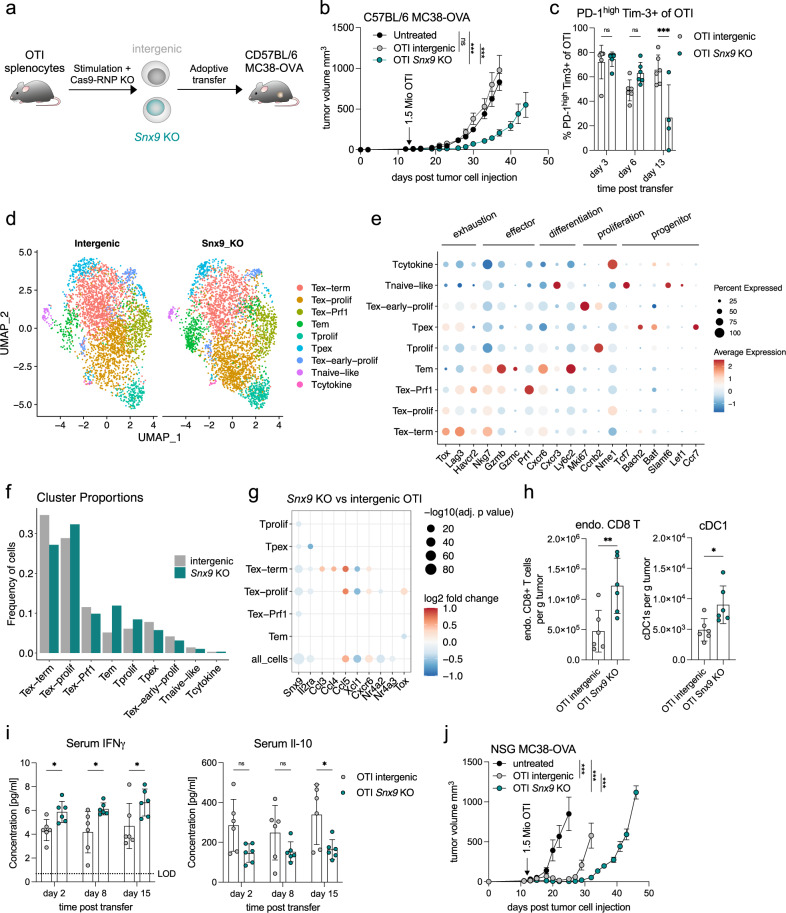
*Snx9* KO improves anti-tumor efficacy and reduces terminal exhaustion in vivo. **a** Schematic experimental setup of OTI *Snx9* KO generation and transfer to MC38-OVA-bearing mice. **b** Mean and SEM of MC38-OVA tumor volumes in C57BL/6 in *n* = 6 mice per condition. Curves are shown until the first mouse per group reaches the humane endpoint. Statistics are pairwise 2-way ANOVAs followed by Bonferroni correction. Representative of *n* = 3 experiments. **c** Percentage of PD-1^high^ Tim3+ OTI cells in MC38-OVA tumors in C57BL/6 mice on the indicated timepoints. *n* = 6 intergenic and *n* = 4 *Snx9* KO. Statistics are 2-way ANOVA with Holm-Sidak correction. **d** UMAP of single-cell RNA sequencing data from OTI cells isolated from MC38-OVA tumors 13 days post-transfer. Intergenic n = 3405 cells of 5 mice and *Snx9* KO *n* = 3612 cells of 6 mice. **e** Average expression of selected marker genes (color). Size = percentage of cells with detected expression for each cluster. **f** Proportions of each cluster in the intergenic versus the *Snx9* KO sample (*n* = 1 from 5–6 pooled mice per condition). **g** Selected differentially expressed genes between intergenic and OTI *Snx9* KO cells per cluster. Size indicates the -log10 adjusted p-value and color the mean log2 fold change. Statistics were derived by Seurat’s FindMarkers function. **h** Quantification of endogenous CD8 T cells (left) and cDC1s (right) in MC38-OVA tumors 3 days post transfer of OTI cells. Statistics are unpaired t-tests. *n* = 6 mice per group (**i**) Serum levels of IFNγ and Il-10 for days 2,8 and 15. Limit of detection (LOD) is indicated for IFNγ. Statistics are unpaired 2-way ANOVA with Holm-Sidak correction. *n* = 6 mice per group. **j** Tumor volume (mean and SEM) of NSG mice with subcutaneous MC38-OVA tumors with a transfer of OTI cells at day 12 postinjection. *n* = 6 mice per OTI condition, *n* = 4 for untreated. Statistics are pairwise 2-way ANOVAs followed by Bonferroni correction * *p* < 0.05, ** *p* < 0.01, *** *p* < 0.001. **c**, **h**-**i** Mean and SD are shown. Source data and exact p-values are provided as a Source Data file.

We then asked whether the reduced initial activation coupled to a later reduction in exhaustion observed with an *SNX9* KO in the ex vivo model would also translate into improved in vivo efficacy. To this aim, we knocked out *Snx9* in pre-stimulated OTI splenocytes (murine OVA-specific CD8 T cells) by Cas9-crRNA-tracrRNA electroporation, which significantly reduced Snx9 protein expression (Fig. 4a, Supplementary Fig. 7a, b). The adoptive transfer of 1.5 Mio *Snx9* KO OT cells to MC38-OVA tumor-bearing mice reduced tumor growth and improved survival (Fig. 4b, Supplementary Fig. 7c, d). No therapeutic effects of *Snx9* KO were observed with P14 T cells, which recognize the LCMV gp33 peptide (Supplementary Fig. 7e). These results demonstrate that *Snx9* KO improves antigen-specific anti-tumor efficacy of adoptively transferred OTI T cells.

We wanted to better understand how *Snx9* KO improves anti-tumor efficacy in vivo, and, therefore, characterized OTI number and phenotype in MC38-OVA tumor-bearing mice. While the frequency of OTI cells in the tumor was unchanged (Supplementary Fig. 8a, b), *Snx9* KO OTI cells at day 13 post transfer co-expressed less PD-1 and Tim-3, which could indicate a less exhausted T cell state (Fig. 4c, Supplementary Fig. 8c–e)^51^. To further investigate this, we performed single-cell RNA sequencing analyses of intratumoral OTI cells 13 days postadoptive transfer. Our analysis pipeline in Seurat resulted in seven clusters ranging from naïve-like cells to terminally exhausted populations, which we termed based on differentially expressed genes, transcription factor expression, and gene set enrichment for published cell populations (Fig. 4d, e, Supplementary Fig. 9a–c). *Snx9* KO OTI cells were less frequently found in the Tex-term cluster (terminally exhausted, high in *Tox* and *Lag3*), while they were enriched in Tem (effector-memory-like, high in *Gzmb*, *Gzmc*, *Cxcr6*, and *Ly6c2*) and the Tex-prolif clusters (proliferating exhausted T cells, high in *Nme1*).

We next investigated the differentially expressed genes between *Snx9* KO and intergenic OTI cells within each cluster and among all cells. First, we observed that *Snx9* itself and *Il2ra* (encoding CD25) were downregulated among multiple clusters, confirming our ex vivo findings with human T cells (Fig. 4g, Supplementary Fig. 9d, Supplementary Data 5). Further, we found that *Snx9* KO OTI cells expressed higher levels of the chemokines *Ccl3*, *Ccl4*, and *Ccl5* in the Tex-term cluster (and for *Ccl5* also the Tex-prolif cluster), while *Xcl1* was reduced. In two Tex clusters, we found higher expression of *Cxcr6*, which is associated with effector functions and tumor infiltration^52^. Unlike in the Tex-prolif cluster, we observed reduced *Tox* expression in *Snx9* KO OTI cells within the effector-memory-like Tem cluster. Additionally, *Nr4a2* was reduced in the Tex-prolif cluster and among all cells, while *Nr4a3* was changed only considering all cells. *Nr4a1* and *Ifng* were not significantly changed, and *Il10* was not detected (Supplementary Data 5). These results suggest that *Snx9* KO OTI T cells express less *IL2RA* and *Nr4a2/3* while they upregulate several genes associated with effector-memory differentiation and anti-tumor immunity.

Ccl3, Ccl4, and Ccl5 are well-known chemokines that attract other immune cells into tumors including dendritic cells, monocytes, and T cells^53^. Therefore, we investigated the number of endogenous immune cells within MC38-OVA tumors 3 days post-OTI transfer. In agreement with a higher chemokine expression, we observed more endogenous CD8 T cells and cDC1s (CD11c+ MHCII+ F4/80− CD103+) in tumors after transfer of *Snx9* KO OTI cells (Fig. 4h, Supplementary Figs. 8e, 9e). These changes in the intratumoral immune composition were accompanied with higher serum levels of IFNγ and lower levels of the immunosuppressive cytokine Il-10 (Fig. 4i). We detected no change in Ccl5, Cxcl10, and Il-6 in the serum (Supplementary Fig. 9f). Notably, we observed a similar improvement in anti-tumor efficacy by *Snx9* KO when OTI cells were transferred to (immunodeficient) NSG mice with MC38-OVA tumors (Fig. 4j, Supplementary Fig. 9g). This suggests that while *Snx9* KO OTI cells promote the recruitment of other immune cells, *Snx9* KO OTI cells can directly mediate anti-tumor effects in NSG mice independent of an intact endogenous immune system.

### Deletion of *SNX9* improves CAR T cell anti-tumor efficacy

**Fig. 5:**
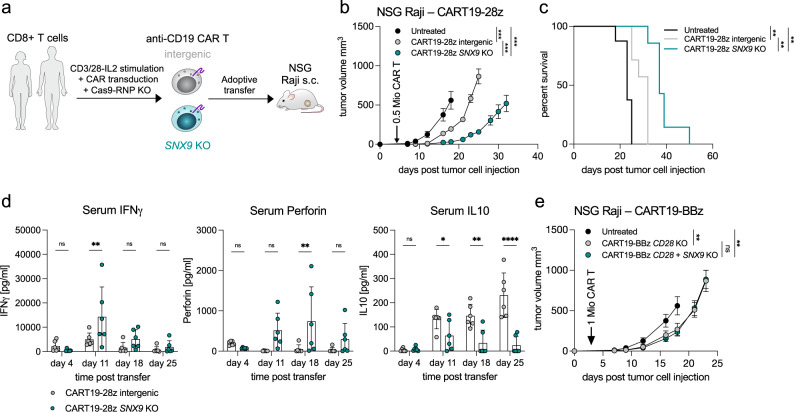
Deletion of *SNX9* improves CAR T cell anti-tumor efficacy. **a** Schematic representation of the CAR T cell transfer experiments. Healthy donor human CD8 T cells are stimulated ex vivo and lentivirally transduced with an anti-human-CD19(FMC63vH)-CD28-CD3zeta-T2A-copGFP CAR construct and electroporated with Cas9-crRNA-tracrRNA complexes to generate *SNX9 KO* cells and intergenic controls. These cells are then transferred to NSG mice with subcutaneous Raji tumors (CD19+). **b** Tumor volume in mm^3^ of NSG mice treated 3 days post Raji tumor injection by i.v. transfer of 0.5 Mio human CD8 anti-CD19-28z CAR T cells with or without *SNX9* KO (mean and SEM). Statistics are pairwise 2-way ANOVAs followed by Bonferroni correction. *n* = 8 animals for untreated of *n* = 2 experiments. *n* = 7 mice for CART-treated mice of *n* = 1 experiment. Experiment was replicated with similar results with higher CART numbers. **c** Survival of the NSG mice in 5b until humane endpoint of 1500mm^3^ tumor size. Statistics are log-rank Mantel-Cox tests followed by Bonferroni correction. (**b** and **c**): *n* = 8 for untreated, *n* = 7 for intergenic and *SNX9* KO CAR T conditions. **d** Human cytokines measured by Legendplex (Biolegend) in the serum of Raji-bearing NSG mice treated with anti-CD19-28z CAR T cells with and without *SNX9* KO. Statistics are paired-2-way ANOVA with Holm-Sidak correction. *n* = 6 mice per condition. Mean and SD are shown. **e** Tumor volume in mm^3^ (mean and SEM) of NSG mice treated 3 days post Raji tumor injection by i.v. transfer of 1 Mio human CD8+ CD28 KO anti-CD19-BBz CAR T cells with or without *SNX9* KO. *n* = 6 for intergenic and *SNX9* KO, *n* = 8 for untreated. Statistics are 2-way ANOVAs followed by Bonferroni correction. * *p* < 0.05, ** *p* < 0.01, *** *p* < 0.001. Source data and exact p-values are provided as a Source Data file.

As we observed improved efficacy of *Snx9* KO in murine adoptive transfer models, we investigated whether *SNX9* KO would also improve the efficacy of human CAR T cells in xenograft models. To test this, we generated human anti-CD19 CAR T cells harboring a CD28-CD3ζ co-stimulation domain with or without *SNX9* KO and transferred them to NSG mice with subcutaneous human CD19+ Raji tumors (CART19-28z, Fig. 5a). Compared to the intergenic control, CART19-28z *SNX9* KO cells improved long-term anti-tumor control and survival (Fig. 5b, c). Injecting three times the number of CARs did not improve the survival for CART19-28z intergenic, while CART19-28z *SNX9* KO again led to higher survival (Supplementary Fig. 10a, b). Notably, the transfer of CART19-28z *SNX9* KO was accompanied with increased serum levels of anti-tumor effector molecules IFNγ, Perforin, and Granulysin, while we detected a decrease in IL10 and IL6 (Fig. 5d, Supplementary Fig. 10c, human CCL5 could not be detected in the serum). To investigate the dependency on CD28 signaling in vivo, we used a 4-1BB domain-containing anti-CD19 CAR and additionally knocked out the endogenous CD28 (“CART19-BBz CD28 KO”, Supplementary Fig. 10d). The transfer of CART19-BBz CD28 KO intergenic induced delays in Raji tumor growth comparable to CART19-28z intergenic (Fig. 5b, e), while we did not observe an additional effect of *SNX9* KO with CART19-BBz CD28 KO cells. Overall, this suggests that the effects of *SNX9* KO in CAR T cells in vivo depend on the presence of CD28 signaling.

## Discussion

We here developed a human ex vivo model for antigen-specific T cell exhaustion using primary CD8 T cells. This allowed us to perform a targeted CRISPR-Cas9 screen to rank gene knockouts according to their enrichment in functional T cells. Investigating the highest-ranking genes, we discovered that *SNX9* KO reduces T-cell signaling via PLCγ1-NFAT-NR4A1/3 and dampens T-cell exhaustion. The in vivo anti-tumor efficacy of adoptively transferred murine TCR transgenic T cells was improved by *Snx9* KO, which correlated with increased effector-memory-like differentiation, enhanced chemokine expression, and elevated IFNγ serum levels. Similarly, *SNX9* KO improved human CART19-28z efficacy in vivo which was accompanied by elevated IFNγ, perforin, and granulysin levels in the serum, whereas IL10 and IL6 were reduced. Moreover, *SNX9* expression in CD8 T cells correlated with resistance to immunotherapy in melanoma patients (graphically summarized in Supplementary Fig. 11).

The ex vivo model developed here enables the generation of millions of cancer-associated exhausted T cells from peripheral human blood. The cells generated with this approach acquire features of T cells in human tumors, such as co-expression of inhibitory receptors, reduced effector functions, and impaired proliferation^2^, a phenotype recently also observed with continuously stimulated CAR T cells^38^. The simplicity and versatile nature of the current ex vivo exhaustion model enables immediate testing of novel compounds or drug targets in a fully human system. Prospectively, the model can be refined to investigate mechanisms beyond persistent antigenic stimulation by including metabolic restriction, low oxygen availability, suppressive cytokines, or immunosuppressive cells which would allow the replication of important aspects of the tumor microenvironment^54^.

Pooled CRISPR-Cas9 screens have been previously used in cancer cells to discover important genes for immunotherapy resistance^55,56^. Additionally, in vivo CRISPR-Cas9 KO screens in murine T cells have been published to improve anti-tumor efficacy^20,21^. However, due to low T cell infiltration in the tumor, in vivo screens often suffer from guide underrepresentation limiting the discovery of new targets^21^. While none of the highest-ranking genes of our CRISPR-Cas9 screen were statistically significant due to experimental variation, the screen allowed us to prioritize genes for single gene validation experiments and thereby discover SNX9’s role in T cell exhaustion. Notably, several studies performed genome-wide CRISPR screens in human T cells using a Cas9 electroporation-based protocol that was shown to enhance gRNA vector transduction rates^40,57,58^. Their protocols provide a perspective to reduce the observed variability in our screen by increasing the number of possible guides per gene, cells per guide, and donor replicates, as well as to perform larger screens utilizing our human exhaustion model. Recently, genome-wide CRISPR/Cas9 screens using polyclonal stimulation have been used to discover potential regulators of T cell exhaustion^23,59^. *SNX9* was not discovered in these screens, and we speculate that reasons for this include the lack of T cell – tumor cell interactions and their proliferation readouts. Another exciting possibility for future studies utilizing our model system might be the use of gain-of-function CRISPR screens, as recently reported for murine CAR T cells^60^.

Our targeted CRISPR-Cas9 screen and the subsequent validation experiments revealed SNX9 as a potential mediator of T-cell exhaustion, which we confirmed ex vivo and in vivo. While *SNX9* has been identified in expression analyses of tumor-infiltrating T cells before^16,18,61,62^, its role in T cell exhaustion has remained unexplored. SNX9 was reported to enhance CD28 signaling, which is generally considered to be beneficial for CD8 T cells^42,63,64^. Badour and colleagues described that SNX9 amplifies CD28 signaling by increasing its internalization. They described interactions of SNX9 with the PI3K subunit p85 and PI(3,4,5)P_3_ via its PX domain^42,63^, while a more recent human CD28 interactome lists a direct interaction of SNX9 with the cytosolic part of CD28^65^. In agreement with its known functions linking different cytoskeletal proteins and membrane lipids^43,66^, Ecker et al. showed that SNX9 contributes to positioning and stabilization of CD28 clusters at the immune synapse in the CD4 Jurkat T cell line^43^.

Our experiments utilizing a human antigen-specific model system (Fig. 3i) and the adoptive transfer of CAR T cells (Fig. 5a–c) indicate that the effects of *SNX9* KO are largely CD28-dependent. However, with saturated levels of TCR/CD3 stimulation ex vivo - which are likely not achieved under physiological conditions - *SNX9* KO appears to decrease T cell activation even in the absence of CD28 co-stimulation. Accordingly, Ecker et al. observed lower but significant recruitment of SNX9 to the immune synapse with low-dose anti-CD3 stimulation alone, however, this was more pronounced with anti-CD3+ anti-CD28 stimulation^43^. Intriguingly, we did not find evidence that SNX9 modulates PI3K/AKT signaling directly, in contrasts to its effects on PLCγ1/Ca^2+^/NFAT signaling. SNX9 is known to bind PI(4,5)P_2,_ PI(3,4,5)P_3_, N-WASP, and trigger Arp2/3-dependent polymerization of actin filaments^42,67^. This raises the interesting possibility that SNX9 functions downstream of TCR/CD28-evoked PI3K activity by enhancing the assembly of ITK, TEK, and WASP-dependent actin regulatory processes, which are known to promote PLCγ1 signaling^42,49^. More research is required to elucidate in detail how SNX9 promotes TCR/CD28 downstream signaling.

We discovered that while *SNX9* KO reduces T cell activation, it also enhances anti-tumor immunity. Most current approaches to alleviate T cell exhaustion aim to increase co-stimulation or decrease co-inhibition. For example, PD-1 blockade is thought to primarily rescue impaired CD28 co-signaling^68^ and CD28 is required for the efficacy of anti-PD1 antibodies in vivo^69^. On the other hand, there is mounting evidence that lack of co-inhibition during antigenic stimulation can lead to T cell exhaustion. For example, the genetic absence of PD-1 increases T cell exhaustion in mice, presumably due to overactivation^70^. In agreement, several methods to dampen T cell overactivation have been shown to reduce T cell exhaustion, including transient pharmacological resting or inhibition of calcium signaling in CAR T cells, or *Cd8a* KO in OTI cells^71–73^. Similarly, small changes in the CAR CD3ζ or CD28 signaling domains reducing activation are known to promote memory differentiation and tumor rejection^74,75^. These findings imply that an optimal balance of co-stimulation is necessary to achieve and maintain functional, tumor-specific effector T cells. *SNX9* KO appears to induce a small but significant decrease in NFAT signaling and is therefore an attractive way to fine-tune T cell activation.

Mechanistically, our ex vivo results show that *SNX9* KO T_ex_ have a lower expression of *NR4A1*, *NR4A3*, and TOX, which have been implicated in T cell exhaustion^36,44,45^. In vivo, we also found that *Snx9* KO OTI cells express lower levels of *Nr4a2 and Nr4a3*, while effects on Tox expression were less consistent. We discovered that *Snx9* KO OTI cells expressed more effector-memory-associated proteins such as *Cxcr6*, *Ccl5*, *Ccl4*, and *Ccl3* and induced higher recruitment of CD8 T cells and cDC1s. Cxcr6 was recently described to promote the maintenance of cytotoxic CD8 T cells in tumors through interactions with Ccr7+ dendritic cells^52^, a potential factor how *Snx9* KO OTI cells may enhance the endogenous anti-tumor immune response. Along this line, adoptive transfer of CART19-28z *SNX9* KO provoked higher serum levels of cytotoxic granule proteins and IFNγ, while the immunosuppressive cytokine IL10 was markedly reduced^76,77^. IL10 secretion is known to correlate with TCR signaling strength, inhibit CD28-signaling, and increase the activation threshold of T cells in a negative-feedback loop, which might be dampened by *SNX9* KO^77–79^. In summary, *SNX9* KO alleviates exhaustion, increases chemoattraction, and prolongs IFNγ secretion of tumor-specific T cells.

Despite its upregulation in exhausted TILs, SNX9 is rather ubiquitously expressed in humans^66,80^. Thus, a knockout of *SNX9* ex vivo*, e.g*. in TILs and genetically engineered T cell products appears to be an ideal strategy for clinical use. As a proof-of-concept, we showed that *Snx9* KO improved the antitumor efficacy of adoptively transferred TCR-transgenic T cells and human CAR T cells. The inclusion of costimulatory domains, e.g. derived from CD28 or 4-1BB, is required for optimal CAR T cell persistence, cytokine production, and tumor rejection in vivo^81^. There is evidence that addition of a CD28 domain drives effector memory differentiation with metabolic reprogramming towards aerobic glycolysis^12,50^. This is in line with our findings for *SNX9* KO T cells, which exhibited less CD28/TCR signaling, lower glucose dependence and increased expression of CCR7. Several studies have demonstrated that CAR T cells with a CD28 costimulatory domain release higher quantities of cytokines that may result in adverse effects^82,83^. Notably, in the NSG mice treated with *SNX9* KO CART19-28z, we found lower serum levels of IL6, one of the main cytokines involved in CAR T-mediated cytokine release syndrome^84^. Therefore, it is intriguing to speculate that lowering TCR/CD28 signaling, e.g., by *SNX9* KO, may also reduce the toxicities associated with CAR T cells.

In summary, our findings suggest that SNX9 fine-tunes T cell activation and is a potential target to improve the efficacy of cellular immunotherapies in cancer patients.

## Methods

### Ethics declaration

All research conducted for this study complies with all relevant ethical and safety regulations. All procedures performed in studies involving human participants were in accordance with the ethical standards of the institutional and/or national research committee (Ethikkommission Nordwestschweiz, EK321/10) and with the 1964 Helsinki declaration and its later amendments or comparable ethical standards. Informed consent was obtained from all individual participants included in the study. All animal experiments were performed in accordance with Swiss federal regulations and licenses (numbers 2408_34213, 2370_34209) were approved by the cantonal veterinary office (animal experimentation committee, Tierversuchskomission) of Basel-Stadt (CH). Handling of potentially hazardous materials was conducted according to local safety standards and approved by the federal authorities (BAFU CH, approval number A172087).

### Nomenclature

According to the HUGO convention, human genes and transcripts are written in all capital letters and italic (e.g. *SNX9*). Human proteins are indicated in all capital letters (e.g. SNX9). Murine genes and transcripts are written in italic with the first letter in capital letters (e.g. *Snx9*). Murine proteins are written with the first letter in capital letters (e.g. Snx9). Official gene symbols and protein names are used when applicable according to the Uniprot Consortium (uniprot.org). CD nomenclature is used for CD107a (official protein name LAMP1).

### Peptides

Peptides were purchased in >95% purity from EZ Biolabs. Lyophilized peptides were resuspended at 10 mM in sterile dimethyl sulfoxide (DMSO) and stored at −20 °C until use. The endogenous NY-ESO-1 peptide SLLMWIQC was shown to elicit half-maximal response by LAU155 TCR transduced T cells at an EC50 of 12 nM^85^. The NY-ESO-9V peptide used in this manuscript has a 4500-fold relative competitor activity towards HLA-A2010 and 200-fold higher antigenic activity (half-maximal dose required for killing of T2 tumor cells) compared to the NY-ESO-9C endogenous peptide^85^.

### Cell culture media

For the culture of T2, Jurkat and NA8-Mel cells, RPMI1640 (Sigma) was supplemented with 10% heat-inactivated (56 °C 30 min) fetal calf serum (PAN Biotech), 100 ng/ml penicillin/streptomycin (Sigma), 2 mM L-Glutamine (Sigma), 1 mM Sodium Pyruvate (Sigma),1% MEM non-essential amino acids (Sigma), 50 nmol/l beta-mercaptoethanol (Thermo Fisher) and 10 mM HEPES (Sigma). In later experiments T2, Jurkats, Raji and NA8-Mel cells were cultured in the media described above but replacing the 10% FCS with 2% FCS and 10% of Panexin Basic FCS Replacement (PAN Biotech) which results in identical growth kinetics.

For the culture of human T cells, RPMI1640 was supplemented as described above, but FCS was replaced with 8% heat-inactivated AB+ male donor serum and 50 μM normocin (Invivogen) was added. Recombinant human IL-2 (Peprotech or Proleukin) was always added freshly at the indicated doses.

For the culture of HEK293T cells, DMEM (Sigma) was supplemented with 5% heat-inactivated (56 °C 30 min) fetal bovine serum (FBS, PAN Biotech), 100 ng/ml penicillin/streptomycin (Sigma), 2 mM L-Glutamine (Sigma), 1 mM Sodium Pyruvate (Sigma) and 1% MEM non-essential amino acids (Sigma) and 10 mM HEPES (Sigma).

### Cell Lines

T2 cells (ACC598, RRID:CVCL_2211) and Jurkat (ACC282, RRID:CVCL_0065) were purchased from DSMZ, Leibnitz Institute. HEK293T cells (ATCC CRL-3216, RRID:CVCL_0063) and Raji (ATCC CCL-86, RRID:CVCL_0511) were purchased from ATCC. The melanoma cell line NA8-Mel (RRID:CVCL_S599) generated by Dr. F. Jotereau (U211, Institut National de la Santé et de la Recherche Médicale, Nantes, France) was kindly provided by Dr. Romero (University of Lausanne) and cultured in RPMI-1640 supplemented as described above. T2, Jurkat, Raji and NA8-Mel cells were cultured in supplemented RPMI as described in Section Cell Culture Media above. Murine MC38-OVA colon cancer cells (provided by Mark Smyth, Peter MacCallum Cancer Centre, Melbourne, Australia) were cultured in Dulbecco’s modified Eagle’s medium (DMEM, Gibco) supplemented with 10% heat-inactivated FBS (Gibco), sodium pyruvate (Sigma), penicillin/streptomycin (Gibco), and minimal essential medium nonessential amino acids (Sigma). All cells were confirmed to be negative for mycoplasma by PCR as described^86^ after every freeze-thaw cycle and then passaged every 2–3 days for a maximum of 15 passages. For adherent cell lines, TrypLE Express (recombinant Trypsin replacement, Thermo Fisher) or 0.05% Trypsin-EDTA (Thermo Fischer) was used.

### Mice

Wildtype (CD57BL/6NRj), OT-I (C57BL/6-Tg(TcraTcrb)1100Mjb/J, RRID:IMSR_JAX:003831), and NSG (NOD.Cg-Prkdc<scid>Il2rg < tm1Wjl>SzJ, RRID:IMSR_JAX:005557) mice were bred in-house at the University Hospital Basel, Switzerland. Animals were housed under specific pathogen-free conditions. For all experiments female mice were used. Sex-matched littermates at 8–12 weeks of age at the start of the experiments were used. Maximally allowed tumor burden of 1500mm^3^ was not exceeded. All animal experiments were performed in accordance with Swiss federal regulations and licenses (numbers 2408_34213, 2370_34209) were approved by the cantonal veterinary office of Basel-Stadt (CH). Mice were maintained in a sterile controlled environment (a gradual light–dark cycle with light from 7:00 to 17:00, 21–25 °C, 45–65% humidity).

### Tumor models

C57BL/6NRj and NSG mice were injected subcutaneously into the right flank with 1 Mio (CD57BL/6NRj) or 0.25 Mio (NSG) syngeneic murine MC38-OVA colon cancer cells suspended in phenol red-free DMEM (without additives) or 0.5 Mio human Raji lymphoma cells suspended in Corning® Matrigel® Matrix High Concentration Phenol-Red-Free diluted 1:1 in phenol red-free DMEM without additives. Cell lines were tested for mycoplasma contamination before injection by PCR as above. Tumor volume was calculated according to the formula: D/2 x d x d, with D and d being the longest and shortest tumor diameter in mm, respectively.

### Cellular tumor assessment

Tumor tissue was isolated from mice, weighed, and minced using razor blades. Tissue was then digested using accutase (PAA), collagenase IV (Worthington), hyaluronidase (Sigma), and DNAse type IV (Sigma) for 60 min at 37 °C with constant shaking. Cell suspensions were filtered using a cell strainer (70 μm). Precision Counting beads (Biolegend) were added before staining to quantify the number of cells per gram tumor. Single-cell suspensions were blocked with rat anti-mouse FcγIII/II receptor (CD16/CD32) blocking antibodies (“Fc-Block”) and stained with live/dead cell-exclusion dye (Zombie UV dye; Biolegend) for 20 min at 4 °C then washed with FACS buffer (PBS supplemented with 2 mM EDTA, 0.1% Na-Azide, 2% FCS) by centrifugation at 500 g for 3 min. Cells were then incubated with fluorophore-conjugated antibodies directed against cell surface antigens in Brilliant Stain buffer (BD) for 20 min at 4 °C, washed, and fixed and permeabilized using Foxp3/transcription factor staining buffer set (eBioscience) prior to incubation with antibodies directed against intracellular antigens in FACS buffer. Cell populations were analyzed on a Cytek Aurora. The following gating strategies were used: OTI T cells: live singlet CD19- Ly6G- CD45.2- CD45.1+ CD8+; Endogenous T cells: live singlet CD19- Ly6G- CD45.2+ CD45.1- F4/80- CD11c- CD8+ or CD4+; NK cells: live singlet CD19- Ly6G- CD45.2+ CD45.1- F4/80- CD11c- CD8- CD4- CD3- MHCII- NKp46+; cDC1: live singlet CD19- Ly6G- CD45.2+ CD45.1- CD11c+ F4/80- MHCII+ CD3- Ly6C- CD103+ CD11b-; cDC2: live singlet CD19- Ly6G- CD45.2+ CD45.1- CD11c+ F4/80- MHCII+ CD3- Ly6C- CD103- CD11b+; B cells: live singlet CD19+; Neutrophils: cDC1: live singlet CD19- Ly6G+ CD11b+; Macrophages: live singlet CD19- Ly6G- CD45.2+ CD45.1- CD11b+ F4/80+ Ly6C_low_; M2 macrophages: live singlet CD19- Ly6G- CD45.2+ CD45.1- CD11b+ F4/80+ Ly6C_low_ CD206+; Monocytes: live singlet CD19- Ly6G- CD45.2+ CD45.1- CD11b+ F4/80_low_ Ly6C_high_.

### Isolation of primary immune cells

Human peripheral blood mononuclear cells (PBMCs) were isolated by density gradient centrifugation using Histopaque-1077 (Sigma) from buffy coats obtained from healthy blood donors (HD) (Blood Bank, University Hospital Basel). Briefly, buffy coat was diluted in PBS and layered on top of Histopaque-1077 and spun in SepMate (Stem Cell) according to manufacturer’s instructions. Red blood cells were lysed using Red Blood Cell Lysis Kit (eBiosciences), washed and frozen in 10% DMSO 90% FCS in a Styrofoam container to −80 °C. For long term storage (>1 week), cells were transferred to liquid nitrogen. Blood from a total of 47 different healthy human donors was used, of which 30 were male (63,83%). Median age was 47.5 years (mean = 45.1, min = 20, max = 74) Healthy donors were self-recruited potentially creating a bias for males.

Fresh tumor tissues were collected from patients with NSCLC undergoing surgery at the University Hospital Basel, Switzerland. The study was approved by the local ethical review board (Ethikkommission Nordwestschweiz, EK321/10), and all patients consented in writing to the analysis of their tumor samples. Tumor lesions were mechanically dissociated and digested using accutase (PAA), collagenase IV (Worthington), type V hyaluronidase from bovine testes (Sigma), and DNAse type IV (Sigma), directly after excision. Single-cell suspensions were prepared and frozen as above. For the SNX9 staining in NSCLC patient TILs, the median age at resection was 70.6 years, average 69.9 years (min-max: 54.6–83 years). 6 patients were male and 5 female. Tumor collection was conducted within the framework of a surgical procedure as decided and approved by an interdisciplinary tumor board. Confirmation of malignancy was obtained from a board-certified pathologist.

### T cell receptor construct

The lentiviral construct encoding for the codon-optimized pRRL 131 (WT) T2A 1xATG Cys LAU155 NY-ESO-1 T cell receptor consists of alpha and beta chains under an hPGK promotor separated by a T2A sequence and was kindly provided by Dr. Michael Hebeisen and Dr. Natalie Rufer at the University of Lausanne^29,87^. This TCR has a K_D_ = 21.4 μM for its endogenous NY-ESO-1 SLLMWITQC peptide.

### Generation of lentivirus

To generate lentivirus, 2.5 million low passage HEK293T cells were cultured in DMEM medium and seeded into a 15 cm tissue-culture treated dish. Three days later, 2nd generation LTR-containing donor plasmid, packaging plasmid pCMV-delta8.9 and the envelope plasmid VSV-G were mixed at a ratio of 4:2:1 ratio in unsupplemented Opti-MEM (ThermoFisher) and sterile filtered. This solution was then mixed with polyethyleneimine 25 kDa (Polysciences Inc.), also diluted in Opti-MEM at a DNA:PEI ratio of 1:3. 28 μg of DNA was transfected per 15 cm dish. After two days, supernatants were collected from cells (exchange medium on plates) and filtered through a 0.45 μm PES filter. Supernatants were stored for 1 day at 4 °C until the second batch of supernatant was collected 24 h later. The supernatant containing lentiviral particles was concentrated by ultra-centrifugation at 40’000 x  *g* for 2 h at 4 °C, resuspended in 0.1% BSA in PBS, and frozen to −80 °C. To ease production of antigen specific T cells, virus production for the NY-ESO-1 TCR vector was later outsourced to Vectorbuilder Inc (USA), who provided stocks of > 1*10^9 TU/ml lentivirus produced by PEG precipitation (measured by p24 ELISA).

### Transduction of human T cells

To generate NY-ESO-1 TCR-specific T cells, human healthy donor PBMCs were thawed and washed in PBS. CD8 T cells were then isolated using the CD8 microbeads kit (Miltenyi, positive selection) according to the manufacturer’s instructions on an AutoMACS. Isolated cells were washed and resuspended in suppl. RPMI with 8% human serum as described above and plated at 1.5 Mio/ml. T cell activation and expansion kit (Miltenyi) anti-CD3+ anti-CD28 stimulatory magnetic beads were coated overnight at 4 °C on a rotator shaker according to the manufacturer’s instructions. These beads were washed in medium and added to the CD8 T cells at a 1:1 ratio together with 150 U/ml IL-2. 24 h later, NY-ESO-1 TCR lentiviral particles produced as described above were added at a multiplicity of infection (MOI) of 2. Cells were then expanded every 2 days with fresh medium and replenishing 50 U/ml IL-2 for 5 days. The percentage of transduced cells was then calculated by staining for TCR Vbeta13.1+ T cells in comparison to non-transduced cells. TCR Vbeta 13.1+ cells were sorted by staining of TCR-Vbeta13.1 antibody by flow cytometry (H131 clone PE-Cy7, 1:25, SorpAria) or using purified antibody (H131 clone purified, 1:50 dilution) followed by magnetic column purification (anti-mouse IgG Microbeads, Milentyi).

### Repetitive stimulation

NY-ESO-1 TCR specific T cells were plated at 0.125 mio/ml specific cells. T2 tumor cells were irradiated with 3000 rad gamma rays using a GammaCell irradiator. Irradiated cells were mixed with the indicated dose (1 μM) of NY-ESO-1 9 V peptide and added to the T cells at an effector-to-target ratio of 1:3 in the presence of 50 U/ml IL-2. This procedure was repeated (with IL-2 stimulation at each stimulation) according to the scheme in Supplementary Fig. 1a. At each stimulation, 75% of the medium was replaced. The cells were expanded 1:2 on the day of the second stimulation with tumor cells and peptide. T_tumor_ were treated identically, but without the addition of NY-ESO-1 9 V peptide. For T_rest_, we only exchanged 75% of the medium every three days replenishing IL2. Acute stimulation controls were only cultured as T_rest_ with IL2 on days 0, 3, 6, and with tumor cells + peptide + IL2 on day 9 after plating. After 12 days (thus 3 days after last stimulation), cells were stained and analyzed by flow cytometry. Functional assays were performed the same or the next day normalized to cell numbers.

### Immunofluorescence staining for flow cytometry

At the indicated time points, T cells were stained with the following protocol. Cells are washed in PBS, resuspended in PBS, and blocked with 1:100 human Fc-receptor-inhibitor (eBioscience) in PBS and stained with Fixable Viability Dyes (Biolegend or eBioscience) 1:200 for 20 min on ice. For surface staining, cells were washed and resuspended in FACS buffer (PBS supplemented with 2 mM EDTA, 0.1% Na-Azide, 2% FCS), and stained with the appropriate antibodies for 30 min at 4 °C. All antibodies used in this study are listed above. For intracellular (cytoplasmic) staining, including SNX9 and cytokines, cells were fixed and permeabilized using IC Fixation Buffer (eBioscience) for 20 min at room temperature. Intracellular antibodies were then stained in 1x Permeabilization buffer (eBioscience) for 30 min at 4 °C. For secondary staining, this procedure was repeated, including washing steps. For staining of nuclear proteins, the Fixation/Permeabilization kit (eBioscience) was used for 30 min at room temperature followed by two wash cycles in 1x permeabilization buffer and antibody staining in 1x permeabilization buffer for 45 min at room temperature. We added 10’000 Precision counting beads (Biolegend) to each sample before the first washing step to adjust cell counts after acquisition based on the bead count (population high in SSC and positive in any channel <640 lasers). For the staining for phospho-AKT-Ser473, cells were fixed with IC fix for 20 min at room temperature and then permeabilized in a custom 0.1% Triton-X100+ 1% BSA in PBS buffer for 5 min. Then cells were stained in FACS buffer with antibodies as described above. After staining, cells were analyzed on a BD LSR Fortessa Cell analyzer (BD Bioscience), Cytoflex S (Beckmann) flow cytometer or an Aurora Spectra Analyzer (CyTek). Data were collected using the BD FACS Diva Software version 7 (for Fotessa), Beckmann Culture CytExpert, or SpectraFlow (for Aurora) and further analyzed with FlowJo v10.1.6 (Tree Star Inc.) and GraphPad Prism v8 (GraphPad Software Inc.). All results unless indicated show integrated fluorescence area on a biexponential scale.

### Flow cytometry-based cell sorting

For cell sorting, cells were kept on ice, washed in PBS, and stained with appropriate antibodies for 30 min at 4 °C in PBS+ 2% FCS and 2 mM EDTA (without Azide). Antibodies targeting CD14, CD11b, CD4 and CD19 were used to gate out potentially contaminating other immune cell populations. Following incubation, cells were washed, resuspended in the same buffer and filtered through a 70 μm mesh. Sorting of cells was performed using a FACSAria III or FACS SorpAria (BD), and the purity of sorted populations was routinely tested to be >98%.

### Degranulation and cytokine production assay

We performed a co-culture of peptide-loaded T2 cells with T cells in the presence of CD107a antibodies to assess the degranulation of cytokine production of T cells. For this purpose, T2 cells were incubated at a density of 1mio/ml with the respective peptides diluted in full RPMI supplemented medium. Afterward, 10’000 NY-ESO-1 specific T cells (measured by TCR Vβ13.1 staining as above) were co-cultured with 10’000 of these peptide-loaded T2 cells (1:1 E:T Ratio) for 5 h in the presence of 20 ng/ml anti-CD107a-PE antibody and 1x Monensin (Biolegend). Following incubation, the cells were stained for dead cells, surface antibodies, fixed using IC fixation buffer (eBioscience), and then stained for the accumulation of IFNγ and TNFα within cells (in 1x Permeabilization Buffer, eBioscience). Samples were analyzed on a Fortessa LSR (BD) or Cytoflex (Beckmann Coulter).

### Killing assay

Killing capacity was measured using a luminescence-based cell system. T2 cells expressing Luciferase and tdTomato (abbreviated “T2-Luc”) were generated by the transduction of a lentivirus made with the pFU-Luc2-tdTomato construct and sorting for tdTomato expression. T2-Luc2 cells were washed and resuspended at 1 mio/ml with 1 μM NY-ESO-9V peptides and incubated at 37 °C for 30 min. T2-Luc cells were then washed and plated at 20’000 cells per well of a V-bottom 96-well white plate. 20’000 (or else according to the E:T ratio) NY-ESO-1 specific T cells were then added to these cells and incubated for the indicated time. Afterward, 0.15 mg/ml D-Luciferase (Perkin Elmer) was added to each well and immediately analyzed on a BioTek H1 Spectro/luminometer, acquiring for 1 sec / well. Averages of three successive reads were used. Controls without T cells (maximal signal) and one with 0.1% Triton-X100 (minimal signal) were used to calculate % specific lysis:

$$\%\,{specific}\,{lysis}=\left(1-\left(\frac{{signal}-{minimal}\,{signal}}{{maixmal}\,{signal}-{minimal}\,{signal}}\right)\right)\times 100\%$$

### Proliferation and stability assay

To measure proliferation and stability after repetitive stimulation, cells on day 13 after the first stimulation, were washed and plated at 10’000 specific cells per 96-well with 50 U/ml IL-2. Cells were then either left to rest or stimulated again with a 1:1 ratio of irradiated NY-ESO-1 9 V loaded T2 tumor cells as above. The medium was exchanged three days later, and cells were stained and analyzed after another 3 days (total 6 days). The next day (7 days total), the degranulation capacity and production of cytokines of the cells was measured as above.

### PD-1 blockade in T_ex_ model

We assessed the responsiveness of Teff and Tex (thus both 3 days post last stimulation), which both upregulate PD-1, to PD-1 blockade in a co-culture assay with the MDA-MB-231 cell line. This breast cancer cell line naturally expresses high levels of PD-L1 and is HLA-A2+, which allows for loading of NY-ESO-1 peptides^88^. We seeded 5000 MDA-MB-231 cells per flat bottom 96-well the day before the assay in 100 μl IMDM complete medium (same supplements as for other media, 10% FCS). The next day, 10 nM NY-ESO-1 9 V peptide and 10 U/ml recombinant human IL-2 in 100 μl human serum RPMI medium (see above) was added to these wells. Next, T_eff_ or T_ex_ were added either at an effector to target E:T ratio of 1:1 for the killing readout or 4:1 for the IFNγ ELISA readout (to stay in detectable range for both assays). Cells were then co-cultured for 4 days. The supernatant of the 4:1 E:T ratio co-culture was used to measure IFNγ by ELISA (BD). Tumor cell killing was evaluated using an MTT assay. Briefly, wells were washed carefully with PBS, then incubated with fully supplemented RPMI containing 0.5 mg/ml MTT ((3-(4, 5-dimethylthiazolyl-2)−2, 5-diphenyltetrazolium bromide, Sigma) for 2 h. Then solution was then aspirated carefully and the deposited color solubilized using 90 μl of DMSO (Sigma). Absorption at 550 nm was then measured using a BioTek H1 plate reader. Specific lysis was calculated as above (killing assay).

### Transcriptomics analysis

Human Healthy donors CD8 T cells from four donors were isolated by CD8 MACS isolation. Cells were stimulated and transduced as described above for the ex vivo exhaustion model. 12 days after the first stimulation with tumor cells and peptide these T_rested_, T_tumor_, T_ex_ were sorted for TCR Vβ13.1+ (NY-ESO-1 TCR) CD8 CD56- CD4- DAPI- cells. Minimally 415’000 (most samples 600’000) T cells were sorted into cold FACS buffer, then centrifuged at 500 g for 8 min, and washed by centrifugation with cold PBS. Cells were then resuspended in 750 μl Trireagent (Sigma). Total RNA were purified using the kit Direct-Zol RNA Miniprep (Zymo-Research, Cat# R2050). The purified RNA was quality-checked on the Bioanalyzer instrument (Agilent Technologies, Santa Clara, CA, USA) using the RNA 6000 Pico Chip (Agilent, Cat# 5067-1513). RNA was quantified by Fluorometry using the QuantiFluor RNA System (Cat# E3310, Promega, Madison, WI, USA). Library preparation was performed, starting from 200 ng total RNA, using the TruSeq Stranded mRNA Library Kit (Cat# 20020595, Illumina, San Diego, CA, USA) and IDT TruSeq RNA UD Indexes. Libraries were quality-checked on the Fragment Analyzer (Advanced Analytical, Ames, IA, USA) using the Standard Sensitivity NGS Fragment Analysis Kit (Cat# DNF-473, Advanced Analytical). Samples were pooled to equal molarity. The pool was quantified by Fluorometry using the QuantiFluor ONE dsDNA System (Cat# E4871, Promega, Madison, WI, USA). Sequencing was performed on the Illumina Novaseq 6000 platform to produce paired-end 51nt reads. Read quality was assessed with the FastQC tool (version 0.11.5). Reads were mapped to the human genome (hg38) with STAR (version 2.7.9a)^89^ with default parameters, except filtering out multimapping reads with more than 10 alignment locations (outFilterMultimapNmax=10) and filtering reads without evidence in the spliced junction table (outFilterType = “BySJout”). Gene expression was quantified with featureCounts from the Subread package (v2.0.1)^90^ using gene annotation from Ensembl release 105, and options *“-O -M–read2pos* = *5–primary -s 2 -p -B*” to count the number of reads (5’ ends) overlapping with the exons of each gene assuming an exon union model.

The results of the RNA sequencing have been deposited on Gene Expression Omnibus (GEO) under the number GSE210534. The analysis pipeline is deposited on Github/Zenodo (10.5281/zenodo.7307407) and more information is found in Supplementary Data 1–4. Briefly, transcript count data was analyzed in R 4.2.1 using edgeR_3.38.4, limma_3.52.2, org.Hs.eg.db_3.15.0, TxDb.Hsapiens.UCSC.hg38.knownGene_3.15.0, tidyr_1.2.0, dplyr_1.0.9, ggrepel_0.9.1. Transcripts coding for protein coding genes were normalized for library size. A donor-paired design matrix an edgeR^91^ functions glmQLFit, glmQLFTest, and topTags were used to investigate all significantly dysregulated genes among any conditions (ANOVA-like, Supplementary Data 1) or between each combination of conditions (Supplementary Data 2). A Benjamini-Hochberg adjusted p-value cutoff of <0.01 and log2-fold-change cutoff of 0.75. Heatmaps and k-means clusters (n = 6) were created using ComplexHeatmap_2.12.0 from row-scaled counts per million (cpms). Gene sets from the comprehensive single-cell PanCancer T cell Atlas by Zheng et al.^37^ were retrieved. The top 100 genes per gene set (ranked based on their reported effect size) were extracted (Supplementary Data 3) to better account for the differences in gene set sizes. Gene set enrichment was calculated using the camera function in edgeR_3.38.1 and plotted using ggplot2_3.3.6. Heatmaps were generated using ComplexHeatmap_2.4.3.

### Selection of genes for the targeted CRISPR/Cas9 screen

To prioritize genes, we ranked all significantly upregulated genes in T_ex_ vs T_rest_ condition according to their overlap with published patient TIL datasets available at the time. We focused our analysis on genes with more than two counts per million reads after repetitive stimulation (T_ex_) and at least two-fold upregulation compared to T_rest_. Of this list of genes, we further selected genes based on literature review and excluded genes that were already highly studied in T cell exhaustion (for example, HAVCR2 and CTLA4) (Supplementary Data 4). In addition to the 29 selected genes, three genes known to be essential for T cell functionality (ZAP70, LAT, LAMP1) (Shifrut et al. 2018) were used as positive and negative controls for the CRISPR-Cas9 screen.

### Cloning gRNA CRISPR screen library

5 gRNA sequences for each of the selected genes and 20 intergenic controls were extracted from a published highly optimized gRNA library (Wang et al. 2017) and ordered as a DNA oligo pool with the required overlaps for assembly from Twist Biosciences according to Wang et al.^92^. gRNA DNA oligonucleotides were amplified by high fidelity PCR, purified over a 2% agarose Tris-acetate EDTA gel, and extracted using Machery Nagel PCR cleanup kit. LentiCRISPRv2-mCherry (Addgene 99154) was digested by BsmBI and cut plasmid extracted from a 1% TAE agarose gel. This fragment was fused with the PCR amplified gRNAs at a 1:30 molar ratio by Hifi-DNA Assembly for 1 h at 50 °C (New England Biolabs, failed using Gibson Assembly standard kit). The product was amplified by the transformation of Stbl3 *E. coli* with over 1000 colonies per guide, cultured in LB with 100 μg/ml ampicillin (Sigma), and extracted using Midi Prep (Machery Nagel). The plasmid library was barcoded by PCR and sequenced at the D-BSSE Genomics Facility on an Illumina MiSeq 50 cycle v2 run, which proved successful cloning and representation of all guides (Gini index 0.188). 2nd generation lentivirus was then prepared on a larger scale (>1000 cells / guide) from the library and the NY-ESO-1 TCR, which were then concentrated by ultracentrifugation and titrated on Jurkat cells.

### CRISPR screen and analysis

Freshly isolated healthy donor CD8 T cells were stimulated as above. In addition, cells were co-transduced with LentiCRISPRv2-mCherry virus at an MOI of 0.5. For this purpose, non-treated 6-well polystyrene plates were coated with 2 μg/cm^2^ Retronectin (Takara) overnight in PBS at 4 °C and then blocked using 2% BSA in PBS for 20 min at room temperature. The LentiCRISPRv2-mCherry virus was diluted according to the desired MOI in PBS with 0.1% BSA and added to the plates in 2 ml per well. Plates were then centrifuged 90 min at 2000 g at 32 °C. Plates were then washed with 0.1% BSA in PBS, and CD8 T cells were added on top. Then the NY-ESO-1 TCR virus was added, and cells were incubated for 2 days. Fresh medium and 50 U/ml IL-2 was added every 2 days, and after 6 days, cells were sorted for live CD8+ TCR+ mCherry+ cells (co-transduction efficacy for the two donors was 0.57% and 0.99% respectively). Cells were then repetitively stimulated with NY-ESO-9V peptide, as described above. After four rounds of stimulation, cells were re-stimulated with NY-ESO-9V loaded T2 cells in the presence of an anti-CD107a-APC-H7 antibody. 4 h later, cells were stained and sorted based on live CD8+ Vβ13.1+ CD19- CD4- CD56- CD107a+/− fractions into 200ul PBS. Genomic DNA from these samples was extracted using the Qiagen Blood DNA mini kit, including a Proteinase K digestion step. DNA was eluted in 5 mM Tris-HCl (no EDTA), and a barcoded PCR amplification of gRNA sequences was performed as described^92^ with PCR cycles: 95 °C 2’; 35x: 98 °C 10”, 60 °C 15”, 72 °C 30”; 72 °C 5’. gRNA PCR product was isolated from a 2% agarose in Tris-Acetate-EDTA gel and eluted using Machery Nagel PCR cleanup kit and dried for 10 min at 56 °C. DNA was eluted in 25 μl 5 mM Tris-HCl. This DNA library was loaded onto a 50 cycle MiSeq v2 Illumina Run on an Illumina MiSeq.

gRNA sequencing reads retrieved from this sequencing were demultiplexed and uploaded to PinAPL-py.ucsd.edu^39^. PinAPL-py was then used to align, control for quality, and count guides with the 5’ adapter ATTTTAACTTGCTATTTCTAGCTCTAAAAC. Sequencing results are deposited under GSE190246. R Studio (R version 4.2.1) was then used to calculate the average log2 fold change per gene and donor (average over the 5 guides per gene) and plot these values. Genes were ranked based on median log2 fold change (of donor replicates).

### Cas9-RNP-mediated KO

To knock out genes using Cas9-crRNA-tracrRNA ribonuclear protein complexes, the Lonza Nucleofector 4D system was used. Both gene-specific Alt-R-crRNA and Alt-R-tracrRNA were mixed at 200 μM in nuclease-free duplex buffer (IDT) and heated to 95 °C for 5 min, then cooled to 25 °C at −0.1 °C per minute. 1.5 μl of these annealed crRNA-tracrRNA complexes was then incubated with 1.5 μl of 40 μM Cas9-NLS protein (Berkeley, QB3) for 30 min at room temperature in the dark and used immediately (referred to as Cas9-RNP). CD8 T cells were stimulated for 1 day using anti-CD3+ anti-CD28 stimulatory beads (Miltenyi) with 150 U/ml of IL-2 in 8% human serum containing supplemented RPMI as described above. The next day, beads were removed by magnetic separation. Cells were spun down at 500 g 3 min and resuspended in supplemented electroporation P3 buffer (1:4.5 supplement to buffer ratio) at 1 Mio / 20 μl according to manufacturer’s instruction. 20 μl of this solution was then mixed with the 3 μl of Cas9-RNP and 0.75 μl of 200 μM Electroporation enhancer. This mixture was then electroporated using the EH115 setting in an X-unit Lonza Nucleofector 4D. Immediately after electroporation, 80 μl prewarmed medium was added, and incubated for 20 min at 37 °C. Cells were then transferred to 24 well plates at 1 mio/ml in fresh medium with 50 U/ml IL-2, 1:4 bead to cell ratio of anti-CD3+ anti-CD28 stimulatory beads and if indicated additional NY-ESO-1 TCR virus at 1 MOI. Cells were then expanded 1:2 every two days with fresh medium and 50 U/ml IL-2. On day 6 post electroporation, cells were sorted for Vβ13.1+ cells using a mouse anti-Vβ13.1 antibody (Biolegend, H131 clone) and anti-mouse IgG microbeads (Miltenyi). Cells were rested overnight in fresh medium and 50 U/ml IL-2 and then stimulated either 1x with T2+ peptide and analyzed on day 3 (T_eff_) or repetitively for 4x T2+ peptide as described above (T_ex_).

### Immunofluorescence staining for imaging

To perform immunofluorescence images of T cell – tumor cell conjugates, T2 tumor cells were loaded with 1 μM NY-ESO-1 (9 V) peptide for 30 min at 37 °C. When indicated T2 cells were before also stained with 1 μM Cell Trace Violet or CFSE for 15 min in PBS at 37 °C, then washed in complete medium 2x before co-culture. Then T2 tumor cells were washed 2x in serum-free prewarmed RPMI and resuspended at 1 Mio/ml in the same medium. Repetitively stimulated NY-ESO-1 TCR transduced T cells produced as above, were washed 2x in serum-free prewarmed RPMI and resuspended at 1 Mio/ml. Both cells were then mixed at a 1:1 ratio and incubated for 5 min at 37 °C. 50 μl of this cell mixture was then plated per microscopy slide well (25'000 T cells and tumor cells respectively). Cells were then incubated on the slide for 30 min and then fixed with −20 °C 100% Methanol for 5 min. For LAMP1 and phalloidin stainings, cells were instead fixed for 20 min at room temperature in 4% PFA (Electron Microscopy Services, diluted with PBS), extracted with 0.1% Triton-X100 in PBS for 5 min and quenched with 50 mM Glycine in PBS for 20 min. For the images of CD28 and TCRz, Teff cells (3 days poststimulation with T2-peptide) were electroporated with 800 ng of p-human-TCRzeta-EGFP or p-human-CD28-EGFP plasmids using the EH115 P3 protocol as described above. The cells were then rested in 50 U/ml IL2 in human serum medium for 24 h, before the co-culture with T2 tumor cells as described above.

Both fixation procedures were then followed by blocking in 1% 0.2 μm filtered bovine serum albumin (Sigma) in PBS (blocking buffer) for 15 min. Primary antibodies were then incubated in the same blocking buffer for 1 h at room temperature or overnight at 4 °C. Samples were then washed 4x times with blocking buffer, incubated for 2×5 min in blocking buffer, and then incubated for 1 h with secondary antibody in blocking buffer at room temperature. For the amplification of EGFP’s signal, it was counterstained with chicken anti-GFP followed by anti-chicken-AF488 antibody. If indicated, samples were washed again as above and incubated with 1:500 dilution of DAPI in blocking buffer for 5 min. Samples were washed again and mounted with Vectashield Vibrance mounting medium and Nr. 1.5 coverslips and sealed with clear nail polish. Samples were stored at 4 °C until acquisition.

### Imaging

Images from immunofluorescence images were recorded on a Nikon Ti with a Yokogawa CSU-W1 spinning disk module on a Photometrics 95B (22 mm back-illuminated sCMOS) camera. A Nikon CFI Apo TIRF NA 1.49 100x objective together with a 1.5x additional magnification unit was used with 1.515 NA oil mounted samples. Diode-pumped solid-state lasers at 405, 488, 561, and 647 nm were used together with filters for DAPI (ET460/50 nm), AF488 (ET525/50 nm), AF568 (ET630/75 nm) and AF647 (ET700/75 nm) with a Quad BS Dichroic mirror. For the actin, LAMP1, and perforin stainings, raw nd2 format image stacks of 110x110x100nm were deconvoluted using Huygens using a theoretical point spread function, classical maximum likelihood estimation using 100 iterations and a quality stop criterion of 0.01. Images were visualized using Imaris 9 (Bitplane) and OMERO (www.openmicroscopy.org, managed by Biozentrum Basel) was used to generate figures. Single slices or sections are shown with linear display adjustments (brightness). All recorded images in single colors are shown in in Supplementary Figs. 1, 2.

### Immunoblotting (Western blot)

T cells were collected and washed 2x in ice-cold PBS and then lysed in 8 M urea (in H_2_O, Cell Signaling #7900) supplemented with 0.5% Triton-X100 (Merck #1.08643.1000), 1× cOmplete mini protease inhibitor cocktail (Roche #11836153001). DNA was sheared by sonication, then the DNA was pelleted before samples were complemented with 5x Laemmli buffer (2% SDS, 5% 2β-mercapto-ethanol, 10% glycerol, 0.002% bromophenol blue in 62.5 mM Tris-HCl) and boiled at 95 °C for 5 min. Denaturized proteins and Precision Plus Protein Dual Color Standards (BioRad #1610374) were separated by SDS-PAGE and later transferred to a PVDF membrane (Immobilon-P, Sigma #IPVH85R) at 100 V for 60 min in tris/glycine buffer (BioRad #1610771) supplemented with 20% methanol. The membrane was blocked for 1 h at room temperature with 5% BSA in TBS-T (TBS with 0.05% Tween 20). Membranes were incubated with primary antibodies overnight at 4 °C followed by an incubation IRDye secondary antibodies (LI-COR #925-68070 and #925-32211) for 1 h at room temperature. The following primary antibodies were used: anti-ERK2 (SCBT #sc-1647, as a loading control) and anti-SNX9 (ThermoFisher #PA-5-56734). All primary antibodies were used at a dilution of 1:1000 in TBS-T with 5% BSA. Blots were scanned by with a LI-COR Odyssey CLx imager. Signal intensities were quantified by ImageJ (Fiji). Full blot scans are found in the source data file for the linked figures.

### OTI KO generation

For the generation of murine antigen-specific Snx9 KO T cells, we harvested spleen and axial, cervical and inguinal lymph nodes from “OTI” mice (C57BL/6-Tg(TcraTcrb)1100Mjb/J, RRID:IMSR_JAX:003831), in which all CD8 T cells recognize ovalbumin (OVA257-264, H-2Kb). Spleens and lymph nodes were strained over a 70 μm strainer and washed with PBS, incubated in red blood cell lysis buffer for 1 min and wash with PBS again. Cells were then resuspended at 1.5 Mio/ml in murine T cell medium (supplemented RPMI medium as above for human T cells, but with 10% heat inactivated FCS instead of human AB+ serum). Cells were then stimulated by addition of 100 ng/ml SIINFEKL (Invivogen, OVA257-264) and 100 U/ml recombinant human IL2 (cross-reactive with murine IL-2 receptor; Proleukin, Clinigen Healthcare) and incubated at 37 °C. After two days of stimulation, 900 pmol Alt-R-crRNA (sgSNX9_9 and sgSNX9_IDT_AF; IDT) and 900 pmol Alt-R-tracrRNA (IDT) were annealed by heating to 95 °C for 5 min, then cooled to 25 °C at −0.1 °C per minute. 1.5 μl of these aligned crRNA-tracrRNA complexes was then incubated with 15 μl of 40 μM Cas9-NLS protein (Berkeley, QB3) for 30 min at room temperature in the dark and used immediately (referred to as Cas9-RNP). 2 days after stimulation, 10mio of the splenocytes and lymphocyte mixture was collected per condition, spun at 90 g for 10 min and then resuspended in 100ul P4 nucleofection buffer, the Cas9-RNPs and electroporated with the CM137 program. Immediately 900ul medium was added and rested for 20 min at 37 °C. Then cells were transferred to wells at 1.5 Mio/ml with 100 U/ml IL-2. Cells were expanded to reach 1.5 Mio/ml with fresh 100 U/ml IL-2 daily for 4 days. Cells were then washed in PBS and 1.5 Mio or 2.7 Mio were transferred per mouse as indicated.

### Calcium flux

For Ca^2+^ flux measurements, T_eff_ were used (1x T2-peptide stimulation of the repetitive stimulation procedure described above, then used on day 3 post stimulation). Black well, clear bottom μClear 96-well polystyrene plates were coated with 0.01% Poly-L-Lysine for 1 h at room temperature, then washed 3x in distilled water and dried for >2 h. Stimulatory beads were prepared from the Human T cell Activation and Expansion Kit (Milteniy) by coating anti-CD2/3/28 antibodies each at 10 μg/ml in MACS buffer (0.5% FCS, 2 mM EDTA in PBS) overnight onto the beads in a rotator at 4 °C. anti-CD2 was included to increase binding strength of cells to the beads. On the day of the assay, cells were loaded for 45 min at 37 °C in a solution consisting of phenol free RPMI (base medium, supplemented with 1% PenStrep, 1 mM Pyruvate, 1% NEAA, 10 mM HEPES) with 0.04% PluronicF127 (Thermo Fisher) and 2 μM Calbryte520-AM (AAT Bioquest). Cells were then washed 2x in base medium with 2% FCS (termed assay medium) and plated at 250’000–500’000 cells per well in assay medium onto the Poly-L-Lysine coated plate and incubated for 30 min at 37 °C for cell attachment. Beads were washed 2x in assay medium and rapidly added to each well at 1.5 Mio beads per well (approx. 4:1 ratio). The plate was then recorded immediately in a BioTech H1 fluorescence reader at 490 nm excitation and 525 nm absorbance for 30 min. The signal for each timepoint was normalized to the first measurement (seconds after the bead addition) to adjust for baseline differences. Normalizing to a read acquired before bead addition proved to be less accurate, because of differences in changes of fluorescence by addition of beads. The normalized signal was the visualized in Graphpad Prism and the area under the curve (AUC) and maximum increase in intensity calculated for the first 30 min after beads addition.

### NFAT nuclear translocation assay

To measure NFAT activation, non-treated flat bottom 96-well plates were coated with 1 μg/ml anti-CD3 (OKT3) or 1 μg/ml anti-CD3 plus 2.5 μg/ml anti-CD28 (CD28.2) in PBS overnight at 4 °C. Wells were then washed with PBS. T_eff_ were collected, counted and resuspended in fresh human T cell medium. 200’000 cells were then seeded per well and the plate was centrifuged for 1 min at 500 g. Cells were then incubated for 3 h at 37 °C in the incubator. Then cells were resuspended, the solution was transferred, the wells washed with FACS buffer and both fractions combined in one V-bottom well per condition. The cells were centrifuged and resuspended in cold FACS buffer with 1:50 dilution of anti-CD8 (SK1, Biolegend) in FACS buffer and incubated for 10 min on ice. Cells were then washed twice in FACS buffer and then fixed with 4% PFA in PBS and incubated for 20 min at RT. Cells were then washed with permeabilization wash buffer (PBS with 0.1% TritonX100 (Sigma), 1% BSA and 0.01% Sodium Azide) 2 times. Then the cells were stained in FACS buffer sequentially for 1:800 NFATc2 (D43B1 clone, CST) and then with 1:500 anti-rabbit IgG (highly cross absorbed, Thermo Fisher) and DAPI 5 μg/ml, each step for 30 min at RT. Cells were then washed twice in FACS buffer and then acquired on an Imagestream MKII imagestream instrument using automated plate handling. Between 1000–5000 single (aspect ratio M01 > 0.5) CD8+ cells were recorded. Cells were then gated for focused, DAPI+ CD8+ NFATc2+ cells. The nuclear localization wizard was used to quantify the Similarity_Morphology metric between DAPI and NFATc2 for each cell. The geometric mean of the distribution of this similarity metric was used to compare the different conditions.

#### Signaling flow cytometry

To determine the phosphorylation of AKT upon stimulation, we used T_eff_ cells+/− SNX9 KO (thus stimulated three days before with tumor cells, peptide, and IL2). 96-well flat bottom plate wells were coated with either 1.25 μg/ml anti-CD3 (OKT3) and 2.5 μg/ml anti-CD28 (28.2 clone) antibodies (low CD3+ CD28 condition), or with 5 μg/ml anti-CD3 (OKT3) alone overnight at 4 °C in PBS. Wells were washed with PBS and replaced with 100 μl human Serum medium as described above. T cells were washed in medium and then added to the plates, spun for 10 seconds at 500 g and then incubated for 30 min. Then cells were rigorously resuspended using ice cold FACS buffer (PBS with 2% FCS, 5 mM EDTA, and 0.1% Sodium azide) and spun at 4 °C. Cells were then fixed for 10 min using IC fix at RT. Then cells were permeabilized using self-made Perm-Wash buffer (PBS with 0.1% Triton-X100 and 0.1% BSA) for 10 min at RT. Cells were then washed 2x and stained in FACS buffer with the indicated antibodies (AKT-phospho-Ser473, clone 98H9L8, Thermo Fisher) and counterstained using PE-labeled anti-rabbit IgG polyclonal highly cross absorbed antibody (Thermo Fisher). Cells were measured using a BD Fortessa.

To determine phospho-PLCγ1, we had to change the assay procedure due to difficulties to detach T cells from the plate after stimulation and the very dynamic signaling through PLCγ1. Therefore, we rested T cells+/− SNX9 KO at 6 days post electroporation, in fresh human serum medium and 10 U/ml IL2 overnight. Then we stained the cells at RT for 5 min with fixable viability dye eF450 before the assay. Afterwards we washed and incubated them in serum-free cytokine-free medium (RPMI with other supplements as above but without serum, but with 0.1% BSA to reduce attachment) for 1 h at 37 °C. Then cells were stained on ice with low anti-CD3+ anti-CD28 (1.25 μg/ml OKT3+ 1.25 μg/ml CD28.2) or high anti-CD3 (2.5 μg/ml OKT3) in medium for 15 min. Then cells were washed and stained on ice with 10 μg/ml anti-mouse IgG (Jackson, to crosslink antibodies for activation) for 15 min and then washed Afterwards, cells were resuspended in 37 °C warm medium and immediately added to a pre-warmed metal plate in an 37 °C incubator (activation). After 5 min, cells were immediately fixed by the addition of an equal volume of IC fix and incubate for 20 min at RT. Then cells were spun and resuspended in 20 μl FACS buffer. Then cells were permeabilized by the addition of 180 μl Methanol at −20 °C and incubated at −20 °C for 15 min. Cells were then 2x washed by centrifugation (from now on 1000 g 3 min) in FACS buffer, stained for antibodies (CD8 SK1 FITC and anti-PLCγ1-Tyr783 CST) in FASC buffer for 30 min and then counterstained with anti-rabbit antibodies as above. Cells were measured on a Beckmann Coulter Cytoflex.

### Quantitative real-time PCR

T_ex_ were purified by human CD8 microbeads (Miltenyi) according to manufacturer’s protocol. Cells were washed 1x by centrifugation at 500 g 3 min in cold PBS. RNeasy Plus Mini Kit (Qiagen; # 74136) was used to isolate total RNA from approx. 1 Mio T cells according to the manufacturer’s protocol. Isolated RNA was reverse transcribed using the iScript cDNA synthesis kit (BioRad; #170-8891). Quantitative real-time PCR was performed according to the manufacturer’s protocol using the PrimeTime Gene Expression Master Mix (IDT; #1055771), the equivalent of 12.5 ng cDNA, and PrimeTime qPCR assay probes (IDT) in a volume of 10 µl. A ViiA™ 7 Real-Time PCR System (Applied Biosystems) was used for the fluorescence readout. The following assay probes were used: *SNX9* (Hs.PT.58.21424684), *TOX* (Hs.PT.58.28002606), *TOX2* (Hs.PT.58.39787291), *NR4A1* (Hs.PT.58.39997829), *NR4A2* (Hs.PT.58.704850), *NR4A3* (Hs.PT.58.14945655), *LDHA* (Hs.PT.40245343*)* and the house keeping gene *HPRT1* (Hs.PT.58.v.45621572). Ct values of the house keeping gene were subtracted from the other transcripts Ct values, yielding a delta Ct value. This value equals the log2 fold change in transcript abundance shown in the figures.

### SCENITH

The SCENITH flow-based single cell metabolic analysis was performed according to and with reagents provided by Argüello et al^93^. T_ex_ (3 days after the fourth stimulation with T2 tumor cells and peptide) were collected, counted and washed in fresh T cell media (as described above 8% human serum) and plated at 150’000 cells per 96 V-bottom plate well. Cells were equilibrated in the incubator at 37 °C and 5% CO2 for 30 min. Then the drug solutions with 2-deoxyglucose (DG), oligomycin (O), the combination (DGO), or DMSO only (control) were prepared in the same medium and equilibrated in the incubator for 10 min. Then the drugs were added to the cells, mixed rapidly and incubated again. For the DGO condition, DG was added first for 10 min and then O was added for the last 5 min, according to the manufacturer’s instruction. After the 15 min total incubation time, the cells were washed 1x in PBS at 4 °C and then stained in PBS for Zombie NIR Viability Dye (Biolegend) and anti-CD8-BV605 (SK1 clone, Biolegend) for 20 min on ice. Cells were then fixed with the Foxp3 Transcription Factor Fixation Permeabilization Kit (eBioscience) for 20 min at room temperature (RT). Cells were then washed (500 g 3 min centrifugation) in 1x Permeabilization buffer (eBioscience) and incubated in blocking solution (1x Permeabilization buffer with 10% final concentration of FCS) for 10 min at RT. Then 1:250 diluted anti-Puromycine-AF488 antibody (Gift from R. Argüello) was added on top in blocking buffer and incubated for 1 h at 4 °C. Cells were then washed (1000 g 5 min centrifugation) 2x with FACS buffer (described above, 2% FCS, 5 mM EDTA in PBS) and then kept in FACS buffer until acquisition on a Beckman Coulter Cytoflex. The following formula was used to calculate the glucose and FAO/AAO dependence. DG inhibits glucose usage, thereby remaining ATP production measured by Puromycin incorporation is dependent on FAP/AAO. Background signal is obtained by inhibiting glucose usage (DG) and mitochondrial respiration (O, oligomycin).

$${glucose}\,{dependence}=1-{FAO}\,{or}\,{AAO}=\frac{{control}-{DG}}{{control}-{DGO}}$$

### scRNASeq Sade-Feldman et al. reanalysis

We obtained the scRNAseq dataset described in Sade-Feldmann et al. under GEO accession number GSE120575 and analyzed in R 4.0.2, SingleCellExperiment_1.18.0, ggplot2_3.3.5 and scater_1.16.2. As described in their original publication, genes were filtered for protein coding genes and min expression of >4.5 logcounts in at least 10 cells. Cells were filtered to have >2.5 mean logcounts of their listed housekeeping genes and >0 logcounts for PTPRC (CD45) to exclude non-immune cells. Cells with a high fraction mitochondrial reads (>3 standard deviations from the mean; dying cells) and very many detected genes (>4 standard deviations from the mean; doublets) were excluded. PCA and UMAP dimension reductions were calculated as above. CD8 T cells were defined as having at least 1 read of *CD3E* and *CD8A* or *CD8B*; and having no detected read for *NCR1*, *NCAM1*, *CD4* (excluding NKs and CD4 T cells). A tSNE dimensionality reduction (based on PCA components and perplexity of 30) was calculated for these CD8 T cells. tSNE plots with selected transcripts highlighted as dot size and color were generated using ggplot2. SNX9 positive CD8+ T cells were additionally defined as having > 1 logcount for *SNX9*. The percentage of SNX9+ cells among all CD8+ T cells before immunotherapy was exported and visualized in Graphpad Prism according to treatment response.

### scRNASeq library preparation of intratumoral OTI cells

OTI T cells with or without Snx9 KO were generated as described in “OTI KO generation” and confirmed using flow cytometry staining. As above CD57BL/6 mice were injected subcutaneously with 1 Mio MC38-OVA and 1.5 Mio OTI T cells were transferred intravenously 12 days post tumor injection. After 12 days, the tumors (7 per condition) were removed separated from fibrous, necrotic and lymphoid tissue and cut into pieces of 1mm^3^. These pieces were then digested in the digestion mix as described above and dissociated using a gentle MACS dissociator (Miltenyi, using a C-tube, program m_impTumor_02). The mixture was then incubated for 15 min on a rotating shaker (200 rpm) at 37 °C. Then the mixture was again dissociated using the m_impTumor_03 program on the gentle MACS dissociator. Cells were then strained through a 100 μm MACS SmartStrainer (Miltenyi) and washed using PBS with 2% FCS. Cells were stained for CD45 (30-F11 clone in V450, BD 560501) and CD19 (1D3 clone in FITC, BD 553785) and analyzed for lymph node contamination. Samples with more than 5% CD19+ cells among CD45+ live cells were excluded due to lymph node contamination (3 samples: 2 intergenic and 1 Snx9 KO). The remaining samples were pooled into 2 pools (2–3 mice) per condition (intergenic n = 5 mice, *Snx9* KO n = 6 mice). These pools were sorted for DAPI- CD3e+ F4/80- Ly6G- CD19- CD8a+ CD45.1+ CD45.2- single cells using an AriaIII sorter (intergenic A: 6787 cells, intergenic B: 1975 cells; Snx9 KO A: 6163 cells, Snx9 KO B: 2295). Sorted single cells were then loaded onto a 10x Genomics Chromium NEXT GEM chip G. Libraires were prepared following the 10x Genomics protocol for 3′ gene expression profiling (CG000315, Rev C) and cDNA or library quality was assessed using a 4200 TapeStation System (Agilent). Sequencing was performed on Illumina Novaseq 6000 platform to produce paired-end 101nt R2 reads. Read quality was assessed with the FastQC tool (version 0.11.5). Sequencing files were processed with STARsolo (STAR version 2.7.9a)^94^ to perform sample and cell demultiplexing, and alignment of reads to the mouse genome (mm10) and UMI counting on gene models from Ensembl 102. The options “*–outFilterType* = *BySJout–outFilterMultimapNmax* = *10–outSAMmultNmax* = *1–outFilterScoreMin* = *30–soloCBmatchWLtype* = *1MM_multi_Nbase_pseudocounts–soloUMIlen* = *12–soloUMIfiltering* = *MultiGeneUMI_CR–soloUMIdedup* = *1MM_CR–soloCellFilter* = *None*” were used for STARsolo. For each sample, empty droplets were detected and removed using the *emptyDrops* function from the Bioconductor DropletUtils package (version 1.14.0; using 5000 iterations, the option *test.ambient* = *TRUE*, a lower threshold of 100 UMIs and an FDR threshold of 0.1%)^95^.

### Analysis of intratumoral OTI scRNAseq

Transcript counts per cell were used for downstream analysis in R version 4.2.1 (within R Studio for Mac 2022.07.01) using Seurat_4.1.1^96^, scater_1.24.0, viridis_0.6.2, future_1.27.0, dittoSeq_1.8.1. The data is deposited under GEO210535, the full analysis pipeline can be found on GitHub/Zenodo [10.5281/zenodo.7307407] and supplementary information is found in Supplementary Data 5. Briefly, Seurat was used to log10 normalize counts and the number of unique features, RNA counts, and the percentage of mitochondrial transcripts were calculated. biomaRt_2.52.0 was used to find the murine orthologues for the human cell cycle genes provided in Seurat. Cells with fewer then 2000 unique RNAs, less than 5000 transcripts, or over 15 percentage of mitochondrial transcripts were discarded. For the remaining cells (n = 3405 intergenic and n = 3612 *Snx9* KO), a FeatureScore for cell cycle genes (S phase and G2M phase), histone genes and interferon-stimulated genes together with the number of unique features per cell were used to scale the data (ScaleData). The ‘future’ Bioconductor package was used for parallelization. UMAPs were generated from the first 20 principal components and clusters defined with a resolution of 0.5. Cells from the two different pools per condition were merged. Signatures were retrieved from original publications: Andreatta^97^, Schietinger^32^, and Miller^98^. ggplot2_3.3.6, ggrepel_0.9.1, tidyr_1.2.0 and Seurat_4.1.1 were used to create visualizations. FindMarkers in Seurat was used to calculate differentially expressed genes for each cluster and for all cells in total between *Snx9* KO and intergenic.

### Generation of T2 CD80 CD86 KO and co-culture assays

Low passage T2 cells (source DSMZ) were electroporated with crRNA-tracrRNA-Cas9 complexes targeting human CD80 and CD86 (see sequences above) using the CA148 program in SE buffer at 0.4 Mio cells / 20 μl in a 16-well strip cuvette. Alt-R crRNA was obtained from IDT, Cas9-NLS protein from Q3 Macrolab (Berkeley). Cells were then expanded for 7 days before sorting for CD80- CD86- live cells using an AriaIII sorter (Beckmann). Cells were then expanded for 7 days before resorting for highly pure CD80- CD86- cells. For the co-culture assays with T2 wt and T2 CD80 CD86 KO cells, T_eff_ cells (day 3 post first stimulation with T2 wt + NY-ESO-1 9 V peptide) with or without SNX9 were washed and reseeded at 1mio cells per ml in 10 U/ml IL2 in human serum medium as above. After 24 h of resting at 37 °C, the cells were co-incubated with T2 wt or T2 KO cells at an E:T ratio of 1:2 in presence of 100 nM NY-ESO-1 9 V peptide. Additionally, either 10 μg/ml human IgG1 (Ultra-LEAF, Biolegend 403502) or Ipilimumab (clinical grade, Yervoy, BMS) was added to the culture. After 14 h of incubation at 37 °C, the co-culture was stained for CD25 in addition to CD8 and Zombie NIR. Fixed stainings (IC fix, eBiosciences) were acquired on a Cytoflex (Beckmann Coulter).

### Cell-free anti-CD3 anti-CD28 stimulation assays

Fresh *SNX9* KO and control CD8+ human T cells were generated by electroporation as described above but without TCR transduction. SNX9 KO efficacy was confirmed by flow cytometry staining 4 days post electroporation. Cells were expanded 1:2 every 2 days in 150 U/ml IL-2 containing human serum RPMI medium as above. 6 days post electroporation, the cells were removed from the magnetic stimulatory beads, washed and reseeded at 1mio/ml cells with 10 U/ml IL2 and rested for 24 h. These rested cells, were then restimulated on non-treated flat bottom 96-well plates coated for 2 h at 37 °C with the indicated concentrations of Ultra-LEAF purified anti-CD3 (OKT3, Biolegend Cat Nr. 317347) and/or anti-CD28 (CD28.2, Biolegend Cat Nr. 302943). Cells were then stained for CD25, CD8 and Zombie NIR, fixed and acquired on a Cytoflex (Beckmann Coulter).

### CAR T cell production and adoptive transfer

To generate human CD8+ CAR T cells, we isolated CD8+ T cells from heathy donor PBMCs using the CD8 human microbead MACS kit according to manufacturer’s instruction. Cells were then cultured in RPMI-1640 (Sigma) with 10% heat inactivated human male AB+ serum with 1 mM Sodium Pyruvate (Sigma), 2 mM Glutamine (contained in RPMI formulation), 10 mM HEPES (Gibco), 5 mM beta-mercaptoethanol (Gibco), 1% PenicilinStreptomycin (Sigma). Cells were stimulated on the same day with 1:1 ratio of CD3/CD28 beads (Human T cell Activation and Expansion kit, Miltenyi) and 150 U/ml rh-IL-2 (Proleukin). The next day, cells were collected into a falcon tube and 4 μg/ml Polybrene (Sigma) was added together with VSV-g pseudotyped lentivirus encoding an anti-human-CD19-FMC63vH chimeric antigen receptor with a CD28 transmembrane domain and a CD28 and CD3ζ signaling domain with a c-terminal T2A self-cleaving copGFP protein (anti-CD19-CD28z-T2A-copGFP). For the anti-CD19-CART19-BBz the same procedure was performed with a pLV-EFS-FMC63-BBz-P2A-mCherry CAR construct encoding the same single chain variable fragment targeting human CD19 but coupled to a CD8 transmembrane domain and CD3zetta and 4-1BB signaling domains. The cell - lentiviral mixture was centrifuged for 90 min at 1000 g (spinfection) and the resuspended and plated for 24 h at 37 °C. Then cells were expanded 1:2 every 2 days for 2 iterations with fresh medium and 50 U/ml rh-IL-2. Cells were then stained for CD8 (CD8-APC SK1 clone, Biolegend) and DAPI (Sigma) and analyzed for GFP+ cells (mCherry+ for BBz-CARs) using a Cytoflex flow cytometer. Cells were then counted, washed by centrifugation at 500 g 3 min in PBS, adjusted in volume for equal numbers of CARs (based on GFP/mCherry positivity), and transferred in PBS intravenously to NSG mice subcutaneously injected 3 days before with 0.5 Mio Raji (ATCC) in 8–12 mg/ml Matrigel (Corning, standard formulation).

### Serum protein analyses

Serum was collected from mice from the tail once a week into Monovette 200 Z-Gel (Sarstedt, Cat Nr. 20.1291) tubes, centrifuged as instructed and frozen to −80 °C.

For the serum analysis after OTI transfer, the Legendplex MurineVirusResponse (13-plex, Biolegend Cat. Nr. 740621) was used according to the manufacturer’s instructions and measured on a Fortessa (BD). The Biolegend Legendplex analysis online tool was used to calculate concentrations according to internal standards. TNFα and IFNβ were not detected but can be found in the source data.

For the CAR T cell experiments, the Legendplex Human CD8/NK Panel (13-plex, Biolegend Cat Nr. 741065) was used according to the manufacturer’s instruction and measured on a Fortessa (BD). Analytes that were not detected in fraction of samples were not displayed in figures, but can be found in source data (IL4, IL17, TNFα, sFAS, Granzyme A, Granzyme B). sFasL was not displayed as it only correlated with tumor size (also in source data). Of note, although we used a Legendplex kit for the detection of human proteins (which binds proteins using two anti-human antibodies per analyte), we cannot exclude that this kit also cross-recognizes the murine orthologues.

### Statistics and reproducibility

The statistical analysis and graph preparation were performed using the software package Prism version 8.0a (GraphPad Software, La Jolla, CA). Functional data and microscopy images are representative of at least two experiments each with different human donors. Recorded microscopy images including representative images shown in single channels are found in the Source Data file. Data is displayed as scatter dot plots where applicable and single points represent different healthy donor replicates. Data were considered statistically significant with p values <0.05. Normality tests were used to choose parametric or non-parametric tests. Data are shown as mean ± standard deviation with symbols representing individual patients or donors where applicable. All t-tests were performed as two-sided tests.

### Materials availability

This study did not generate unique new reagents. Plasmids are listed in Supplementary Table 1 and can be requested at the indicated sources. Antibodies and CRISPR crRNAs are available at the indicated sources in Supplementary Tables 2, 3.

### Reporting summary

Further information on research design is available in the Nature Portfolio Reporting Summary linked to this article.

## Supplementary information

Supplementary Information

Peer Review File

Description of Additional Supplementary Files

Supplementary Data 1

Supplementary Data 2

Supplementary Data 3

Supplementary Data 4

Supplementary Data 5

Reporting Summary

## Data Availability

The data for this manuscript has been deposited as a super series to the Gene Expression Omnibus (GEO) under the accession number GSE190247. Within this superseries, processed data for the bulk RNA sequencing of the Tex model are available under accession code GSE210534, raw and processed data from the CRISPR-Cas9 screen gRNA sequencing under accession code GSE190246, and raw and processed data for single cell sequencing of intratumoral OTI T cells with or without Snx9 KO under accession code GSE210535. For the bulk RNA sequencing of the Tex model, raw sequencing FASTQ files are available through the European Genome-Phenome Archive under accession number EGAS00001006794. The data are protected and available under restricted access according to the data security guidelines of the University of Basel. Access can be obtained by contacting dac@unibas.ch. The Sade-Feldman et al. publicly available data^17^ used in this study are available in the GEO database under accession code GSE120575. The Satpathy et al. publicly available single cell ATACseq data used in this study are available on the WashU EpiGenome Browser under http://epigenomegateway.wustl.edu/legacy/?genome=hg19&session=7UZG0iF90b&statusId=807471043. The remaining data are available within the Article, Supplementary Information or Source Data file. Source data are provided with this paper.

## Source data

Source Data

## Code availability

Code generated for this manuscript has been deposited in a Github/Zenodo repository and can be accessed without restrictions under the Zenodo accession number (7307407) [10.5281/zenodo.7307407].
