# Supplementary material for: Deletion of SNX9 alleviates CD8 T cell exhaustion for effective cellular cancer immunotherapy
Source: Nat Commun. 2023 Feb 2;14:86. doi: 10.1038/s41467-022-35583-w (PMC9895440; doi:10.1038/s41467-022-35583-w)
Supplement: Supplementary file 1 — Supplementary Information [file 41467_2022_35583_MOESM1_ESM.pdf]

Supplementary Information

Deletion of SNX9 alleviates CD8 T cell exhaustion for effective cellular cancer immunotherapy

Marcel P. Trefny[1*], Nicole Kirchhammer[1], Priska Auf der Maur[2], Marina Natoli[1], Dominic Schmid[1], Markus Germann[1], Laura Fernandez Rodriguez[1], Petra Herzig[1], Jonas Lötscher[3], Maryam Akrami[1], Jane C. Stinchcombe[4], Michal A. Stanczak[5], Andreas Zingg[5], Melanie Buchi[1], Julien Roux[6,7], Romina Marone[8,9], Leyla Don[1], Didier Lardinois[10], Mark Wiese[10], Lukas T. Jeker[8,9], Mohamed Bentires-Alj[2], Jérémie Rossy[12], Daniela S. Thommen[1,13], Gillian M. Griffiths[4], Heinz Läubli[5,11], Christoph Hess[3,14], and Alfred Zippelius[1,11*]

[1]Laboratory of Cancer Immunology, Department of Biomedicine, University of Basel and University Hospital of Basel, Basel, Switzerland; [2]Laboratory of Tumor Heterogeneity, Metastasis and Resistance, Department of Biomedicine, University of Basel and University Hospital of Basel, Basel, Switzerland; [3]Laboratory of Immunobiology, Department of Biomedicine, University of Basel and University Hospital of Basel, Basel, Switzerland; [4]Cambridge Institute for Medical Research, Biomedical Campus, Cambridge CB2 0XY, UK; [5]Laboratory of Cancer Immunotherapy, Department of Biomedicine, University of Basel and University Hospital of Basel, Basel, Switzerland; [6]Bioinformatics Core Facility, Department of Biomedicine, University of Basel and University Hospital of Basel, Basel, Switzerland; [7]Swiss Institute of Bioinformatics, Basel, Switzerland; [8]Laboratory of Molecular Immune Regulation, Department of Biomedicine, University of Basel and University Hospital of Basel, Basel, Switzerland; [9]Transplantation Immunology & Nephrology, Basel University Hospital, Basel, Switzerland; [10]Department of Surgery, University Hospital Basel, Basel, Switzerland; [11]Medical Oncology, University Hospital Basel, Basel, Switzerland; [12]Biotechnology Institute Thurgau, University of Konstanz, Kreuzlingen, Switzerland; [13]Division of Molecular Oncology and Immunology, The Netherlands Cancer Institute, Amsterdam, The Netherlands; [14]Cambridge Institute of Therapeutic Immunology and Infectious Disease, Jeffrey Cheah Biomedical Centre, University of Cambridge, Cambridge CB2 0AW, UK

***Corresponding authors:** Marcel Trefny, Ph.D. and Alfred Zippelius, M.D., Laboratory of Cancer Immunology, Department of Biomedicine, University of Basel and University Hospital of Basel, Hebelstrasse 20, 4031 Basel, Switzerland, Phone: +41 61 265 23 55, marcel.trefny@unibas.ch or alfred.zippelius@usb.ch

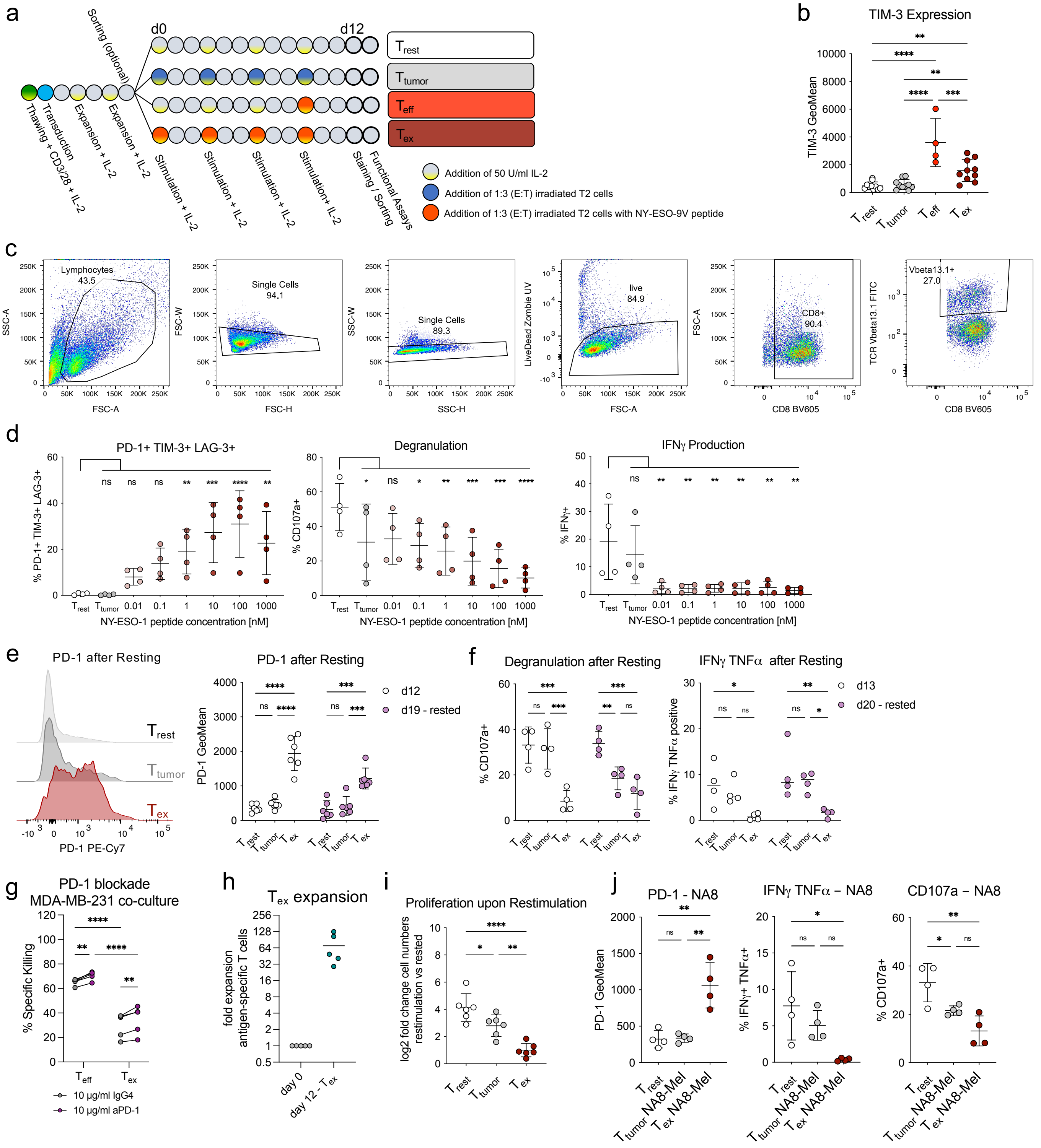

a
d0
d12
Sorting (optional)
Thawing + CD3/28 + IL-2
Transduction
Expansion + IL-2
Expansion + IL-2
Stimulation + IL-2
Stimulation + IL-2
Stimulation + IL-2
Stimulation+ IL-2
Staining | Sorting
Functional Assays
T rest
T tumor
T eff
T ex
Addition of 50 U/ml IL-2
Addition of 1:3 (E:T) irradiated T2 cells
Addition of 1:3 (E:T) irradiated T2 cells with NY-ESO-9V peptide
b
TIM-3 Expression
TIM-3 GeoMean
c
Lymphocytes 43.5
Single Cells 94.1
Single Cells 89.3
live 84.9
CD8+ 90.4
Vbeta13.1+ 27.0
SSC-A
FSC-A
FSC-W
FSC-H
SSC-W
SSC-H
LiveDead Zombie UV
CD8 BV605
TCR Vbeta13.1 FITC
d
PD-1+ TIM-3+ LAG-3+
% PD-1+ TIM-3+ LAG-3+
Degranulation
% CD107a+
IFNγ Production
% IFNγ+
NY-ESO-1 peptide concentration [nM]
e
PD-1 after Resting
PD-1 PE-Cy7
PD-1 GeoMean
d12
d19 - rested
f
Degranulation after Resting
IFNγ TNFα after Resting
% IFNγ TNFα positive
d13
d20 - rested
g
PD-1 blockade
MDA-MB-231 co-culture
% Specific Killing
10 µg/ml IgG4
10 µg/ml aPD-1
h
T ex expansion
fold expansion antigen-specific T cells
day 0
day 12 - T ex
i
Proliferation upon Restimulation
log2 fold change cell numbers restimulation vs rested
j
PD-1 - NA8
IFNγ TNFα – NA8
CD107a – NA8
% IFNγ+ TNFα+
T rest
T tumor NA8-Mel
T ex NA8-Mel

Supplementary Fig. 1

(a) Representative scheme of the transduction and stimulation procedure underlying the ex vivo T cell exhaustion model. Days are indicated as circles with different treatments in different colors and shadings. (b) Expression of TIM-3 on cells stimulated in the indicated conditions 12 days after the first stimulation. 1 way ANOVA with Holm-Sidak correction. n = 10 donors from n = 5 experiments except for Teff n = 4 of n = 2 experiments. (c) Exemplary gating strategy for single live CD8+ TCR-Vbeta13.1+ T cells. This gating strategy was used in Fig. 1b-g and Supplementary Fig. 1b,d-j. (d) Titration of NY-ESO-1 9V peptide dose at the four repetitive stimulations during the T_{ex} culture showing the percentage of PD-1+ TIM-3+ LAG-3+ cells (left), degranulation capacity measured by CD107a exposure over 4h (middle) and intracellular IFNγ production (right) in response to peptide-loaded T2s. T_{rest} and T_{tumor} conditions were performed as controls. n = 4 donors of n = 2 experimental replicates. Statistics are paired 1-way ANOVA with comparisons between T_{rest} and all other conditions with Dunnett's correction for multiple testing. (e) Representative plot of PD-1 expression and geometric mean signal of PD-1 after 13 days of culture in the indicated conditions followed by 6 days of resting in fresh medium and IL-2. n = 6 donors of n = 2 experimental replicates. (f) Degranulation capacity measured by CD107a exposure upon re-stimulation with T2 tumor cells pulsed with peptide. Measurements were performed before and after seven days of resting. n = 4 of n = 2 experimental replicates. (e-f) 2-way ANOVA with Holm-Sidak correction. (g) Specific killing of MDA-MB-231 tumor cells in a 4-day co-culture assay with Teff and Tex in presence of 100 nM NY-ESO-9V peptide. The co-culture was conducted in presence of either 10 μg/ml Nivolumab (anti-PD-1 blocking antibody) or hIgG4 (isotype control). Statistics are a paired 2-way ANOVA with Holm-Sidak correction. n = 5 donors. (h) Fold expansion of T_{ex} cells after the four rounds of stimulation compared to input on day 0. n = 5 donors. (i) Log2 fold change of cell numbers of the different conditions after re-stimulation with T2 tumor cells and peptide compared to expansion in IL2 alone within six days. Measured by flow cytometry on day 19 (six days post stimulation) using precision counting beads. 1-way ANOVA with Holm-Sidak correction. n = 6 donors of n = 2 experiments. (j) Expression of PD-1, co-production of IFNγ and TNFα and degranulation capacity of NY-ESO-1 specific T cells stimulated with NA8 melanoma cells for the indicated conditions. For degranulation and cytokines, the cells were re-stimulated for 5h with NY-ESO-9V loaded T2 cells in the presence of an anti-CD107a antibody and Monensin. 1-way ANOVA statistics with Holm-Sidak correction with n = 4 donors of n = 2 experiments. (b-j) Mean and SD are shown. * $p<0.05$, ** $p<0.01$, *** $p<0.001$, **** $p<0.0001$. Source data and exact p-values are provided as a Source Data file.

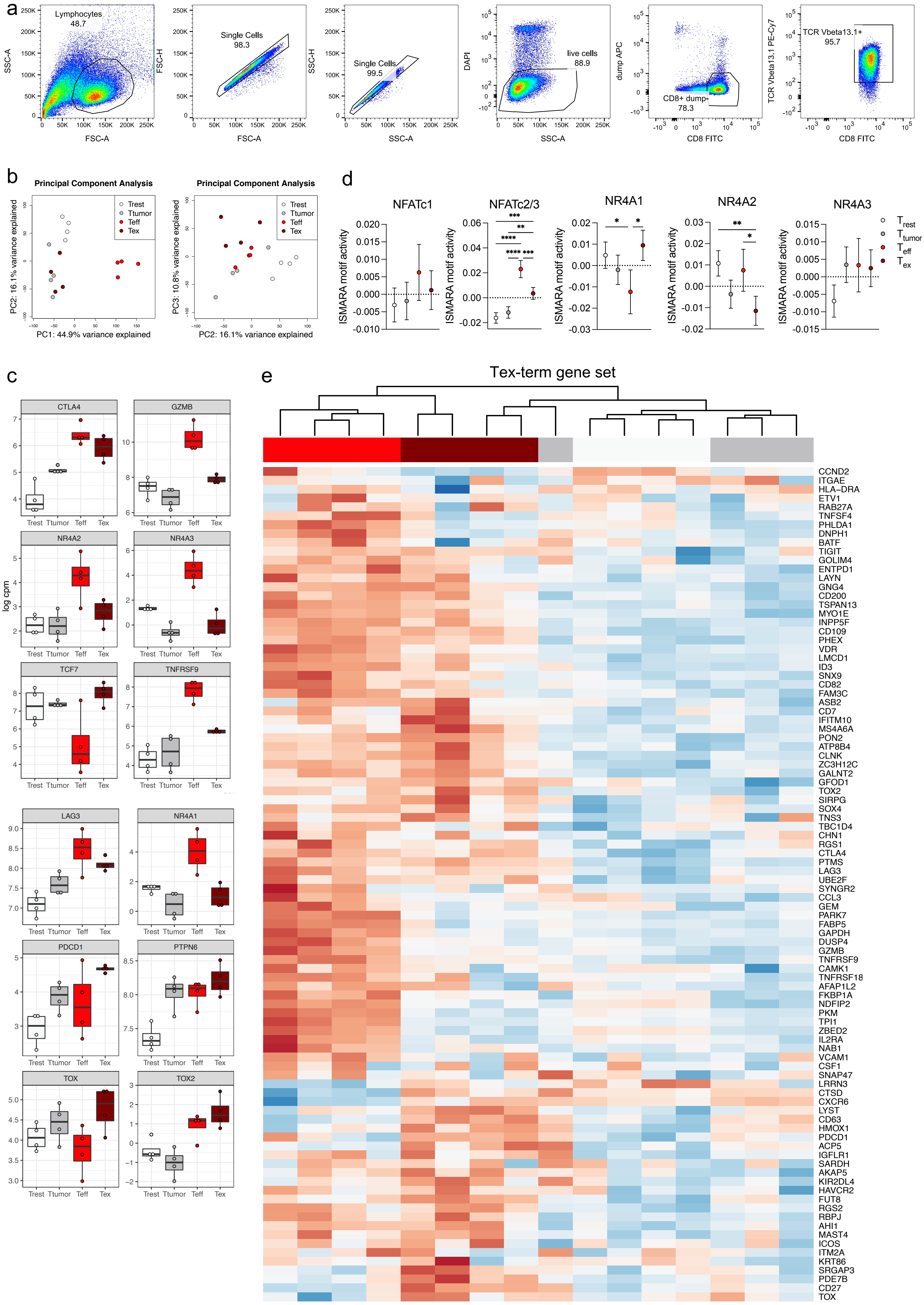
a
Lymphocytes
48.7
SSC-A
FSC-A
Single Cells
98.3
FSC-H
Single Cells
99.5
SSC-H
SSC-A
live cells
88.9
DAPI
dump APC
CD8+ dump-
78.3
CD8 FITC
TCR Vbeta13.1+
95.7
TCR Vbeta13.1 PE-Cy7
b
Principal Component Analysis
Trest
Ttumor
Teff
Tex
PC2: 16.1% variance explained
PC1: 44.9% variance explained
PC3: 10.8% variance explained
PC2: 16.1% variance explained
d
NFATc1
NFATc2/3
NR4A1
NR4A2
NR4A3
ISMARA motif activity
T_{rest}
T_{tumor}
T_{eff}
T_{ex}
c
CTLA4
GZMB
NR4A2
NR4A3
TCF7
TNFRSF9
LAG3
NR4A1
PDCD1
PTPN6
TOX
TOX2
log cpm
Trest Ttumor Teff Tex
e
Tex-term gene set
CCND2
ITGAE
HLA−DRA
ETV1
RAB27A
TNFSF4
PHLDA1
DNPH1
BATF
TIGIT
GOLIM4
ENTPD1
LAYN
GNG4
CD200
TSPAN13
MYO1E
INPP5F
CD109
PHEX
VDR
LMCD1
ID3
SNX9
CD82
FAM3C
ASB2
CD7
IFITM10
MS4A6A
PON2
ATP8B4
CLNK
ZC3H12C
GALNT2
GFOD1
TOX2
SIRPG
SOX4
TNS3
TBC1D4
CHN1
RGS1
CTLA4
PTMS
LAG3
UBE2F
SYNGR2
CCL3
GEM
PARK7
FABP5
GAPDH
DUSP4
GZMB
TNFRSF9
CAMK1
TNFRSF18
AFAP1L2
FKBP1A
NDFIP2
PKM
TPI1
ZBED2
IL2RA
NAB1
VCAM1
CSF1
SNAP47
LRRN3
CTSD
CXCR6
LYST
CD63
HMOX1
PDCD1
ACP5
IGFLR1
SARDH
AKAP5
KIR2DL4
HAVCR2
FUT8
RGS2
RBPJ
AHI1
MAST4
ICOS
ITM2A
KRT86
SRGAP3
PDE7B
CD27
TOX

Supplementary Fig. 2

(a) Exemplary sorting gating strategy for RNA sequencing samples. Live single CD8+ dump- TCR Vbeta13.1+ cells were sorted. Dump channel is a combination of anti-CD4 and anti-CD56 antibodies in APC. Used for Fig. 1i-j and Supplementary Fig. 2b-e. (b) Principal component analysis of individual replicates (n = 4 healthy donors) for the four investigated conditions. Axis indicate components and percentage of variance explained by these. (c) Boxplots (mean and interquartile ranges IQR 25% and 75% are shown as boxes, whiskers extend to outliers <1.5*IQR) of the log2 cpm expression of selected genes among the four conditions for the n = 4 donor replicates. (d) ISMARA transcription factor activity scores. Shown are the average and SD activity of 4 donor replicates. Statistics are 1-way ANOVAs with Holm-Sidak correction, only significant differences are shown. (e) Column clustered heatmap showing row-scaled log2-cpm expression of the top 100 genes of the Zheng et al.[1] Tex-term gene set. Colors indicate the comparisons as in k. * p<0.05, ** p <0.01, *** p < 0.001, **** p < 0.0001. Source data and exact p-values are provided as a Source Data file.

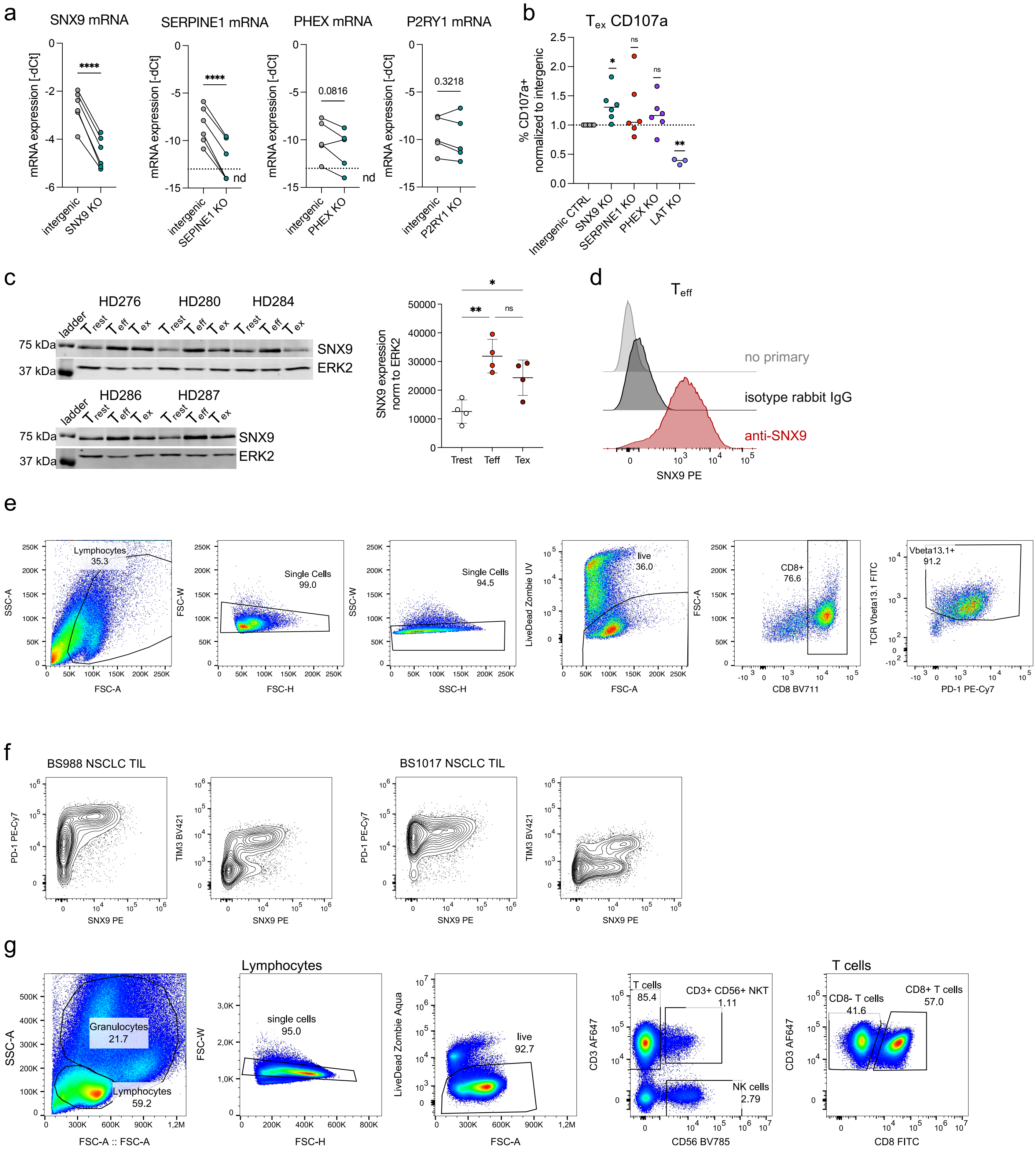
a
SNX9 mRNA
SERPINE1 mRNA
PHEX mRNA
P2RY1 mRNA
mRNA expression [-dCt]
****
0.0816
0.3218
nd
intergenic
SNX9 KO
SEPINE1 KO
PHEX KO
P2RY1 KO
b
Tex CD107a
% CD107a+ normalized to intergenic
ns
Intergenic CTRL
SNX9 KO
SERPINE1 KO
PHEX KO
LAT KO
c
HD276
HD280
HD284
HD286
HD287
ladder
T rest
T eff
T ex
75 kDa
37 kDa
SNX9
ERK2
SNX9 expression norm to ERK2
Trest
Teff
Tex
d
Teff
no primary
isotype rabbit IgG
anti-SNX9
SNX9 PE
e
Lymphocytes 35.3
Single Cells 99.0
Single Cells 94.5
live 36.0
CD8+ 76.6
Vbeta13.1+ 91.2
SSC-A
FSC-A
FSC-W
FSC-H
SSC-W
SSC-H
LiveDead Zombie UV
CD8 BV711
TCR Vbeta13.1 FITC
PD-1 PE-Cy7
f
BS988 NSCLC TIL
BS1017 NSCLC TIL
PD-1 PE-Cy7
TIM3 BV421
SNX9 PE
g
Lymphocytes
T cells
Granulocytes 21.7
Lymphocytes 59.2
single cells 95.0
live 92.7
T cells 85.4
CD3+ CD56+ NKT 1.11
NK cells 2.79
CD8- T cells 41.6
CD8+ T cells 57.0
SSC-A
FSC-A :: FSC-A
FSC-W
FSC-H
LiveDead Zombie Aqua
FSC-A
CD3 AF647
CD56 BV785
CD8 FITC

Supplementary Fig. 3

(a) mRNA expression of SNX9, SERPINE1 and PHEX in Teff with the indicated genes knocked out by Cas9-crRNA-tracrRNA electroporation. Shown are -dtCt values to the HPRT1 house keeping control gene. Statistics are paired t-tests. N = 6 donors of n = 2 experiments for SNX9 and SERPINE1. n = 5 of n = 2 experiments for P2RY1 and PHEX. (b) Percentage of CD107a+ cells after 4h of restimulation of Tex with the indicated KOs normalized to the intergenic control of the same donor (to account for donor-to-donor variation). Statistics are two-sided 1-sample t-tests against H_0 = 1. n = 6 donors of n = 2 experiments, except LAT = 3 of n = 1 experiment. (c) Western blot of SNX9 protein levels in T_{rest}, T_{eff} and T_{ex} conditions shown against ERK2 as a stable loading control for n = 5 different healthy donors. Protein standard ladders are shown. To the right, quantification of this blot using band densitometry in Fiji. Statistics are 1-way ANOVA with Holm-Sidak correction. (d) Fluorescence intensity (area) of SNX9 staining in T_{eff} measured by flow cytometry. Shown is a control which was not stained with the primary antibody (“no primary), an isotype control (rabbit IgG) and the rabbit-anti-SNX9 antibody. (e) Gating strategy to identify lymphocyte single live CD8+ TCR Vbeta13.1+ cells used in Fig. 2c and 3g-h. (f) Additional example plots showing PD-1 or TIM-3 versus SNX9 protein staining using flow cytometry of NSCLC infiltrating CD8 T cells. Shown as contour plots with 5% lines and including outliers. (g) Gating strategy to identify SNX9 expression in lymphocyte single live CD3+ CD56- CD8+ CD4- used in Fig. 2d-e and Supplementary Fig. 3f. * $p<0.05$, ** $p<0.01$, *** $p<0.001$, **** $p<0.0001$. Source data and exact p-values are provided as a Source Data file.

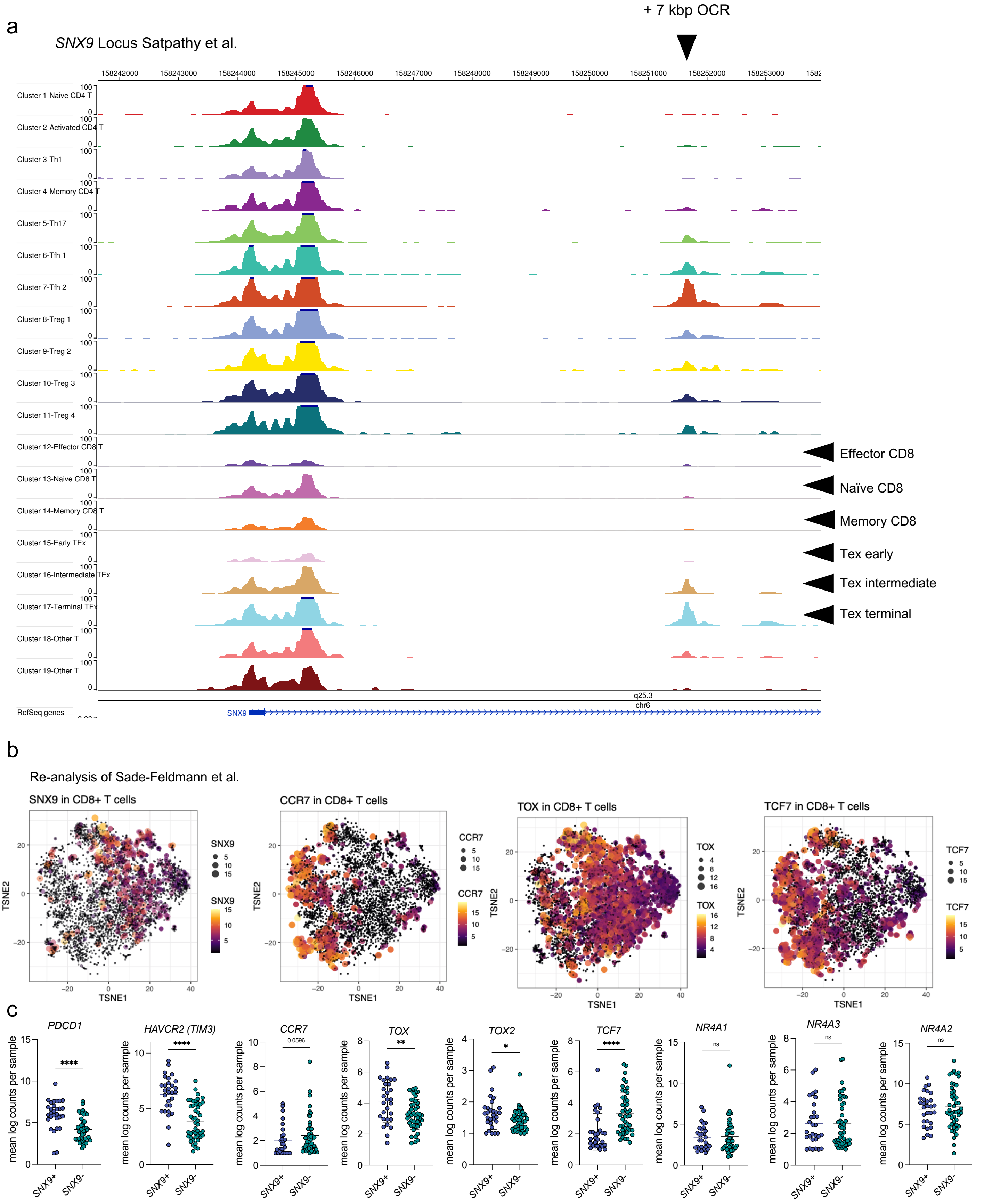

a
+ 7 kbp OCR
SNX9 Locus Satpathy et al.
158242000
158243000
158244000
158245000
158246000
158247000
158248000
158249000
158250000
158251000
158252000
158253000
Cluster 1-Naive CD4 T
Cluster 2-Activated CD4 T
Cluster 3-Th1
Cluster 4-Memory CD4 T
Cluster 5-Th17
Cluster 6-Tfh 1
Cluster 7-Tfh 2
Cluster 8-Treg 1
Cluster 9-Treg 2
Cluster 10-Treg 3
Cluster 11-Treg 4
Cluster 12-Effector CD8 T
Cluster 13-Naive CD8 T
Cluster 14-Memory CD8 T
Cluster 15-Early TEx
Cluster 16-Intermediate TEx
Cluster 17-Terminal TEx
Cluster 18-Other T
Cluster 19-Other T
Effector CD8
Naïve CD8
Memory CD8
Tex early
Tex intermediate
Tex terminal
q25.3
chr6
RefSeq genes
SNX9
b
Re-analysis of Sade-Feldmann et al.
SNX9 in CD8+ T cells
CCR7 in CD8+ T cells
TOX in CD8+ T cells
TCF7 in CD8+ T cells
TSNE1
TSNE2
SNX9
CCR7
TOX
TCF7
c
PDCD1
HAVCR2 (TIM3)
CCR7
TOX
TOX2
TCF7
NR4A1
NR4A3
NR4A2
mean log counts per sample
****
****
0.0596
**
*
****
ns
ns
ns
SNX9+
SNX9-

Supplementary Fig. 4

(a) Single-cell ATAC seq tracks displaying open chromatin regions from BCC TILs of Satpathy et al.[2] are displayed using the WashU Epigenome Browser. The human SNX9 locus is shown. (RefSeq annotation of hg19) with the transcriptional start site found around the prominent OCR region to the left. An arrow indicates the OCR specific to exhaustion, Treg and Tfh specific at approx. +7 kbp of the TSS. (b) CD8 T cells extracted from the published human melanoma TIL scRNAseq data set by Sade-Feldman et al.[3] are shown in a TSNE plot. Expression of SNX9, TCF7, and TOX are indicated by the size and color of the dots. n = 5138 cells. (c) Mean log normalized counts and SD for CCR7, TCF7, TOX, and TOX2 in SNX9+ versus SNX9- CD8+ T cells. Expression of PDCD1, HAVCR2, CCR7, TOX, TOX2, TCF7, NR4A1, NR4A2 and NR4A3 in the scRNAseq data set by Sade-Feldman et al. Mean log counts of CD8 T cells for the two subsets (SNX9- and SNX9+, defined by > 1 log count) are shown per sample. Samples with less than ten cells in either population were excluded. n = 47 for SNX9- and n = 29 for SNX9+, representing individual patient samples split into these two categories. Statistics are Mann-Whitney tests (non-normal distribution). Mean and SD are indicated. * $p<0.05$, ** $p <0.01$, *** $p < 0.001$, **** $p < 0.0001$. Source data and exact p-values are provided as a Source Data file.

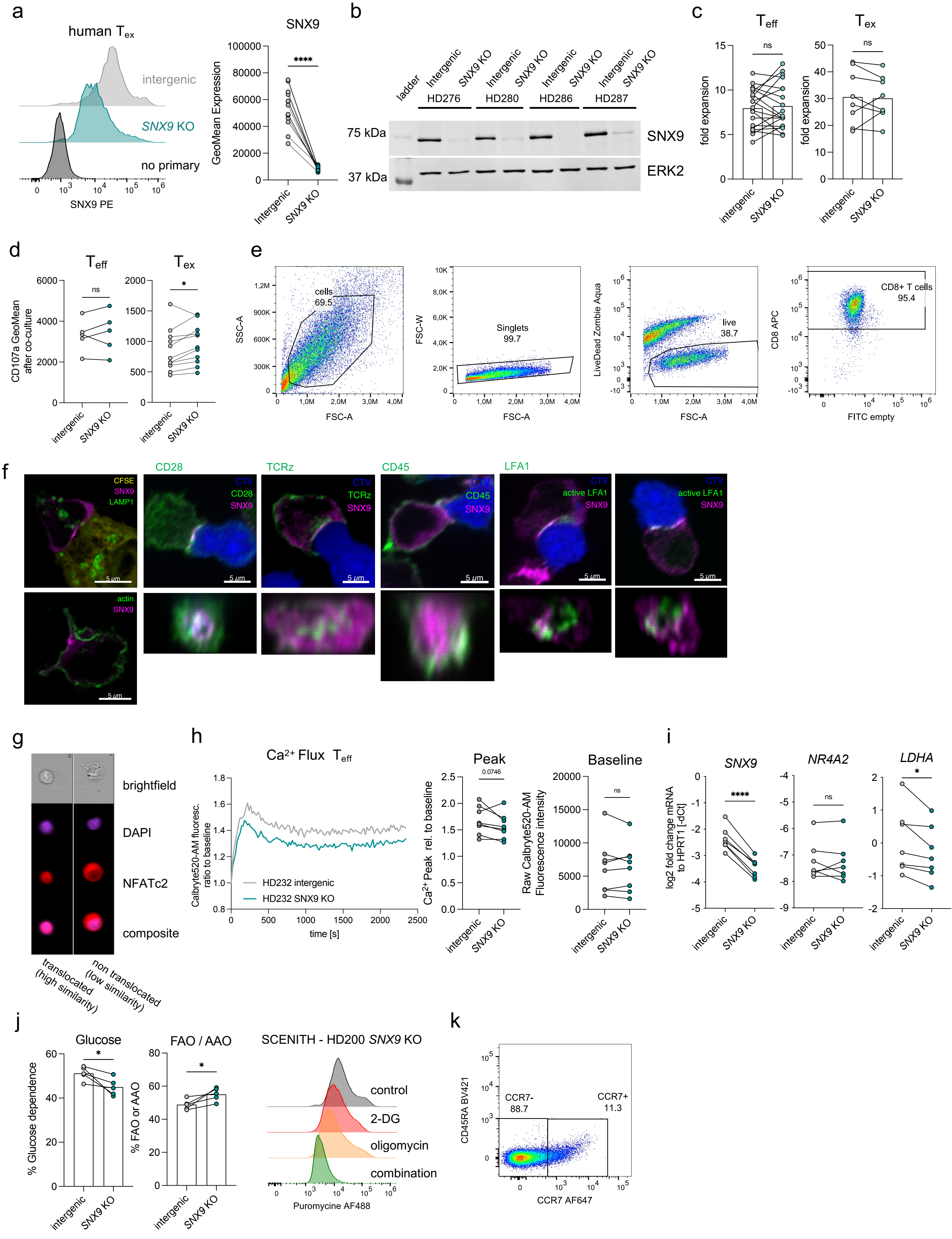
a
human Tex
intergenic
SNX9 KO
no primary
SNX9 PE
SNX9
GeoMean Expression
****
Intergenic
SNX9 KO
b
ladder
Intergenic
SNX9 KO
HD276
HD280
HD286
HD287
75 kDa
37 kDa
SNX9
ERK2
c
Teff
Tex
fold expansion
ns
intergenic
SNX9 KO
d
CD107a GeoMean after co-culture
ns
*
e
cells 69.5
Singlets 99.7
live 38.7
CD8+ T cells 95.4
SSC-A
FSC-A
FSC-W
LiveDead Zombie Aqua
CD8 APC
FITC empty
f
CD28
TCRz
CD45
LFA1
CFSE
SNX9
LAMP1
CTV
active LFA1
actin
g
brightfield
DAPI
NFATc2
composite
translocated (high similarity)
non translocated (low similarity)
h
Ca2+ Flux Teff
Calbryte520-AM fluoresc. ratio to baseline
HD232 intergenic
HD232 SNX9 KO
time [s]
Peak
0.0746
Ca2+ Peak rel. to baseline
Baseline
Raw Calbryte520-AM Fluorescence intensity
i
SNX9
NR4A2
LDHA
log2 fold change mRNA to HPRT1 [-dCt]
j
Glucose
% Glucose dependence
FAO / AAO
% FAO or AAO
SCENITH - HD200 SNX9 KO
control
2-DG
oligomycin
combination
Puromycine AF488
k
CCR7- 88.7
CCR7+ 11.3
CD45RA BV421
CCR7 AF647

Supplementary Fig. 5

(a) Example flow cytometry histogram and quantification of donor replicates of SNX9 expression in T_{ex} with or without Cas9-RNP electroporation at the beginning of the procedure. Statistics is paired two-sided paired t-test. (b) Westernblot for protein expression of SNX9 and ERK2 as a loading control in Teff of four donors with or without SNX9 KO. The same antibody was used as for the flow cytometry experiments. (c) Fold cell expansion for T_{eff} (left, n = 19) and T_{ex} (right, n = 8) from the first day of stimulation. Data are shown on a log2 scale. Donor replicates are shown as connected dots for intergenic and SNX9 KO conditions. Statistics are paired t-tests. (d) Degranulation capacity for T_{eff} (left, n = 5) and T_{ex} (right, n = 11) after re-stimulation with T2 + peptide shown as delta unstimulated geometric mean fluorescence intensity of n = 4 experiments. Statistics are paired two-sided t-tests. (e) Exemplary gating strategy to identify lymphocyte single live CD8+ cells used in Supplementary Fig. 5d,i, 6d, and 10e. (f) Additional single-z slices of spinning disk confocal images for NY-ESO-1 specific cells co-incubated with NY-ESO-9V peptide-pulsed T2 cells for 30min. In the actin and LAMP1 images, tumor cells were stained with CFSE. All images show anti-SNX9 staining in magenta. On the right T_{eff} are shown with electroporated CD28-EGFP or TCR-EGFP, or antibody-based staining of anti-CD45 and LFA1. “En face” images are 0.25 μm sections of the synapse region looking into the direction of the T cell from a 3D representation rendered in Imaris 9. All images are found in the source data files for Fig 3c. Actin (n = 6), and LAMP1/Perforin (n = 3), CD28-EGFP (n = 6), TCRz-EGFP (n = 5), anti-CD45 (n = 6) and LFA1 (n = 4). (g) Example images of the NFATc2 nuclear translocation readout using an Imagestream MK-II. Shown are an example of a cell that shows non-translocated and translocated NFATc2 signal towards the nucleus (in DAPI). (h) Calcium flux peak normalized to baseline of n = 8 donors of n = 3 experiments. Shown in connected dot plots are the maximum peak intensities normalized to baseline and the raw baseline values for the Calbryte520-AM signal (before beads addition). Statistics are paired two-sided t-tests. (i) SNX9, NR4A2 and LDHA mRNA quantification by qPCR of T_{ex} at day 6 of culture. Shown are -dtCt values to the housekeeping control (HPRT1). Statistics are paired two-sided t-tests. n = 7 donors of n = 2 experiments. (j) Glucose dependence and FAO/AAO values from the SCENITH flow cytometry single cell metabolism assay of n = 5 donors of n = 2 experiments. On the right, representative flow cytometry histograms for anti-puromycine-AF488 for the four conditions used to calculate the glucose dependence vs FAO/AAO are shown [93]. Statistics are two-sided paired t-tests. (k) Exemplary gating strategy to identify CCR7+ cells in addition to gating shown in Supplementary Fig. 3e. Source data and exact p-values are provided as a Source Data file.

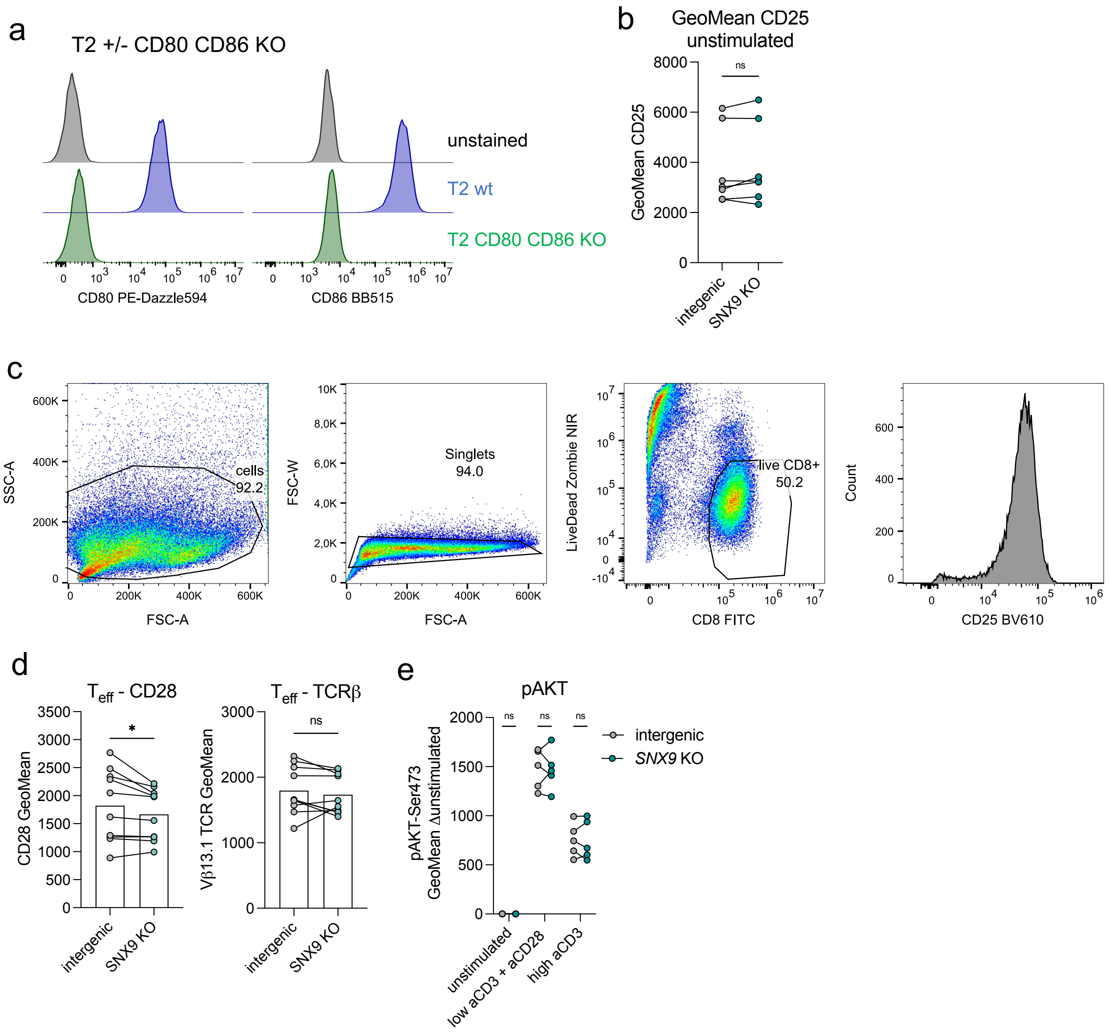

Supplementary Fig. 6

(a) Histograms of flow cytometric determination of CD80/86 levels on T2 wt and T2 CD80 CD86 cell lines. (b) Geometric mean intensity of CD25 signal measured by flow cytometry for the unstimulated controls in the antibody titration assays. n = 6 healthy donors of n = 2 independent experiments. Statistics is a two-sided paired t-test. (c) Exemplary gating strategy to identify single live CD8+ cells used in Fig. 3i-k and Supplementary Fig. 6b and 6e. (d) Geometric mean intensities of CD28 and TCRβ13.1 (variant of the NY-ESO-1 specific TCR) measured by flow cytometry on the surface of T_{eff}. Statistics are paired two-sided t-tests. n = 10 healthy donors of n = 2 independent experiments. (e) Geometric mean area of fluorescence for anti-p-AKT-Ser473 shown as delta unstimulated controls measured by flow cytometry. T_{eff} with or without *SNX9* KO were stimulated for 30 min with plate-bound antibody (low CD3 + CD28 = 1.25μg/ml OKT3 + 2.5 μg/ml CD28.2, high CD3 = 5 μg/ml OKT3). Statistics is a paired-2-way ANOVA with Holm-Sidak correction. n = 5 healthy donors. * p<0.05, ** p <0.01, *** p < 0.001, **** p < 0.0001. Source data and exact p-values are provided as a Source Data file.

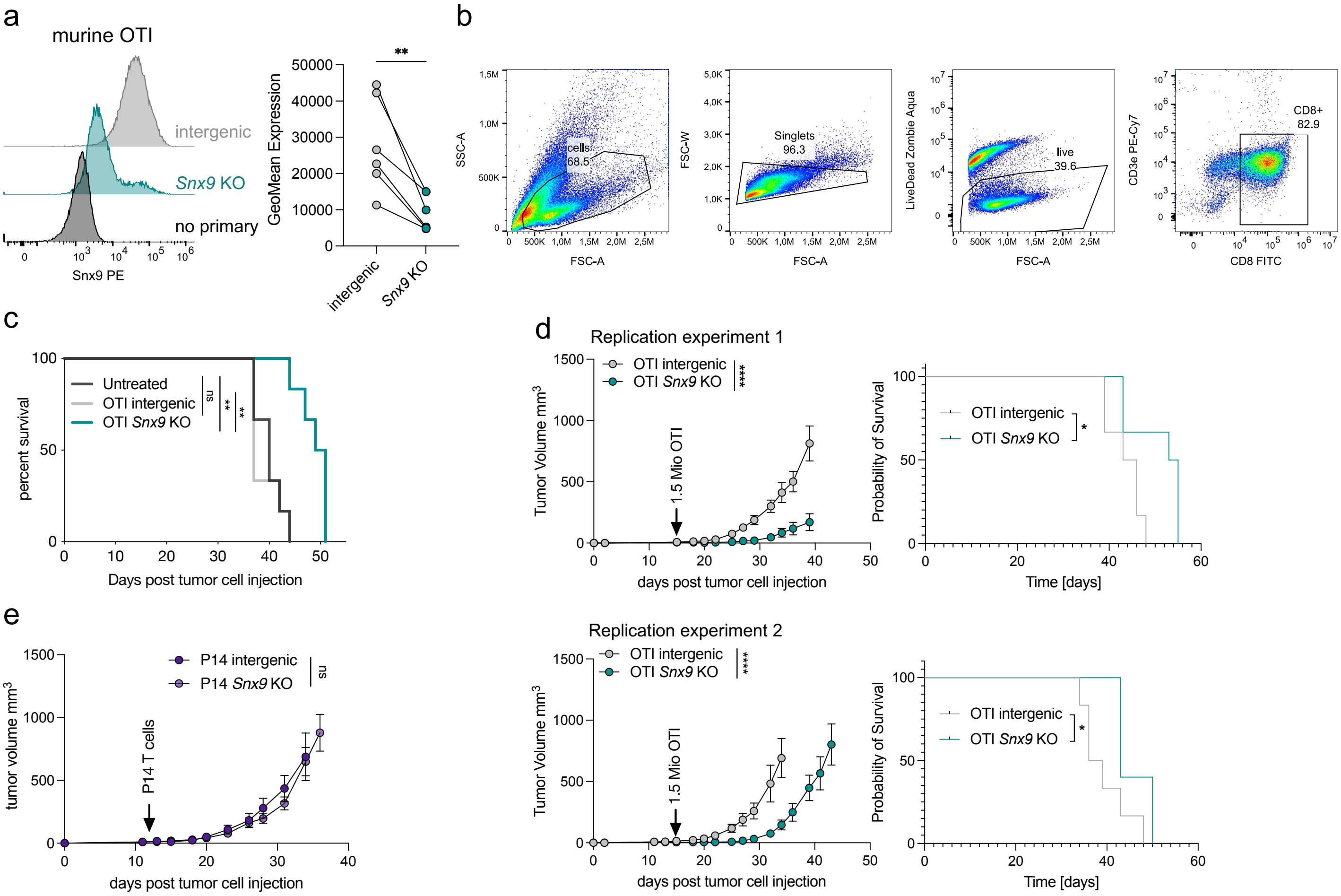

Supplementary Fig. 7

(a) Example flow cytometry histogram of Snx9 staining in murine OTI cells for the indicated conditions and quantification thereof (n = 6 experiments) for *Snx9* KO and intergenic. Statistics is a paired two-sided t-test. (b) Exemplary gating strategy to identify live single CD8+ OTI cells used in Supplementary Fig. 7a (c) Survival curve of C57BL/6 mice with MC38-OVA tumors with adoptive transfer of OTI T cells with or without *Snx9* KO at day 13 post tumor injection. n= 6 mice per condition and statistics are Bonferroni-adjusted Mantel-Cox log-rank tests. (d) Additional replications of tumor growth measurements and survival of MC38-OVA tumors in C57BL/6 mice with transfer of intergenic and Snx9 KO OTI transfer. N = 6 mice per condition. N = 3 experiments in total. Statistics for growth curves are pairwise 2-way ANOVA followed Bonferroni correction. Statistics for survival curves are Mantel-Cox log rank tests. (e) Tumor growth curve in mm^3 (mean and SEM) of MC38-OVA bearing tumors in C57BL/6 mice with a transfer of P14 (LCMV gp33 specific T cells) with and without a *Snx9* KO for n = 6 mice per condition. Statistic is a 2-way ANOVA. * p<0.05, ** p <0.01, *** p < 0.001, **** p < 0.0001. Source data and exact p-values are provided as a Source Data file.

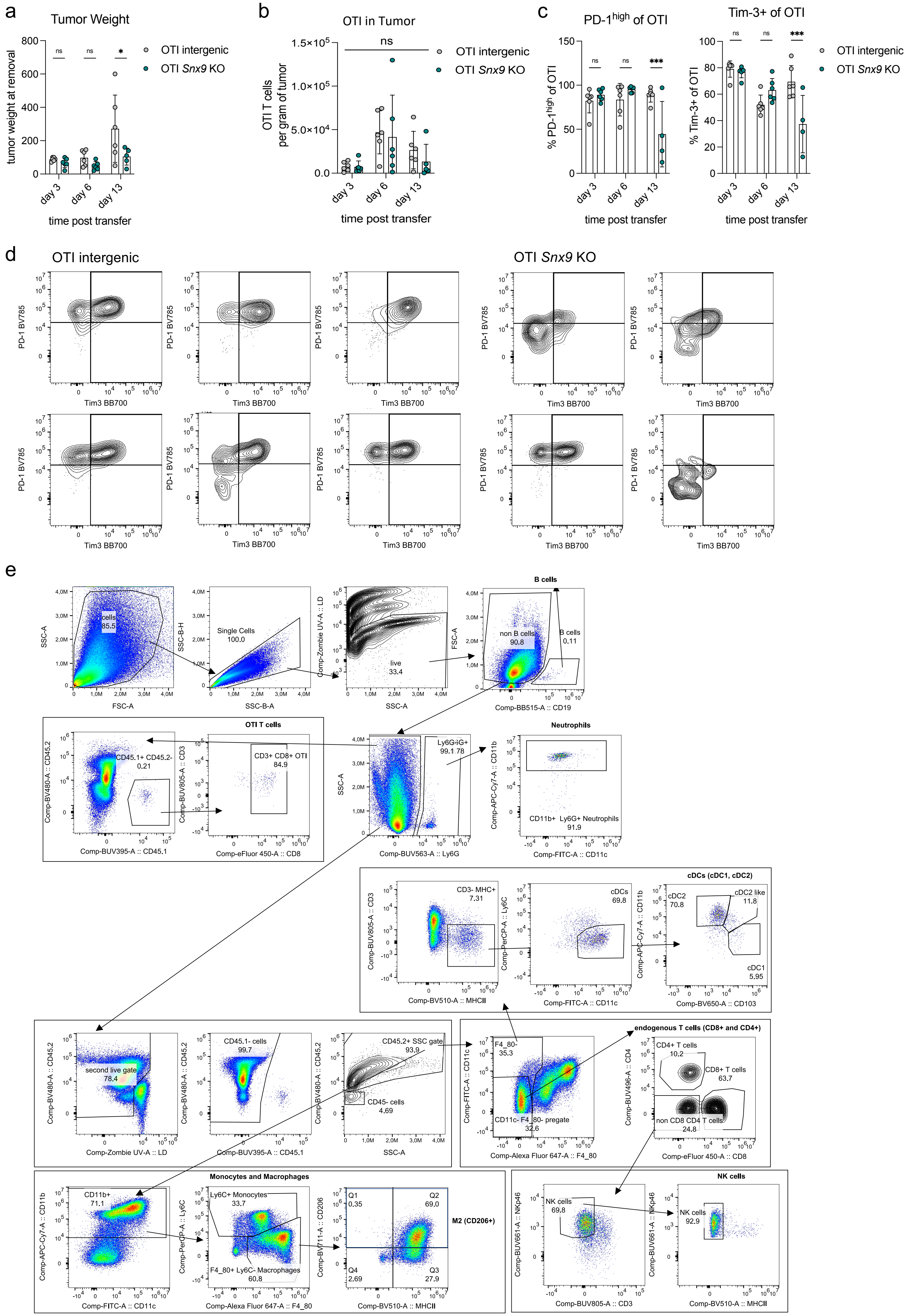

a
Tumor Weight
tumor weight at removal
800
600
400
200
0
ns
ns
*
OTI intergenic
OTI Snx9 KO
day 3
day 6
day 13
time post transfer
b
OTI in Tumor
OTI T cells per gram of tumor
1.5×10^5
1.0×10^5
5.0×10^4
0.0
ns
OTI intergenic
OTI Snx9 KO
day 3
day 6
day 13
time post transfer
c
PD-1^high of OTI
% PD-1^high of OTI
150
100
50
0
ns
ns
***
day 3
day 6
day 13
time post transfer
Tim-3+ of OTI
% Tim-3+ of OTI
100
80
60
40
20
0
ns
ns
***
OTI intergenic
OTI Snx9 KO
day 3
day 6
day 13
time post transfer
d
OTI intergenic
OTI Snx9 KO
PD-1 BV785
Tim3 BB700
e
B cells
SSC-A
FSC-A
cells 85.5
SSC-B-H
SSC-B-A
Single Cells 100.0
Comp-Zombie UV-A :: LD
live 33.4
FSC-A
non B cells 90.8
B cells 0.11
Comp-BB515-A :: CD19
OTI T cells
Comp-BV480-A :: CD45.2
CD45.1+ CD45.2- 0.21
Comp-BUV395-A :: CD45.1
Comp-BUV805-A :: CD3
CD3+ CD8+ OTI 84.9
Comp-eFluor 450-A :: CD8
Ly6G+G+ 99.1 78
Comp-BUV563-A :: Ly6G
Neutrophils
Comp-APC-Cy7-A :: CD11b
CD11b+ Ly6G+ Neutrophils 91.9
Comp-FITC-A :: CD11c
cDCs (cDC1, cDC2)
Comp-BUV805-A :: CD3
CD3- MHC+ 7.31
Comp-BV510-A :: MHCII
Comp-PerCP-A :: Ly6C
cDCs 69.8
Comp-FITC-A :: CD11c
Comp-APC-Cy7-A :: CD11b
cDC2 70.8
cDC2 like 11.8
cDC1 5.95
Comp-BV650-A :: CD103
second live gate 78.4
Comp-Zombie UV-A :: LD
CD45.1- cells 99.7
Comp-BUV395-A :: CD45.1
CD45.2+ SSC gate 93.9
CD45- cells 4.69
SSC-A
Comp-FITC-A :: CD11c
F4_80- 35.3
CD11c- F4_80- pregate 32.6
Comp-Alexa Fluor 647-A :: F4_80
endogenous T cells (CD8+ and CD4+)
Comp-BUV496-A :: CD4
CD4+ T cells 10.2
CD8+ T cells 63.7
non CD8 CD4 T cells 24.8
Comp-eFluor 450-A :: CD8
Monocytes and Macrophages
Comp-APC-Cy7-A :: CD11b
CD11b+ 71.1
Comp-FITC-A :: CD11c
Comp-PerCP-A :: Ly6C
Ly6C+ Monocytes 33.7
F4_80+ Ly6C- Macrophages 60.8
Comp-Alexa Fluor 647-A :: F4_80
Comp-BV711-A :: CD206
Q1 0,35
Q2 69,0
Q3 27,9
Q4 2,69
Comp-BV510-A :: MHCII
M2 (CD206+)
NK cells
Comp-BUV661-A :: NKp46
NK cells 69.8
Comp-BUV805-A :: CD3
NK cells 92.9
Comp-BV510-A :: MHCII

Supplementary Fig. 8

(a) Tumor weight of MC38-OVA tumors at timepoints for the flow cytometry analysis of intratumoral OTI cells. Statistics are 2-way ANOVA with Holm-Sidak correction. n = 6 mice per condition. (b) Numbers of adoptively transferred OTI T cells found in the tumor (identified by CD45.1, adjusted by Precision counting beads) and normalized per gram of tumor weight. n = 6 (for *Snx9* KO at d13 n = 5 due to ulceration). (c) Percentage of PD-1^{high} and Tim-3+ OTI cells at different timepoints post transfer. (d) Flow cytometry plots showing PD-1 versus TIM-3 for the samples on day 13 post OTI transfer shown in h. (e) Gating strategies to identify OTI T cells and other endogenous immune cell types among intratumoral cells of MC38-OVA tumors. Detailed gating strategy also described in Material and Methods. Relates to data shown in Fig. 4h, Supplementary Fig. 8a-d and 9e. (a-c) Mean and SD are shown, statistics are 2-way ANOVA with Holm-Sidak correction. * p<0.05, ** p <0.01, *** p < 0.001, **** p < 0.0001. Source data and exact p-values are provided as a Source Data file.

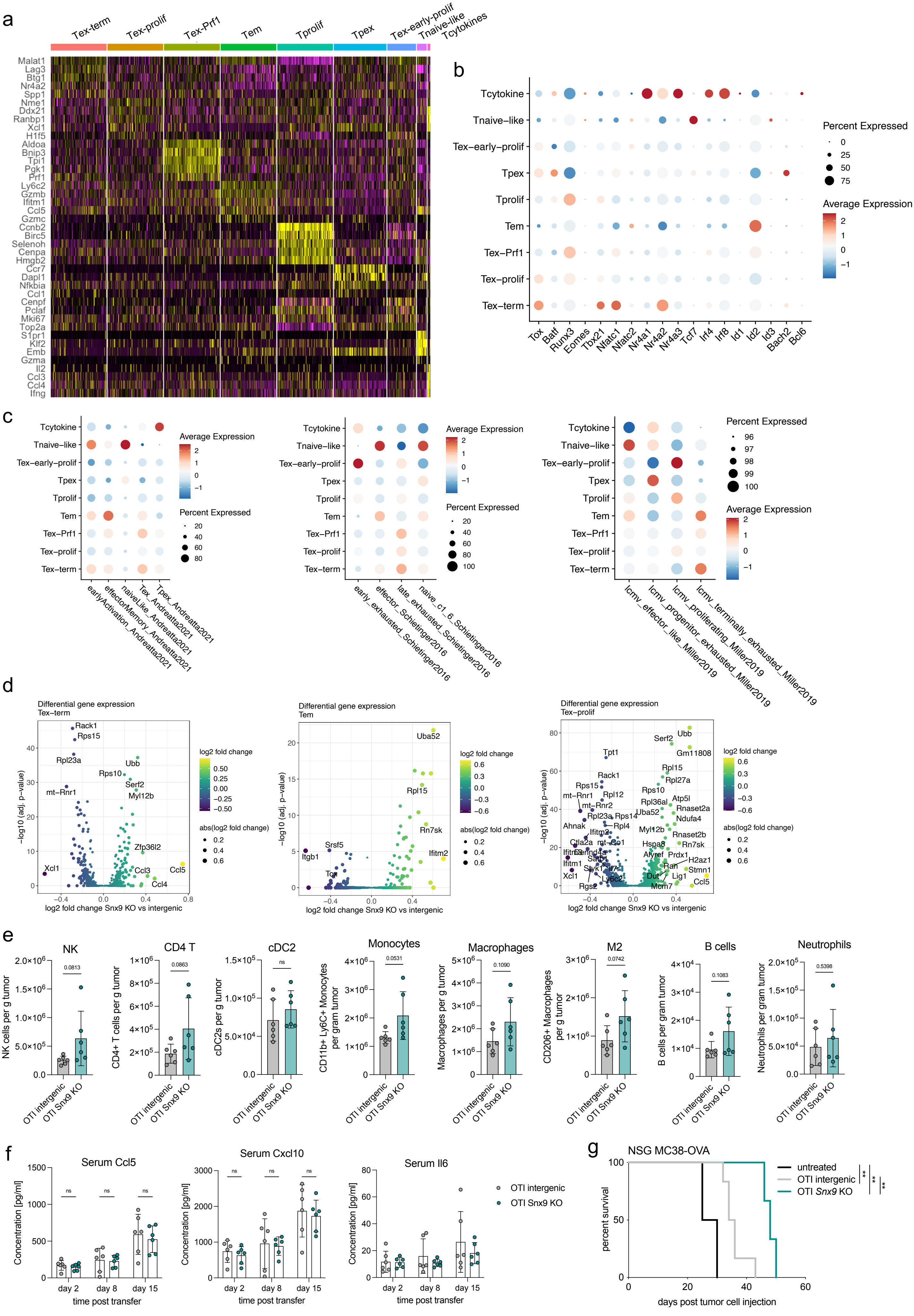
a
Tex-term
Tex-prolif
Tex-Prf1
Tem
Tprolif
Tpex
Tex-early-prolif
Tnaive-like
Tcytokines
Malat1
Lag3
Btg1
Nr4a2
Spp1
Nme1
Ddx21
Ranbp1
Xcl1
H1f5
Aldoa
Bnip3
Tpi1
Pgk1
Prf1
Ly6c2
Gzmb
Ifitm1
Ccl5
Gzmc
Ccnb2
Birc5
Selenoh
Cenpa
Hmgb2
Ccr7
Dapl1
Nfkbia
Ccl1
Cenpf
Pclaf
Mki67
Top2a
S1pr1
Klf2
Emb
Gzma
Il2
Ccl3
Ccl4
Ifng
b
Tcytokine
Tnaive−like
Tex−early−prolif
Tpex
Tprolif
Tem
Tex−Prf1
Tex−prolif
Tex−term
Tox
Batf
Runx3
Eomes
Tbx21
Nfatc1
Nfatc2
Nr4a1
Nr4a2
Nr4a3
Tcf7
Irf4
Irf8
Id1
Id2
Id3
Bach2
Bcl6
Percent Expressed
0
25
50
75
Average Expression
2
1
0
−1
c
Average Expression
Percent Expressed
20
40
60
80
earlyActivation_Andreatta2021
effectorMemory_Andreatta2021
naiveLike_Andreatta2021
Tex_Andreatta2021
Tpex_Andreatta2021
100
early_exhausted_Schietinger2016
effector_Schietinger2016
late_exhausted_Schietinger2016
naive_c1_6_Schietinger2016
96
97
98
99
100
lcmv_effector_like_Miller2019
lcmv_progenitor_exhausted_Miller2019
lcmv_proliferating_Miller2019
lcmv_terminally_exhausted_Miller2019
d
Differential gene expression
Tex-term
Rack1
Rps15
Rpl23a
Ubb
Rps10
Serf2
mt−Rnr1
Myl12b
Zfp36l2
Xcl1
Ccl3
Ccl5
Ccl4
−log10 (adj. p−value)
log2 fold change Snx9 KO vs intergenic
log2 fold change
0.50
0.25
0.00
−0.25
−0.50
abs(log2 fold change)
0.2
0.4
0.6
Differential gene expression
Tem
Uba52
Rpl15
Rn7sk
Srsf5
Itgb1
Ifitm2
Tox
Differential gene expression
Tex−prolif
Ubb
Serf2
Gm11808
Tpt1
Rack1
Rpl15
Rps15
Rpl27a
Rps10
mt−Rnr1
Rpl12
Atp5l
mt−Rnr2
Rpl36al
Rnaset2a
Rpl23a
Rps14
Uba52
Ndufa4
Ahnak
Rpl4
Ifitm2
Myl12b
Rnaset2b
Cd52
mt−Co1
Hspa8
Rn7sk
Ifitm3
Dennd4a
Alyref
Prdx1
Satb1
H2az1
Ifitm1
Styk1
Il7r
Ran
Stmn1
Xcl1
Ly6c2
Dut
Lig1
Ccl5
Rgs2
Mcm7
e
NK
CD4 T
cDC2
Monocytes
Macrophages
M2
B cells
Neutrophils
0.0813
0.0863
ns
0.0531
0.1090
0.0742
0.1083
0.5398
NK cells per g tumor
CD4+ T cells per g tumor
cDC2s per g tumor
CD11b+ Ly6C+ Monocytes per gram tumor
Macrophages per g tumor
CD206+ Macrophages per g tumor
B cells per gram tumor
Neutrophils per gram tumor
OTI intergenic
OTI Snx9 KO
f
Serum Ccl5
Serum Cxcl10
Serum Il6
Concentration [pg/ml]
ns
day 2
day 8
day 15
time post transfer
OTI intergenic
OTI Snx9 KO
g
NSG MC38-OVA
percent survival
days post tumor cell injection
untreated
OTI intergenic
OTI *Snx9* KO
**
**
**

Supplementary Fig. 9

(a - d) Data from the scRNAseq dataset of OTI cells 13 days post transfer in MC38-OVA tumors. (a) Heatmap showing row-scaled expression of the indicated marker genes defined by FindMarkers in Seurat for the clusters shown in columns. Yellow indicates high expression, magenta low expression. Cells were downsampled before plotting. (b) Average expression as color and percentage expression as size of the indicated transcription factors and nuclear genes with known involvement in T cell differentiation for each cluster. (c) Average expression as color and percentage detection as size for the indicated gene sets for each cluster. (d) Differentially expressed genes between *Snx9* KO and intergenic OTI cells for the indicated clusters: Tex-term, Tem and Tex-prolif. Log2 fold change is indicated as x-axis, color and size of the dots, while the y-axis represents to -log10 adjusted p-value. (e) Numbers of the indicated immune cell subsets in MC38-OVA tumors of C57BL/6 mice three days post OTI transfer with or without Snx9 KO. Numbers were corrected by Precision counting beads and normalized to the tumor weight. n = 6 mice. Statistics are unpaired two-sided t-tests. (f) Serum cytokines in MC38-OVA bearing C57BL/6 mice at the indicated times post OTI transfer with or without Snx9 KO. n = 6 mice per condition. Statistics are unpaired 2-way ANOVA with Holm-Sidak correction. (g) Survival curve (humane endpoints) for NSG mice with MC38-OVA tumors with a transfer of OTI T cells 12 days post tumor injection. n= 6 mice per condition and statistics are Bonferroni-adjusted Mantel-Cox log-rank tests. (e-f) Mean and SD are shown. * $p<0.05$, ** $p <0.01$, *** $p < 0.001$, **** $p < 0.0001$. Source data and exact p-values are provided as a Source Data file.

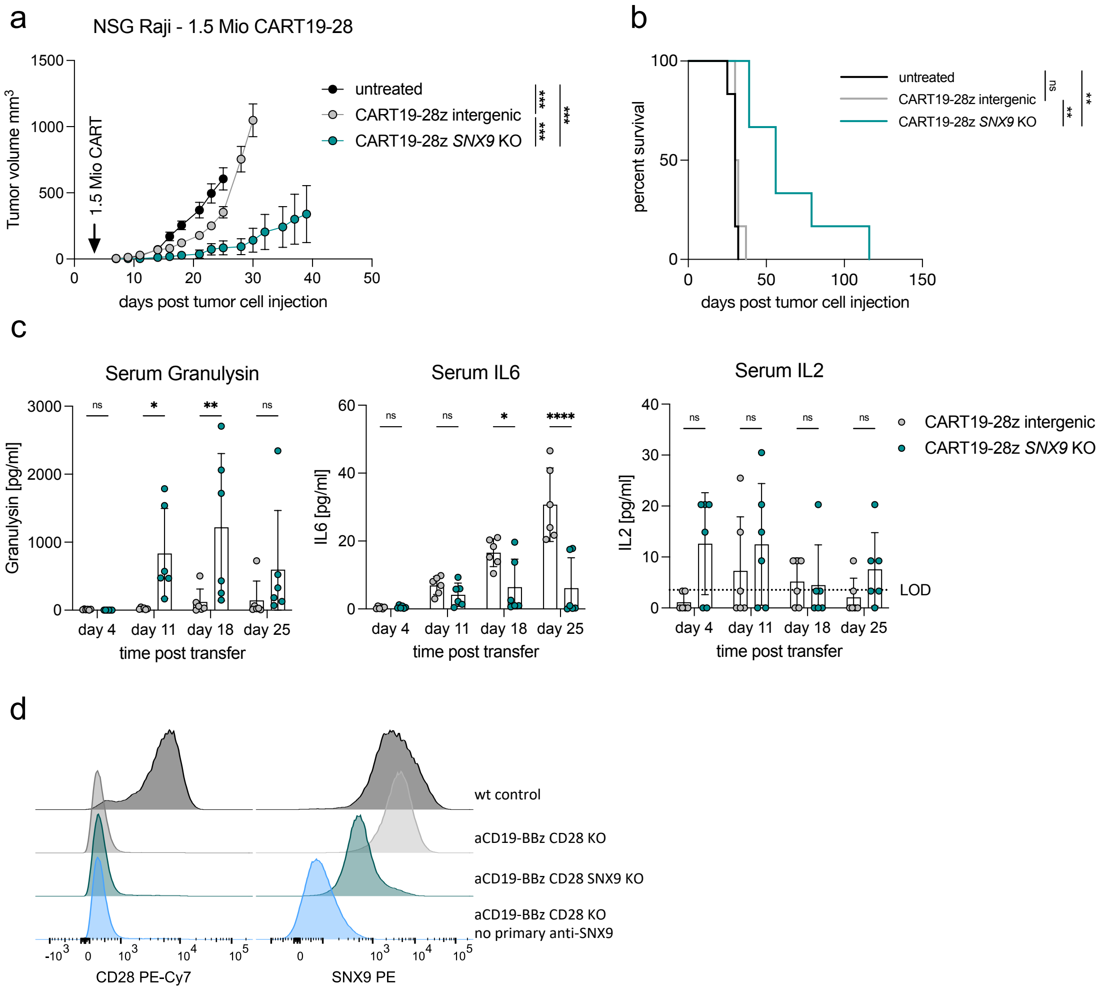

Supplementary Fig. 10

(a) Tumor volumes in mm³ for subcutaneous Raji tumors in NSG mice either untreated or with 1.5 Mio anti-CD19-28z CARs with or without *SNX9* KO. Statistics are individually performed 2-way ANOVAs with Bonferroni correction. n = 6 mice per condition. Shown are the mean and SEM. (b) Survival of these mice (humane endpoints) with Bonferroni-adjusted Mantel-Cox log-rank tests. n = 6 mice per condition. (c) Legendplex-based measurement of the indicated human proteins in sera of Raji-bearing NSG mice with the indicated CAR treatments. The limit of detection (LOD) is indicated for IL2. Statistics are paired-2-way ANOVAs with Holm-Sidak correction. n = 6 mice per condition. Mean and SD are shown. (d) Histograms for fluorescence intensity of CD28 on the left and SNX9 on the right for the indicated conditions measured by flow cytometry for anti-CD19-BBz CAR T cells. A control without the primary anti-SNX9 is included. * p<0.05, ** p <0.01, *** p < 0.001, **** p < 0.0001. Source data and exact p-values are provided as a Source Data file.

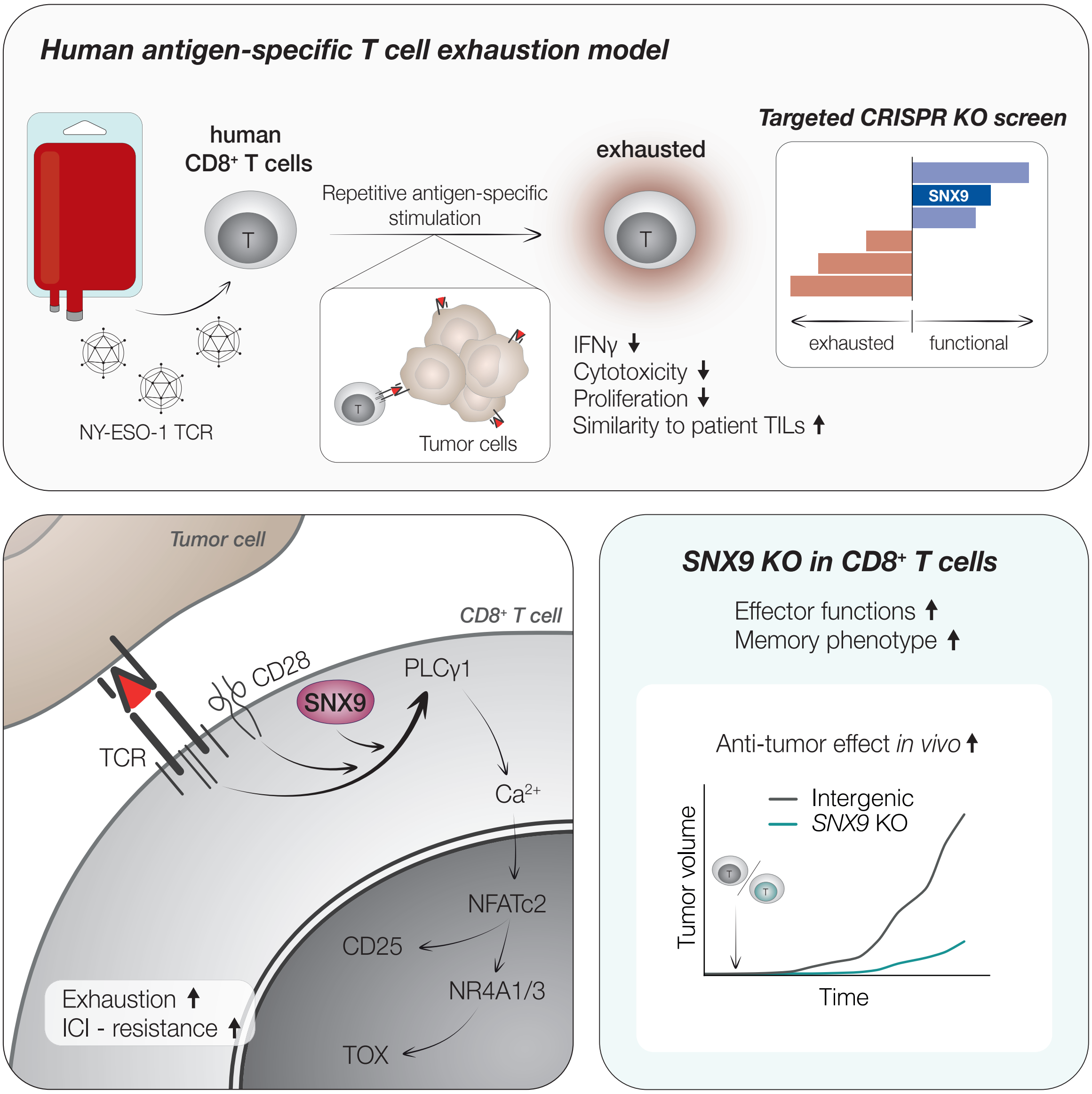

Supplementary Fig. 11

Graphical summary of the key findings. Top panel shows the generation of the human *ex vivo* model for T cell exhaustion and the pooled targeted CRISPR-Cas9 screen. The lower left panel shows the proposed mechanism how SNX9 amplifies TCR/CD28 signaling towards PLCγ1, Ca^{2+}, NFATc2, NR4A1/3, and TOX. The lower right panel shows the effects of *SNX9* KO *in vivo.*

Supplementary Table 1: Reagents and Resources

Name	Source	Identifier
**Critical reagents**		
NY-ESO-1 peptide 9V: SLLMWITQV >95% purity	EZ Biolabs	custom
CD8 microbeads human	Miltenyi	130-045-201
Anti-mouse microbeads	Miltenyi	130-048-402
T cell stimulation and expansion kit	Miltenyi	130-091-441
Polyethylenimine > 25kDa	Poly Sciences Inc.	23966-1
Nucleospin plasmid miniprep	Machery Nagel	740588.250
NucleoBond Xtra Midi Kit	Machery Nagel	740410.50
NucleoBond Xtra Maxi Kit	Machery Nagel	740414.50
Precision Counting Beads	Biolegend	424902
ALT-R crRNA	Integrated DNA Technologies (IDT)	Sequence specific, see Supplementary Table 3
ALT-R tracrRNA	Integrated DNA Technologies (IDT)	1072533
3Alt-R Cas9 Electroporation enhancer	Integrated DNA Technologies (IDT)	10007805
16 % para-formaldehyde	Electron Microscopy Services	15710-S
Vectashield Mounting Media Vibrance	Reactolab SA	H-1700-10
5 well microscopy slides	Hendly-Essex	PH299 B230615
Retronectin	Takara	T100B
NucleoSpin Gel and PCR Clean-up	Machery Nagel	740609.50
QIAamp DNA Blood Mini Kit	Qiagen	51104
Polybrene	Sigma	TR-1004-G
PluronicF127 20%	Thermo Fisher	P3000MP
Foxp3 / Transcription Factor Fixation/Permeabilization Concentrate and Diluent	eBioscience	00-5521-00
IC Fixation Buffer	eBioscience	00-8222-49
Poly-L-Lysine	Santa Cruz Biotechnology	sc-286689

PrimeTime Gene Expression Mastermix	IDT	230524087
Human IL2 (Proleukin, Aldesleukin)	Clinigen	N.a.
Legendplex MurineVirusResponse	Biolegend	740621
Legendplex Human CD8/NK	Biolegend	741065
**Plasmids**		
NY-ESO-1 TCR lentiviral vector, codon optimized, pairing optimized: pRRL 131 (WT) T2A 1xATG Cys	Kindly provided by Dr. Michael Hebeisen and Dr. Natalie Rufer[4,5]	Upon request to Natalie Rufer (University of Lausanne)
LentiCRISPRv2-mCherry	Addgene,<br>was as a gift from Agata Smogorzewska	RRID:Addgene_99154
pcDNA3.1(+)/Luc2=tdT	Addgene,<br>was a gift from Christopher Contag	RRID:Addgene_32904
Anti-CD19-CD28z-T2A-copGFP	Was a gift from Wolfgang Schamel	Upon request to Wolfgang Schamel (University of Freiburg)
p-CMV-IE-human-TCRzeta-EGFP	Was a gift from Jérémie Rossy, University of Konstanz	Upon request to Jérémie Rossy (University of Konstanz)
p-CMV-IE-human-CD28-EGFP	Was a gift from Jérémie Rossy, University of Konstanz	Upon request to Jérémie Rossy (University of Konstanz)
pLV-EFS-FMC63-BBz-P2A-mCherry	Was a gift from Gregor Hutter, University of Basel	Upon request to Gregor Hutter, University of Basel
**Biological Samples**		
Human Peripheral Blood Buffy Coat	Blood Donation Center Basel, Switzerland	NA
Human non-small cell lung cancer	University Hospital Basel, Switzerland	NA

Human AB+ male serum	Blood Donation Center Basel, Switzerland	NA
Fetal Bovine Serum	Pan Biotech	P30-5500
Panexin Basic, Defined Components (FBS replacement)	Pan Biotech	P04-96950
**Dyes**		
Zombie UV	Biolegend	423107
Zombie Acqua	Biolegend	423102
Zombie NIR	Biolegend	423106
Fixable Viability Dye eF450	ThermoFisher	65-0863-14
Cell Trace Violet	ThermoFisher	C34557
Cell Trace CFSE	ThermoFisher	C34554
Phalloidin-AF647	ThermoFisher	A22287
Calbryte520-AM	AAT Bioquest	20650
**Software and algorithms**		Version or source
PinAPL-py	Spahn et al.[6]	http://pinapl-py.ucsd.edu/ accessed February 2019
Huygens Deconvolution	Scientific Volume Imaging https://svi.nl/HomePage	Huygens Remote Manager 3.6.0-3-g0891e1e
OMERO	openmicroscopy.org University of Dundee	OMERO.web 5.4.10-ice36-b105
R Studio Version	https://rstudio.com	2022.07.1 Build 554
Graphpad Prism	Graphpad Software LLC	v9.3.0
R	https://rstudio.com	v4.2.1
Flow Jo	Becton Dickinson & Company	v10.8.1
Imaris	Bitplane, Oxford Instruments	v9
NIS Elements	Nikon	v5.21.03
FACS Diva Software	BD	v8.0.1
CytExpert	Beckmann Coulter	v2.4.0.28
SpectroFlow	CyTek	v3.0.1

IDEAS	Luminex Amnis	v6.3
Excel for Mac	Microsoft	v16.6.27
Inspire	Luminex Amnis	v200.1.620.0
**Real-time qPCR PrimeTime™ Probes**		
**Transcript**	**Manufacturer**	**Ordering Number**
SNX9	IDT	*Hs.PT.58.21424684*
TOX	IDT	*Hs.PT.58.28002606*
TOX2	IDT	*Hs.PT.58.39787291*
NR4A1	IDT	*Hs.PT.58.39997829*
NR4A2	IDT	*Hs.PT.58.704850*
NR4A3	IDT	*Hs.PT.58.14945655*
HPRT1	IDT	*Hs.PT.58.v.45621572*
LDHA	IDT	*Hs.PT.40245343*
SERPINE1	IDT	*Hs.PT.58.3938488.g*
PHEX	IDT	*Hs.PT.58.26760508*
P2RY1	IDT	*Hs.PT.58.24915313.g*

Supplementary Table 2 Antibodies

Antibody target	Target species	Fluorochrome	Provider	Catalog Nr.	Clone	Dilution
CD3	Human	PE-CF594	BD	562280	UCHT1	100
CCR7	Human	Alexa Fluor 647	Biolegend	3532218	G043H7	50
CD3	Human	APC-eF780	eBioscience	47-0038-42	SK7	100
CD11b	Human	APC	Biolegend	301310	ICRF44	100
CD4	Human	APC	eBioscience	17-0047-42	SK3	100
CD56	Human	APC	Miltenyi	130-113-312	REA196	100
CD45	Human	APC-H7	BD	560178	2D1	100
CD56	Human	BV785	BioLegend	362550	5.1H11	100
CD8	Human	FITC	eBioscience	11-0087	SK1	100
CD8	Human	BV605	Biolegend	344742	RPA-T8	50
CD8	Human	APC	Biolegend	344722	SK1	100
CD8	Human	BV711	Biolegend	344734	SK1	100
EOMES	Human	PerCP eFluor710	eBioscience	46-4877	WD1928	50
TNFα	Human	APC	eBioscience	17-7349	MAb11	20
IFNγ	Human	BV421	BD	564791	4S.B3	20
KI67	Human	APC	BioLegend	350514	Ki-67	50
LAMP-1 (CD107a)	Human	PE	BD	555801	H4A3	100
PD-1	Human	PE-Cy7	BD	561272	EH12.1	20
TBET	Human	BV421	BioLegend	644815	4B10	50
TIM-3	Human	BV605	BioLegend	345018	F38-2E2	50
TIM-3	Human	BV421	BioLegend	345008	F38-2E2	50
TIM-3	Human	PE	BioLegend	345006	F38-2E2	50
TCR Vbeta13.1	Human	FITC	Biolegend	362404	H131	20
TCR Vbeta13.1	Human	PE-Cy7	Biolegend	362406	H131	20
TCF7	Human	AF647	Biolegend	655203	7F11A10	20
CD28	Human	FITC	Biolegend	302906	CD28.2	50

CD28	Human	PE-Cy7	eBioscience	25-0289.42	CD28.2	50
CD57	Human	BV421	BD	563896	NK-1	100
LAG-3	Human	APC	eBioscience	17-2239-41	3DS223H	50
TOX	Human	PE	Invitrogen	12-6502-82	TXRX10	50
GZMB	Human	FITC	Biolegend	372206	QA16A02	100
KI67	Human	BV421	BD	562899	B56	50
CD45RA	Human	BV421	Biolegend	304130	HI100	100
CD4	Human	BV605	Biolegend	317438	OKT4	100
CD14	Human	BV605	Biolegend	301834	M5E2	100
CD19	Human	BV605	Biolegend	363024	SJ25C1	100
SNX9	Human	None	ThermoFisher	PA5-56734	polyclonal	500
CD8α	Human	AF488	R&D	37006	FAB1509G	100
LAMP1 (CD107a) Microscopy	Human	none	Biolegend	328602	H4A3	100
Perforin (Microscopy)	Human	none	BD Pharmingen	556434	δG9	100
CD11a/CD18 (active LFA-1) (Microscopy)	Human	none	Biolegend	363402	M24	100
Actin	Human	none	Sigma Aldrich	A3853-200UL	AC-40	200
NFATc2	Human	none	Cell Signaling Technologies	#5861	D43B1	800
CD45	Human	AF532	eBioscience	58-0459-42	HI30	50
CD25	Human	BV605	Biolegend	302632	BC96	100
CD69	Human	BV421	Biolegend	310930	FN50	100
CD80	Human	PE/Dazzle 594	Biolegend	305230	2D10	50
CD86	Human	BB515	BD Bioscience	564545	2331 (FUN-1)	100

PD-1	Human	None	Bristol-Meyers-Squibb SA	Opdivo	Nivolumab, clinical grade	10 μg/ml
CTLA-4	Human	None	Bristol-Meyers-Squibb SA	Yervoy	Ipilimumab, clinical grade	10 μg/ml
pAKT (phospho-Ser473)	Human	None	ThermoFisher	700392	98H9L8 rabbit	500
pPLCγ1 (phospho-Tyr783)	Human	None	Cell Signaling Technologies	14008S	D6M9S	450
Goat-anti-mouse IgG	Mouse	AF488	ThermoFisher	A32723	Polyclonal, cross absorbed	500
Goat-anti-mouse IgG	Mouse	AF568	ThermoFisher	A-11031	Polyclonal, cross absorbed	200
Goat-anti-rabbit IgG	Rabbit	AF647	ThermoFisher	A21246	Polyclonal, cross absorbed	400
Goat-anti-rabbit IgG	Rabbit	AF488	ThermoFisher	A11034	Polyclonal, cross absorbed	500
Goat-anti-rabbit IgG	Rabbit	PE	ThermoFisher	A10542	Polyclonal, cross absorbed	500
None	Rabbit	None	Jackson	011-000-003	Polyclonal IgG isotype control	0.1 μg/ml
Ultra-LEAF Purified anti-human CD3	Mouse	None	Biolegend	317326	OKT3	As indicated in figure legends
Ultra-LEAF	Mouse	None	Biolegend	302934	CD28.2	As indicated

Purified anti-human CD28						in figure legends
CD45.1	Mouse	BUV395	BD Biosciences	565212	A20	100
CD4	Mouse	BUV496	BD Biosciences	612952	GK1.5	100
Ly-6G	Mouse	BUV563	BD Biosciences	612921	1A8	200
NKp46	Mouse	BUV661	BD Biosciences	741678	29A14	70
CD3	Mouse	BUV805	BD Biosciences	741895	145-2C11	70
PD-L1	Mouse	BV421	Biolegend	124315	10F.9G2	150
CD8	Mouse	eFluor 450	Thermo Fisher Scientific	eBio 48-0081-82	53-6.7	100
CD45.2	Mouse	BV480	BD Biosciences	566077	104	200
MHCII	Mouse	BV510	Biolegend	107636	M5/114.15.2	300
CD80	Mouse	BV605	Biolegend	104792	16-10A1	70
CD103	Mouse	BV650	BD Biosciences	748256	2.00E+07	70
CD206	Mouse	BV711	Biolegend	141727	C068C2	100
PD-1	Mouse	BV785	Biolegend	135225	29F.1A12	100
CD19	Mouse	BB515	BD Biosciences	564509	1D3	100
CD11c	Mouse	FITC	Biolegend	117306	N418	100
Ki67	Mouse	AF532	Thermo Fisher Scientific	58-5698-82	SolA15	200
Ly-6C	Mouse	PerCP	Biolegend	128028	HK1.4	200
Tim-3	Mouse	BB700	BD Biosciences	747619	5D12/TIM-3	70

GzmB	Mouse	PE-eFluor610	Thermo Fisher Scientific	61-8898-82	NGZB	100
CD25	Mouse	PE-Cy5.5	Thermo Fisher Scientific	35-0251-82	PC61.5	100
CD28	Mouse	APC	Biolegend	102110	37.51	100
F4/80	Mouse	AF647	Biolegend	123122	BM8	100
TCF-7	Mouse	AF700	R&D Systems	FAB8224N	# 812145	
CD11b	Mouse	APC-Cy7	Biolegend	101226	M1/70	150
CD45	Mouse	V450	BD	560501	30-F11	100
CD19	Mouse	FITC	BD	553785	1D3	100
Chicken IgY	goat	AF488	Invitrogen	A-11039	Polyclonal affinity purified	500
GFP	Chicken	None	Abcam	Ab13970	polyclonal	1000

Supplementary Table 3. CRISPR-Cas9 Guide Sequences

**Guide identifier**	**gene**	**Species**	**Guide RNA sequence (in crRNA)**
sgIntergenic19822	Intergenic region control	Homo sapiens	GAGAGGGTGGCGACAGAGCG
sgSNX9-1 (Sabatini library)	*SNX9*	Homo sapiens	GAAACATCAAAGGAGAACGA
sgSNX9-2 (Hs.Cas9.SNX9.1.AL)	*SNX9*	Homo sapiens	GAGGTAGGATAAACCCACAT
sgPHEX_4	*PHEX*	Homo sapiens	GGGCTGATGTCTTTCAGATG
sgSERPINE1_3	*SERPINE*	Homo sapiens	GCTCCTTGTACAGATGCCGG
sgP2RY1_4 (Hs.Cas9.P2RY1.1.AW)	*P2RY1*	Homo sapiens	GCTGGTGTGGCTCATTGTGG
sgLAT_1	*LAT*	Homo sapiens	GCAGGAGCCCCAGCACGCAG
sgCD28	CD28	Homo sapiens	CTATAGCTTGCTAGTAACAG
sgCD80 (Hs.Cas9.CD80.1.AA)	CD80	Homo sapiens	CGCTTTCAAGCGGGAACACC
sgCD86 (Hs.Cas9.CD86.1.AB)	CD86	Homo sapiens	GGGCCGCACAAGTTTTGATT
sgINTERGENIC_27270	Intergenic region control	Mus musculus	GCACAGCACAAGCAGACCTA
sgSnx9_9	*Snx9*	Mus musculus	CCAGCGGGGAAACAGTCGTG
Mm.Cas9.SNX9.1.AF	*Snx9*	Mus musculus	AGGTAGGATAAACCCACATA

Supplementary References

1. Zheng, L. *et al.* Pan-cancer single-cell landscape of tumor-infiltrating T cells. *Science (80-.).* **374**, (2021).
2. Satpathy, A. T. *et al.* Massively parallel single-cell chromatin landscapes of human immune cell development and intratumoral T cell exhaustion. *Nat. Biotechnol.* **37**, 925–936 (2019).
3. Sade-Feldman, M. *et al.* Defining T Cell States Associated with Response to Checkpoint Immunotherapy in Melanoma. *Cell* **175**, 998-1013.e20 (2018).
4. Schmid, D. A. et al. Evidence for a TCR Affinity Threshold Delimiting Maximal CD8 T Cell Function. J. Immunol. 184, 4936–4946 (2010).
5. Hebeisen, M. et al. Molecular insights for optimizing T cell receptor specificity against cancer. Front. Immunol. 4, 1–10 (2013).
6. Spahn, P. N. et al. PinAPL-Py: A comprehensive web-application for the analysis of CRISPR/Cas9 screens. Sci. Rep. 7, 15854 (2017).
