# Supplementary material for: Deletion of SNX9 alleviates CD8 T cell exhaustion for effective cellular cancer immunotherapy
Source: Nat Commun. 2023 Feb 2;14:86. doi: 10.1038/s41467-022-35583-w (PMC9895440; doi:10.1038/s41467-022-35583-w)
Supplement: Supplementary file 3 — Description of Additional Supplementary Files [file 41467_2022_35583_MOESM3_ESM.pdf]

Title: Supplementary Data 1:
Description: RNA sequencing of human T cells for the four conditions of the ex vivo exhaustion model. Scaled centered cpms and the cluster id for all the genes found in Figure 1i.

Title: Supplementary Data 2:
Description: Differential gene expression analyses between the indicated conditions (sheets) of the RNA sequencing of human T cells from the Tex model. Relates to Figure 1i. The statistics are derived from edgeR as described in the methods and can be recreated using the deposited code.

Title: Supplementary Data 3
Description: Gene set enrichment analysis (sheet: Gene Set Enrichment) relating to Figure 1j. The other sheets contain the gene sets used for the analyses (top 100 Genes by Effect Size ranking derived from Zheng et al. Science 2021)

Title: Supplementary Data 4
Description: Genes differentially expressed among the four conditions of the Tex model. Statistics are derived from edgeR. The genes were overlapped with published datasets describing T cell exhaustion and ranked according to the degree of overlap. Second sheet 'gene sets' contains listed EntrezIDs for human gene sets used for overlap analyses

Title: Supplementary Data 5
Description: Supplementary Information relating to single cell RNA sequencing of intratumoral OTI T cells isolated on day 13 post transfer to MC38-OVA bearing C57BL/6 mice.
