# Supplementary material for: Deletion of SNX9 alleviates CD8 T cell exhaustion for effective cellular cancer immunotherapy
Source: Nat Commun. 2023 Feb 2;14:86. doi: 10.1038/s41467-022-35583-w (PMC9895440; doi:10.1038/s41467-022-35583-w)
Supplement: Supplementary file 9 — Reporting Summary [file 41467_2022_35583_MOESM9_ESM.pdf]

nature portfolio

Corresponding author(s): Marcel P. Trefny and Alfred Zippelius

Last updated by author(s): Nov 25, 2022

Reporting Summary

Nature Portfolio wishes to improve the reproducibility of the work that we publish. This form provides structure for consistency and transparency in reporting. For further information on Nature Portfolio policies, see our Editorial Policies and the Editorial Policy Checklist.

Statistics

For all statistical analyses, confirm that the following items are present in the figure legend, table legend, main text, or Methods section.

n/a	Confirmed	
☐	☒	The exact sample size (*n*) for each experimental group/condition, given as a discrete number and unit of measurement
☐	☒	A statement on whether measurements were taken from distinct samples or whether the same sample was measured repeatedly
☐	☒	The statistical test(s) used AND whether they are one- or two-sided<br>*Only common tests should be described solely by name; describe more complex techniques in the Methods section.*
☒	☐	A description of all covariates tested
☐	☒	A description of any assumptions or corrections, such as tests of normality and adjustment for multiple comparisons
☐	☒	A full description of the statistical parameters including central tendency (e.g. means) or other basic estimates (e.g. regression coefficient) AND variation (e.g. standard deviation) or associated estimates of uncertainty (e.g. confidence intervals)
☐	☒	For null hypothesis testing, the test statistic (e.g. *F*, *t*, *r*) with confidence intervals, effect sizes, degrees of freedom and *P* value noted<br>*Give P values as exact values whenever suitable.*
☒	☐	For Bayesian analysis, information on the choice of priors and Markov chain Monte Carlo settings
☒	☐	For hierarchical and complex designs, identification of the appropriate level for tests and full reporting of outcomes
☒	☐	Estimates of effect sizes (e.g. Cohen's *d*, Pearson's *r*), indicating how they were calculated

Our web collection on statistics for biologists contains articles on many of the points above.

Software and code

Policy information about availability of computer code

Data collection	FACS Diva Software BD v8.0.1<br>CytExpert Beckmann Coulter v2.4.0.28<br>SpectroFlow CyTek v3.0.1<br>NIS Elements Nikon v5.21.03<br>Luminex Amnis v200.1.620.0
Data analysis	The following software was used for analysis:<br>PinAPL-py http://pinapl-py.ucsd.edu/ accessed February 2019<br>Huygens Deconvolution Scientific Volume Imaging https://svi.nl/HomePage Huygens Remote Manager 3.6.0-3-g0891e1e<br>OMERO openmicroscopy.org University of Dundee OMERO.web 5.4.10-ice36-b105<br><br>R Studio Version https://rstudio.com 2022.07.1 Build 554<br>Graphpad Prism Graphpad Software LLC v9.3.0<br>R https://rstudio.com v4.2.1<br>Flow Jo Becton Dickinson & Company v10.8.1<br>Imaris Bitplane, Oxford Instruments v9<br>NIS Elements Nikon v5.21.03<br>FACS Diva BD v8.0.1<br>CytExpert Beckmann Coulter v2.4.0.28<br>IDEAS Luminex Amnis v6.3<br>SpectroFlow CyTek v3.0.1<br>Excel for Mac Microsoft v16.6.27

Inspire Luminex Amnis v200.1.620.0

Sequencing data (RNAseq, CRISPR screen and scRNAseq) data were analyzed in R, using Bioconductor packages, specifically:
edgeR_3.38.4 (depending on limma_3.52.2)
Seurat_4.1.1
ggplot2_3.3.6
SingleCellExperiment_1.18.0
TxDb.Hsapiens.UCSC.hg38.knownGene_3.15.0
org.Hs.eg.db_3.15.0
ComplexHeatmap_2.4.3
scater_1.24.0
viridis_0.6.2
future_1.27.0
dittoSeq_1.8.1
biomaRT_2.52.0
ggrepel_0.9.1
tidyr_1.2.0
dplyr_1.0.9

For manuscripts utilizing custom algorithms or software that are central to the research but not yet described in published literature, software must be made available to editors and reviewers. We strongly encourage code deposition in a community repository (e.g. GitHub). See the Nature Portfolio guidelines for submitting code & software for further information.

Data

Policy information about availability of data

All manuscripts must include a data availability statement. This statement should provide the following information, where applicable:

- Accession codes, unique identifiers, or web links for publicly available datasets
- A description of any restrictions on data availability
- For clinical datasets or third party data, please ensure that the statement adheres to our policy

The data for this manuscript has been deposited as a super series to the Gene Expression Omnibus (GEO) under the accession number GSE190247 [https://www.ncbi.nlm.nih.gov/geo/query/acc.cgi?acc=GSE190247]. Within this superseries, processed data for the bulk RNA sequencing of the Tex model are available under accession code GSE210534 [https://www.ncbi.nlm.nih.gov/geo/query/acc.cgi?acc=GSE210534], raw and processed data from the CRISPR-Cas9 screen gRNA sequencing under accession code GSE190246 [https://www.ncbi.nlm.nih.gov/geo/query/acc.cgi?acc=GSE190246], and raw and processed data for single cell sequencing of intratumoral OTI T cells with or without Snx9 KO under accession code GSE210535 [https://www.ncbi.nlm.nih.gov/geo/query/acc.cgi?acc=GSE210535]. For the bulk RNA sequencing of the Tex model, raw sequencing FASTQ files are available through the European Genome-Phenome Archive under accession number EGAS00001006794 [https://ega-archive.org/studies/EGAS00001006794].
The Sade-Feldman et al. publicly available data used in this study are available in the GEO database under accession code GSE120575 [https://www.ncbi.nlm.nih.gov/geo/query/acc.cgi?acc=GSE120575]. The Satpathy et al. publicly available single cell ATACseq data used in this study are available on the WashU EpiGenome Browser under http://epigenomegateway.wustl.edu/legacy/?genome=hg19&session=7UZG0iF90b&statusId=807471043. The remaining data are available within the Article, Supplementary Information or Source Data file.

Field-specific reporting

Please select the one below that is the best fit for your research. If you are not sure, read the appropriate sections before making your selection.

☒ Life sciences ☐ Behavioural & social sciences ☐ Ecological, evolutionary & environmental sciences

For a reference copy of the document with all sections, see nature.com/documents/nr-reporting-summary-flat.pdf

Life sciences study design

All studies must disclose on these points even when the disclosure is negative.

Sample size

No statistical sample size estimations were performed before experiments due to unknown effect sizes. Experiments on murine in vivo models were performed with 4-6 mice per condition according to internal standard procedures (cage sizes and licenses). These sample size estimates for in vivo experiments are based on expected variability and effect sizes observed for these tumor models with other treatments:

Kirchhammer, N. et al. NK cells with tissue-resident traits shape response to immunotherapy by inducing adaptive antitumor immunity. Sci. Transl. Med. 14, (2022). https://doi.org/10.1126/scitranslmed.abm9043

Läubli, H., Müller, P., D'Amico, L. et al. The multi-receptor inhibitor axitinib reverses tumor-induced immunosuppression and potentiates treatment with immune-modulatory antibodies in preclinical murine models. Cancer Immunol Immunother 67, 815–824 (2018). https://doi.org/10.1007/s00262-018-2136-x

Data exclusions

in murine models, tumors with ulcerations were excluded. For ex vivo experiments, samples without sufficient SNX9 KO were excluded (less than -0.5 log fold change in qPCR or protein staining).

Replication	For the murine model of OTI transfer to MC38-OVA bearing mice the intergenic vs Snx9 KO conditions were replicated three times which confirmed the initial results (slower tumor growth and prolonged survival with transfer of Snx9 KO OTIs). These replicates are shown in the Supplementary Figures.<br>Murine in vivo experiment transferring CART19-28z cells with or without SNX9 KO to Raji bearing NSG mice were replicated two times with similar results. The replication is shown in the Supplementary Figures.<br><br>Ex vivo results were successfully replicated with material from different individual human healthy donors (>4) in at least 1-3 (almost all experiments >2) separate experiments.
Randomization	Experimental mice were randomized according to tumor volume to ensure equal mean tumor volumes per condition at the start of treatment.
Blinding	Blinding the in vivo experiments was not feasible given the study design, the labeling requirements of the facilities and limitation of available personnel.

Reporting for specific materials, systems and methods

We require information from authors about some types of materials, experimental systems and methods used in many studies. Here, indicate whether each material, system or method listed is relevant to your study. If you are not sure if a list item applies to your research, read the appropriate section before selecting a response.

Materials & experimental systems

n/a	Involved in the study
☐	☒ Antibodies
☐	☒ Eukaryotic cell lines
☒	☐ Palaeontology and archaeology
☐	☒ Animals and other organisms
☐	☒ Human research participants
☒	☐ Clinical data
☒	☐ Dual use research of concern

Methods

n/a	Involved in the study
☒	☐ ChIP-seq
☐	☒ Flow cytometry
☒	☐ MRI-based neuroimaging

Antibodies

Antibodies used

Included in Supplementary Table 2.
Antibody target ,Target species,Fluorochrome,Provider,Catalog Nr.,Clone,Dilution
CD3,Human,PE-CF594,BD,562280,UCHT1 ,100
CCR7 ,Human,Alexa Fluor 647,Biolegend,3532218,G043H7,50
CD3 ,Human ,APC-eF780,eBioscience,47-0038-42,SK7,100
CD11b,Human ,APC,Biolegend,301310,ICRF44,100
CD4,Human ,APC,eBioscience,17-0047-42,SK3,100
CD56,Human ,APC,Miltenyi,130-113-312,REA196,100
CD45,Human,APC-H7,BD,560178,2D1 ,100
CD56,Human,BV785,BioLegend,362550,5.1H11,100
CD8,Human,FITC,eBioscience,11-0087,SK1,100
CD8,Human,BV605,Biolegend,344742,RPA-T8,50
CD8,Human,APC,Biolegend,344722,SK1,100
CD8,Human,BV711,Biolegend,344734,SK1,100
EOMES,Human,PerCP eFluor710,eBioscience,46-4877,WD1928,50
TNFa,Human,APC,eBioscience,17-7349,MAb11,20
IFNg,Human,BV421,BD,564791,4S.B3,20
KI67,Human,APC,BioLegend,350514,Ki-67,50
LAMP-1 (CD107a),Human,PE,BD,555801,H4A3 ,100
PD-1,Human,PE-Cy7,BD,561272,EH12.1 ,20
TBET,Human,BV421,BioLegend,644815,4B10,50
TIM-3,Human,BV605,BioLegend,345018,F38-2E2,50
TIM-3,Human,BV421,BioLegend,345008,F38-2E2,50
TIM-3,Human,PE,BioLegend,345006,F38-2E2,50
TCR Vbeta13.1,Human,FITC,Biolegend,362404,H131,20
TCR Vbeta13.1,Human,PE-Cy7,Biolegend,362406,H131,20
TCF7,Human,AF647,Biolegend,655203,7F11A10,20
CD28,Human,FITC,Biolegend,302906,CD28.2,50
CD28,Human,PE-Cy7,eBioscience,25-0289.42,CD28.2,50
CD57,Human,BV421,BD,563896,NK-1,100
LAG-3,Human,APC,eBioscience,17-2239-41,3DS223H,50
TOX,Human,PE,Invitrogen,12-6502-82,TXRX10,50
GZMB,Human,FITC,Biolegend,372206,QA16A02,100

KI67,Human,BV421,BD,562899,B56,50
CD45RA,Human,BV421,Biolegend,304130,HI100,100
CD4,Human,BV605,Biolegend,317438,OKT4,100
CD14,Human,BV605,Biolegend,301834,M5E2,100
CD19,Human,BV605,Biolegend,363024,SJ25C1,100
SNX9,Human,None,ThermoFisher,PA5-56734,polyclonal,500
CD8_,Human,AF488,R&D,37006,FAB1509G,100
LAMP1 (CD107a) Microscopy,Human,none,Biolegend,328602,H4A3,100
Perforin (Microscopy),Human,none,BD Pharmingen,556434,_G9,100
CD11a/CD18 (active LFA-1) (Microscopy),Human,none,Biolegend,363402,M24,100
Actin,Human,none,Sigma Aldrich,A3853-200UL,AC-40,200
NFATc2,Human,none,Cell Signaling Technologies,#5861,D43B1,800
CD45,Human,AF532,eBioscience,58-0459-42,HI30,50
CD25,Human,BV605,Biolegend,302632,BC96,100
CD69,Human,BV421,Biolegend,310930,FN50,100
CD80,Human,PE/Dazzle 594,Biolegend,305230,2D10,50
CD86,Human,BB515,BD Bioscience,564545,2331 (FUN-1),100
PD-1,Human,None,Bristol-Meyers-Squibb SA,Opdivo,"Nivolumab, clinical grade",10 mg/ml
CTLA-4,Human,None,Bristol-Meyers-Squibb SA,Yervoy,"Ipilimumab, clinical grade",10 mg/ml
pAKT (phospho-Ser473),Human,None,ThermoFisher,700392,98H9L8 rabbit,500
pPLCg1(phospho-Tyr783),Human,None,Cell Signaling Technologies,14008S,D6M9S,450
Goat-anti-mouse IgG,Mouse,AF488,ThermoFisher,A32723,"Polyclonal, cross absorbed",500
Goat-anti-mouse IgG,Mouse,AF568,ThermoFisher,A-11031,"Polyclonal, cross absorbed",200
Goat-anti-rabbit IgG,Rabbit,AF647,ThermoFisher,A21246,"Polyclonal, cross absorbed",400
Goat-anti-rabbit IgG,Rabbit,AF488,ThermoFisher,A11034,"Polyclonal, cross absorbed",500
Goat-anti-rabbit IgG,Rabbit,PE,ThermoFisher,A10542,"Polyclonal, cross absorbed",500
None,Rabbit,None,Jackson,011-000-003,Polyclonal IgG isotype control,0.1 mg/ml
Ultra-LEAF Purified anti-human CD3 ,Mouse,None,Biolegend,317326,OKT3,As indicated in figure legends
Ultra-LEAF Purified anti-human CD28,Mouse,None,Biolegend,302934,CD28.2,As indicated in figure legends
CD45.1,Mouse,BUV395,BD Biosciences,565212,A20,100
CD4,Mouse,BUV496,BD Biosciences,612952,GK1.5,100
Ly-6G,Mouse,BUV563,BD Biosciences,612921,1A8,200
NKp46,Mouse,BUV661,BD Biosciences,741678,29A14,70
CD3,Mouse,BUV805,BD Biosciences,741895,145-2C11,70
PD-L1,Mouse,BV421,Biolegend,124315,10F.9G2,150
CD8,Mouse,eFluor 450,Thermo Fisher Scientific,eBio 48-0081-82,53-6.7,100
CD45.2,Mouse,BV480,BD Biosciences,566077,104,200
MHCII,Mouse,BV510,Biolegend,107636,M5/114.15.2,300
CD80,Mouse,BV605,Biolegend,104792,16-10A1,70
CD103,Mouse,BV650,BD Biosciences,748256,2.00E+07,70
CD206,Mouse,BV711,Biolegend,141727,C068C2,100
PD-1,Mouse,BV785,Biolegend,135225,29F.1A12,100
CD19,Mouse,BB515,BD Biosciences,564509,1D3,100
CD11c,Mouse,FITC,Biolegend,117306,N418,100
Ki67,Mouse,AF532,Thermo Fisher Scientific,58-5698-82,SolA15,200
Ly-6C,Mouse,PerCP,Biolegend,128028,HK1.4,200
Tim-3,Mouse,BB700,BD Biosciences,747619,5D12/TIM-3,70
GzmB,Mouse,PE-eFluor610,Thermo Fisher Scientific,61-8898-82,NGZB,100
CD25,Mouse,PE-Cy5.5,Thermo Fisher Scientific,35-0251-82,PC61.5,100
CD28,Mouse,APC,Biolegend,102110,37.51,100
F4/80,Mouse,AF647,Biolegend,123122,BM8,100
TCF-7,Mouse,AF700,R&D Systems,FAB8224N,# 812145,
CD11b,Mouse,APC-Cy7,Biolegend,101226,M1/70,150
CD45,Mouse,V450,BD,560501,30-F11,100
CD19 ,Mouse,FITC,BD,553785,1D3,100
Chicken IgY,goat,AF488,Invitrogen,A-11039,Polyclonal affinity purified,500
GFP,Chicken,None,Abcam,Ab13970,polyclonal,1000

Validation

Antibody validation of the manufacturers were available for all antibodies including positive and negative staining controls. For the SNX9 antibody (PA5-56734) an internal knock out validation was successfully conducted (cells with and without SNX9 KO by Cas9-RNPs were stained and KOs had confirmed reduced staining with this antibody) shown in Supplementary Fig. 5a. Additional Westernblot validation of the same antibody was performed and only one major band was observed at the expected size and this band was greatly lowered by Cas9-RNP mediated KO shown in Supplementary Fig. 5b.

Eukaryotic cell lines

Policy information about cell lines

Cell line source(s)

T2 cells (ACC598, RRID:CVCL_2211) and Jurkat (ACC282, RRID:CVCL_0065) were purchased from DSMZ, Leibnitz Institute.

	HEK293T cells (ATCC CRL-3216, RRID:CVCL_0063) and Raji (ATCC CCL-86, RRID:CVCL_0511) were purchased from ATCC. The melanoma cell line NA8-Mel (RRID:CVCL_S599) generated by Dr. F. Jotereau (U211, Institut National de la Santé et de la Recherche Médicale, Nantes, France) was provided by Dr. Romero (University of Lausanne). Murine MC38-OVA colon cancer cells were provided by Mark Smyth, Peter MacCallum Cancer Centre, Melbourne, Australia.
Authentication	Cell lines were freshly ordered from reliable sources with authentication certification (DSMZ, ATCC) for T2, HEK293T and Raji. NA8-Mel and MC38-OVA were obtained from reliable academic sources. For NA8-Mel the HLA-A2+ status and absence of NY-ESO-1 endogenous expression was confirmed.<br>For MC38-OVA the OVA positivity and GFP expression was confirmed. Cell morphology matched original descriptions. No other authentication was performed.
Mycoplasma contamination	All cells were confirmed to be negative for mycoplasma by PCR as described after every freeze-thaw cycle and before injection into mice according to this protocol: Choppa, P. C., Vojdani, A., Tagle, C., Andrin, R. & Magtoto, L. Multiplex PCR for the detection of Mycoplasma fermentans, M. hominis and M. penetrans in cell cultures and blood samples of patients with chronic fatigue syndrome. Mol. Cell. Probes 12, 301–308 (1998).
Commonly misidentified lines<br>(See ICLAC register)	none

Animals and other organisms

Policy information about studies involving animals; ARRIVE guidelines recommended for reporting animal research

Laboratory animals	Wildtype (CD57BL/6NRj), OT-I (C57BL/6-Tg(TcraTcrb)1100Mjb/J, RRID:IMSR_JAX:003831), and NSG (NOD.Cg-Prkdc<scid>Il2rg<tm1Wjl>SzJ, RRID:IMSR_JAX:005557) mice were bred in-house at the University Hospital Basel, Switzerland. Animals were housed under specific pathogen-free conditions. For all experiments only female mice were used. Sex-matched littermates at 8-12weeks of age at the start of the experiments were used. Maximally allowed tumor burden of 1500mm3 was not exceeded. Mice were maintained in a sterile controlled environment (a gradual light–dark cycle with light from 7:00 to 17:00, 21–25°C, 45–65% humidity).
Wild animals	none
Field-collected samples	none
Ethics oversight	All animal experiments were performed in accordance with Swiss federal regulations and licenses (numbers 2408_34213, 2370_34209) were approved by the cantonal veterinary office (animal experimentation committee, Tierversuchskomission) of Basel-Stadt (CH).

Note that full information on the approval of the study protocol must also be provided in the manuscript.

Human research participants

Policy information about studies involving human research participants

Population characteristics	Fresh tumor tissues were collected from patients with pathologically confirmed NSCLC undergoing surgery at the University Hospital Basel, Switzerland. Median age at resection was 70.6 years, average 69.9 years (min-max: 54.6-83 years). 6 patients were male and 5 female.<br>Blood from a total of 47 different healthy human donors was used, of which 30 were male (63,83%). Median age was 47.5 years (mean = 45.1, min = 20, max = 74) Healthy donors were self-recruited potentially creating a bias for males.
Recruitment	Written informed consent was obtained from all patients and donors prior to sample collection in accordance with the Declaration of Helsinki. Tumor collection was conducted within the framework of a surgical procedure as decided and approved by an interdisciplinary tumor board. Confirmation of malignancy was obtained from a board-certified pathologist.<br><br>Healthy donors were self-recruited for whole blood donations potentially creating a bias for males.
Ethics oversight	All procedures performed in studies involving human participants were in accordance with the ethical standards of the institutional and/or national research committee (Ethikkommission Nordwestschweiz, EK321/10) and with the 1964 Helsinki declaration and its later amendments or comparable ethical standards. Informed written consent was obtained from all individual participants included in the study.

Note that full information on the approval of the study protocol must also be provided in the manuscript.

Flow Cytometry

Plots

Confirm that:

- [x] The axis labels state the marker and fluorochrome used (e.g. CD4-FITC).
- [x] The axis scales are clearly visible. Include numbers along axes only for bottom left plot of group (a 'group' is an analysis of identical markers).
- [x] All plots are contour plots with outliers or pseudocolor plots.
- [x] A numerical value for number of cells or percentage (with statistics) is provided.

Methodology

Sample preparation	At the indicated time points, T cells were stained with the following protocol. Cells are washed in PBS, resuspended in PBS, and blocked with 1:100 human Fc-receptor-inhibitor (eBioscience) in PBS and stained with Fixable Viability Dyes (Biolegend or eBioscience) 1:200 for 20min on ice. For surface staining, cells were washed and resuspended in FACS buffer (PBS supplemented with 2 mM EDTA, 0.1% Na-Azide, 2% FCS), and stained with the appropriate antibodies for 30 min at 4°C. All antibodies used in this study are listed above. For intracellular (cytoplasmic) staining, including SNX9 and cytokines, cells were fixed and permeabilized using IC Fixation Buffer (eBioscience) for 20min at room temperature. Intracellular antibodies were then stained in 1x Permeabilization buffer (eBioscience) for 30min at 4°C. For secondary staining, this procedure was repeated, including washing steps. For staining of nuclear proteins, the Fixation/Permeabilization kit (eBioscience) was used for 30min at room temperature followed by two wash cycles in 1x permeabilization buffer and antibody staining in 1x permeabilization buffer for 45 min at room temperature. We added 10'000 Precision counting beads (Biolegend) to each sample before the first washing step to adjust cell counts after acquisition based on the bead count (population high in SSC and positive in any channel <640 lasers).<br><br>For cell sorting, cells were kept on ice, washed in PBS, and stained with appropriate antibodies for 30 min at 4°C in PBS + 2% FCS and 2 mM EDTA (without Azide). Antibodies targeting CD14, CD11b, CD4 and CD19 were used to gate out potentially contaminating other immune cell populations. Following incubation, cells were washed, resuspended in the same buffer and filtered through a 70 microm mesh. Sorting of cells was performed using a FACSAria III or FACS SorpAria (BD), and the purity of sorted populations was routinely tested to be >98%.
Instrument	After staining, cells were analyzed on a BD LSR Fortessa Cell analyzer (BD Bioscience), Cytoflex S (Beckmann) flow cytometer or an Aurora Spectra Analyzer (CyTek).<br>Sorting of cells was performed using a FACSAria III or FACS SorpAria (BD).
Software	Data were collected using the BD FACS Diva Software version 7 (for Fortessa), Beckmann Culture CytExpert, or SpectraFlow (for Aurora) and further analyzed with FlowJo v10.1.6 (Tree Star Inc.) and GraphPad Prism v8 (GraphPad Software Inc.).
Cell population abundance	Sample purity after fluorescent cell sorting was confirmed by re-analysis of the sorted samples on the same machine and was >98%.
Gating strategy	Exemplary gating strategies are found in the Supplementary Figures.<br>Sorting: For the RNAseq samples and specific cells for further culture: TCRVbeta13.1+ (NY-ESO-1 TCR) CD8+ CD56- CD4- DAPI- cells<br>Analysis: Cells were always gated for non-debris (by FSC, SSC), live cells (live dead dye negative), single cells (based on linear FSC-A to FSC-H, and SSC-H to SSC-A, or FSC-A to FSC-W ratios) and CD8+ cells.<br>Gating for protein markers was done based on clear populations, negative controls (e.g. unstimulated samples for IFNg and TNFa staining), 2ndary only controls (e.g. SNX9 intracellular staining), or fluorescence minus one (FMO) controls (e.g. for Aurora spectral flow analysis. When clear populations could not be discerned, that is when intensity rather increased gradually, the geometric mean intensity for the indicated marker was used as a measurement instead (e.g. SNX9, TOX).<br><br>For the murine tumor analyses the following gating strategies were used: OTI T cells: live singlet CD19- Ly6G- CD45.2- CD45.1 + CD8+; Endogenous T cells: live singlet CD19- Ly6G- CD45.2+ CD45.1- F4/80- CD11c- CD8+ or CD4+; NK cells: live singlet CD19- Ly6G- CD45.2+ CD45.1- F4/80- CD11c- CD8- CD4- CD3- MHCII- NKp46+; cDC1: live singlet CD19- Ly6G- CD45.2+ CD45.1- CD11c+ F4/80- MHCII+ CD3- Ly6C- CD103+ CD11b-; cDC2: live singlet CD19- Ly6G- CD45.2+ CD45.1- CD11c+ F4/80- MHCII+ CD3- Ly6C- CD103- CD11b+; B cells: live singlet CD19+; Neutrophils: cDC1: live singlet CD19- Ly6G+ CD11b+; Macrophages: live singlet CD19- Ly6G- CD45.2+ CD45.1- CD11b+ F4/80+ Ly6Clow; M2 macrophages: live singlet CD19- Ly6G- CD45.2+ CD45.1- CD11b+ F4/80+ Ly6Clow CD206+; Monocytes: live singlet CD19- Ly6G- CD45.2+ CD45.1- CD11b+ F4/80low Ly6Chigh;

- [x] Tick this box to confirm that a figure exemplifying the gating strategy is provided in the Supplementary Information.
